# Statistically based splicing detection reveals neural enrichment and tissue-specific induction of circular RNA during human fetal development

**DOI:** 10.1186/s13059-015-0690-5

**Published:** 2015-06-16

**Authors:** Linda Szabo, Robert Morey, Nathan J. Palpant, Peter L. Wang, Nastaran Afari, Chuan Jiang, Mana M. Parast, Charles E. Murry, Louise C. Laurent, Julia Salzman

**Affiliations:** Stanford Department of Biochemistry and Stanford Cancer Institute, Stanford, CA USA; UC San Diego Department of Reproductive Medicine, San Diego, CA USA; Center for Cardiovascular Biology, Institute for Stem Cell and Regenerative Medicine, Departments of Pathology, Bioengineering and Medicine/Cardiology, University of Washington, Seattle, WA 98109 USA; UC San Diego Department of Pathology, San Diego, CA USA

## Abstract

**Background:**

The pervasive expression of circular RNA is a recently discovered feature of gene expression in highly diverged eukaryotes, but the functions of most circular RNAs are still unknown. Computational methods to discover and quantify circular RNA are essential. Moreover, discovering biological contexts where circular RNAs are regulated will shed light on potential functional roles they may play.

**Results:**

We present a new algorithm that increases the sensitivity and specificity of circular RNA detection by discovering and quantifying circular and linear RNA splicing events at both annotated and un-annotated exon boundaries, including intergenic regions of the genome, with high statistical confidence. Unlike approaches that rely on read count and exon homology to determine confidence in prediction of circular RNA expression, our algorithm uses a statistical approach. Using our algorithm, we unveiled striking induction of general and tissue-specific circular RNAs, including in the heart and lung, during human fetal development. We discover regions of the human fetal brain, such as the frontal cortex, with marked enrichment for genes where circular RNA isoforms are dominant.

**Conclusions:**

The vast majority of circular RNA production occurs at major spliceosome splice sites; however, we find the first examples of developmentally induced circular RNAs processed by the minor spliceosome, and an enriched propensity of minor spliceosome donors to splice into circular RNA at un-annotated, rather than annotated, exons. Together, these results suggest a potentially significant role for circular RNA in human development.

**Electronic supplementary material:**

The online version of this article (doi:10.1186/s13059-015-0690-5) contains supplementary material, which is available to authorized users.

## Background

The pervasive expression of circular RNA from protein- and non-coding loci is a recently discovered feature of highly diverged eukaryotic gene expression programs, conserved from humans to very simple organisms such as fungi [1–5]. Isolated reports of expression of circular RNAs from single genes have existed for decades, but mainly due to technological and methodological biases were generally considered to be rare splicing “mistakes” until quite recently [1]. We and others have shown that, in humans, thousands of genes have circular RNA isoforms, their expression relative to that of cognate linear RNA and their alternative splicing varying by cell type [3, 6]. For hundreds of genes, the circular RNA isoform is more abundant than linear RNA from the same locus, raising the intriguing possibility of functional roles for these molecules [1, 3, 6].

While isolated examples of circular RNA acting as microRNA sponges have been reported [2, 7], comprehensive detection and quantification of circular RNA is a necessary foundation for future studies aimed at discovery of additional circular RNA functions and elucidation of mechanisms for circular RNA regulation. Identification of biological systems in which the expression of circular RNA differs according to time, space, or cell type may provide insights into both the function and regulation of circular RNA. In order to screen large numbers of diverse datasets for this purpose, precise statistical algorithms to quantify circular and linear RNA splicing are required, and currently available algorithms for doing so have significant shortcomings. Lack of rigorous statistical testing and/or biases in ascertainment of circular RNA expression have the potential to reduce both the sensitivity and the accuracy of circular RNA detection and quantification, and can in fact lead to artifactual discovery of spurious circular RNAs. The significance of this problem was highlighted in a recent survey of circular RNA in *Drosophila*, where non-statistical alignment procedures produced millions of candidate circular RNAs which those authors believed to be dominated by artifacts [8].

High confidence reconstruction of mRNA splicing events in linear RNAs from short read RNA-Seq data remains a challenging and unsolved problem, despite advances in this area. Detection of circular RNA faces the same challenges as well as additional concerns, including systematic biases in many widely used sequencing techniques and algorithms that can lead to characteristic false positive or false negative calls. For example, some approaches will be biased against detecting very small exonic circles because read lengths exceed the length of these circular RNAs. Such an ascertainment bias might negatively impact results from genome-wide approaches to test the hypothesis that circular RNAs containing a single exon tend to be longer than other circularized exons and are therefore more efficiently circularized, as was suggested in [9].

In this paper, we present a new statistical algorithm for quantifying the expression of circular and linear RNA spliced at annotated and un-annotated exon boundaries. This new algorithm assigns confidence (a posterior probability that a junction is expressed, and a *p* value for this probability) for each detected circular or linear RNA junction from RNA-Seq experiments. It differs from other published methods in that it calculates a statistical score for each read based on alignment properties, including the number of mismatches and mapping quality. These scores are aggregated for all reads that span a putative junction to assess the strength of evidence that this junction is expressed, a concept which we previously used in a much more simple form to detect circular RNAs only at annotated exonic boundaries [3]. We demonstrate a reduction of false positive and negative results compared with other methods, and show that our improved accuracy can have significant implications for genome-wide analysis. While we focus on the methodological approach applied to circular RNA in humans in this paper, our algorithm is equally powerful and applicable to the study of linear mRNA splicing and can be applied to any genome.

We used this algorithm to rapidly test large numbers of data sets for regulation of circular RNA. Motivated by the observation that a highly expressed circular RNA in the mouse, Sry, is induced during embryonic development [10], we included data from developmental time courses to test the hypothesis that developmental induction of circular RNA could be a more general phenomenon. Our algorithm allowed us to discover striking induction of circular RNA during weeks 10–20 of human fetal development, including in the heart and lung. We found particularly high levels of circular RNA isoforms in the developing brain, including in genes essential to neurogenesis, such as lncRNA RMST [11], adding to the recent report that circular RNA is enriched in the aging fly brain [8]. We have shown that induction of circular RNA in the heart is recapitulated in in vitro directed differentiation of human pluripotent stem cells to the cardiomyocyte lineage and focus on the circular RNA from the gene NCX1, a calcium transporter that is essential for cardiac development [12, 13]. The developmental induction of the circular RNA from NCX1 is conserved at least between human and mouse, suggesting that developmental regulation of circular RNA may be evolutionarily conserved. In addition to these results, our algorithm unveiled circular RNAs that use splice sites not annotated in the current genome databases, including some spliced by the U12 (minor) spliceosome that likely use splice sites that are not used for splicing of the linear mRNA.

## Results

### Statistical annotation-dependent algorithm

**Fig. 1:**
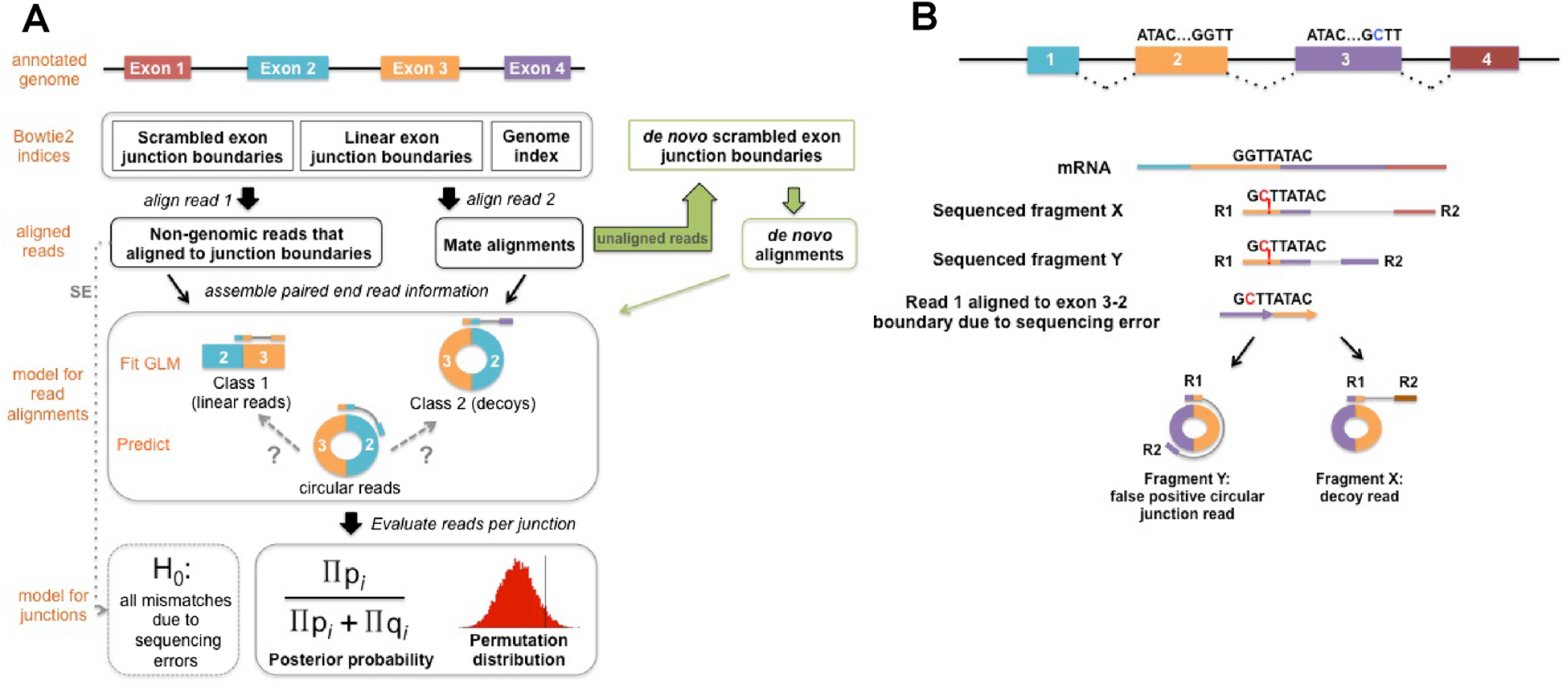
Computational pipeline to identify circular RNA candidates. **a** We start with an annotated genome to create a database of junction sequences which is used to create two custom Bowtie2 junction indices: 1) a scrambled junction index containing all possible junction sequences formed either from circularization of a single exon or by each pair of exons in non-canonical order within a 1 Mb sliding window; 2) a linear junction index containing these exon pairs in canonical order. For single-end data (*SE*), mismatch rates in all reads aligned to a given junction are used to determine whether it is a true or false positive (see “Materials and methods”). For paired-end RNA-Seq data, read 1 (*R1*) and read 2 (*R2*) are aligned independently as single-end reads to these junction indices and a Bowtie2 genome index. Each R1 that aligned to a scrambled junction and did not align to the genome or a linear junction is categorized based on its mate alignment: circular if the mate aligns within the genomic region of the presumed circle defined by the junctional exons or decoy if the mate aligns outside this region. Each R1 that aligned to a linear junction is categorized as linear if the mate aligns concordantly to support a linear transcript. Reads in the linear and decoy categories are used to fit a generalized linear model (*GLM*). The GLM predicts the probability that each circular read belongs to class 1 (true positive) versus class 2 (false positive). The de novo analysis pipeline is shown in *green*. All reads that did not align to any of the indices are used to create a Bowtie2 index of de novo junction sequences, and all of the unaligned reads are re-aligned to this index. Each R1 from the de novo alignment is categorized based on its mate alignment just as the reads aligned to annotated exon junctions are. See “Materials and methods” for details. **b** Sequencing errors can lead to incorrect alignment to a circular junction. This results in either a false positive circular read or decoy read depending on whether R2 aligns inside or outside of the circularized region defined by R1

Our analysis pipeline (Fig. 1a) uses statistical modeling to improve sensitivity and specificity of circular RNA detection from RNA-Seq data. For paired-end data, we map each read independently to separate Bowtie2 indexes for the genome, ribosomal RNA, linear exon–exon junctions, and scrambled exon–exon junctions. Each junction index contains all exon pairs within a one megabase sliding window. For a read to be considered “junctional”, read 1 (R1) must overlap the junction by a user-specified number of nucleotides and must not have a high-scoring alignment to the genome or ribosomal indexes. If a read has a high-scoring alignment to both a linear junction and scrambled junction, only the linear junction alignment is considered. We model false positives in a sample using two categories of reads: 1) those that map to canonical linear isoforms (“real” alignments); and 2) those which are likely artifacts because the relative alignment orientations of the paired-end reads are consistent with neither a linear nor a circular RNA (“decoy” alignments). As recently reported, these decoy alignments are not on their own sufficient to discriminate true positive circular RNA from false positives, and are typically simply removed from analyses [8].

Decoy reads could result from a convolution of sequencing errors and homology between genes as described in [14]. For example, many exons in the human genome derive from ALU elements and hence have very similar sequences, leading to genes for which two or more exons in a single gene are distinguished by only a handful of nucleotides [15]. As depicted in Fig. 1b, if read 1 of a paired-end read truly originates from an exon 2–exon 3 linear junction and exons 2 and 3 are nearly identical, sequencing and/or polymerase errors may cause this read to best map to an exon 3–exon 2 circular junction. If read 2 maps to exon 4, as shown for fragment X, the read would be put into the “decoy” category because the read pair is incompatible with a circular or linear transcript. However, if read 2 mapped into exon 3, as shown for fragment Y, it would not be flagged as a decoy as the relative orientations of the reads would be consistent with a circular RNA. Therefore, due to a combination of sequencing errors and exon homology, such a read would result in a false positive observation of an exon 3–exon 2 circle. This is an example of what we subsequently refer to as “artifacts” or “false positives”. Other processes that give rise to decoy reads include technical artifacts such as template switching during the reverse transcription step of RNA-Seq library construction [16].

The above models for the production of decoy reads suggest that sequencing base quality, the number of mismatches in an alignment, the “uniqueness” of alignment (mapping quality) and the offset (the number of nucleotides that overlap the junction boundary) should jointly predict the likelihood that a read is a decoy. Decoy reads, considered as a group, would be predicted to generally have smaller offsets and worse mapping quality and alignment scores compared with other classes of reads. We therefore built a statistical model, described below, to predict whether a read is a decoy based on these variables. Specifically, rather than ignoring decoy reads, we assigned them to a bucket (class 2) and compared them with reads consistent with originating from linear mRNA (class 1). We fit a logistic generalized linear model (GLM) with three predictors — alignment score (a composite of sequencing quality and mismatch rate), mapping quality, and offset position — to the response variable, which is the binary set of class labels (class 1 or 2 above). Because of the systematic differences in biochemistry and read quality between read 1 and read 2 on Illumina platforms, particularly for random-primed libraries, we only considered read 1 in assigning a paired-end read to class 1 or 2 [17, 18]. This makes our counts of junction expression comparable to roughly half the number that are reported by algorithms that use both reads to quantify junction expression.

The GLM was fit for each dataset, with the larger class downweighted so that each class had equal influence in the model. All reads within a class were initially weighted equally. After fitting this model, the weights of reads with poor fit to the model were downweighted in proportion to their lack of fit, maintaining the constraint that, together, reads in each class had equal total weight (see “Materials and methods” for details). The model was then fit again to the dataset using the modified weights to obtain the final estimates of the three coefficients in the GLM and their statistical significance. We used this model to predict the probability of class membership in the independent set of reads with paired-end alignments consistent with being generated from circular RNA. The fit of the model and significance of each predictor is data-dependent, but in all data sets evaluated here, each predictor was highly significant, which corresponds to a significant improvement of model fit by including it [19].

The next step of our algorithm was also novel compared with other methods: rather than treating each read independently, we borrowed strength across all reads aligning to the junction and formalized quantification of each putative junction via Bayesian hypothesis testing. For each junction, we computed the posterior probability that junction-spanning reads, in aggregate, had a statistical profile consistent with decoy reads, which would suggest they are artifacts versus those from linear splice junctions, which would suggest they are real [20]. The posterior probability is the conditional probability that a junction is a true positive conditioned on one of two scenarios: reads from the junction are all decoys or all true positives. The posterior probability thus reduces to:

$$ {P}_i = \frac{\prod \widehat{p}}{\prod \widehat{p}+\prod \left(1-\widehat{p}\right)} $$

where the product is over all non-decoy reads aligned to the junction. See Fig. 1 and “Materials and methods” for workflow and statistical details of the algorithm. Intuitively, if half of the reads from a given junction have statistical profiles consistent with artifacts while the other half are consistent with a true circular isoform, the posterior probability of this junction would be 50 %. We were most interested in circular junctions with a strong level of support, and hence only focused on junctions with high posterior probabilities exceeding 90 %, corresponding to a very small fraction of reads with statistical similarity to the profile of decoy reads (see “Materials and methods”).

### Posterior probability of circular RNA expression is independent of read count

**Fig. 2:**
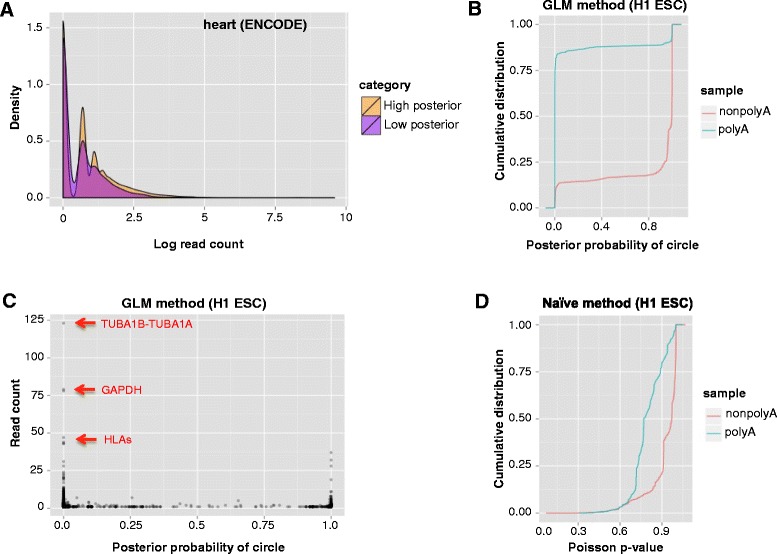
Statistical algorithm improves the precision of circular RNA detection. **a** Circular junctions with at least one aligned read are divided into two groups based on posterior probabilities: greater than 0.5 (*orange*); 0.5 or less (*purple*). Density distributions of the read counts are shown for each group in a representative ENCODE tissue sample (adult heart). **b** Cumulative distribution of posterior probability for circular RNA detected (read count ≥ 1) in poly(A) + and poly(A)- ENCODE H1 human embryonic stem cell line. Higher posterior probability indicates increased confidence that a circle is a true positive versus an artifact. **c** Putative circles with the highest read counts (labeled in *red*) in the H1 poly(A) + sample are identified as false positives. **d** Cumulative distribution of *p* value calculated based on the naïve model described in “Materials and methods”, using the same paired-end data used in panel (b). Higher *p* value indicates increased confidence that a circle is a true positive versus an artifact

As an initial orthogonal evaluation to determine whether our approach provides information that cannot be obtained based on read count alone, we assessed the relationship between the posterior probability assigned to a circular junction and the number of reads aligned to the junction. Analysis of multiple datasets (see Fig. 2a for a representative sample) shows that the level of confidence is not merely a function of read count.

We also control for the effect of total junctional counts on posterior probability for linear junctions by computing the distribution of posterior probability per junction, conditioning on total junctional counts and the distribution of predicted ${\widehat{p}}_i$ across all linear reads. This is necessary because our GLM classifier is trained on linear junctional reads and in principle could assign a very high predicted probability of being “real” to all reads from a linear junction [19]. To control for this, after estimating the predicted probabilities of each read, we compute a *p* value for the posterior probability of a junction based on the null hypothesis which induces a permutation distribution on the $\left\{{\widehat{p}}_i\right\}$, the distribution that randomly assigns a ${\widehat{p}}_i$ for each read independent of the junction to which the read aligned. The permutation distribution on $\left\{{\widehat{p}}_i\right\}$ induces a distribution on the posterior probability for a linear junction as a function of number of aligned reads (see “Materials and methods”). We perform an analogous test for each circular junction with a simple model to provide a false discovery rate (FDR) because the above approach is too conservative for circular RNA junctions, as these reads were not used to fit the GLM model (see “Materials and methods”).

Unlike existing methods, which generate a list of circular RNAs that are prioritized based on read counts, our approach is based on calculating a statistical confidence score for each junction with aligned reads. This is particularly important given that, like long non-coding RNAs, and some essential genes such as Smoothened (supplemental file for accession GSE64283), the vast majority of circular junctions (70–80 %) detected in RNA-Seq data have fewer than five aligned reads. This holds even when experimental procedures to enrich for circular RNA, such as RNase-R, are used (Additional file 1). This is due to the low abundance of many circular RNAs as well as the fact that some circular RNAs are sensitive to RNAase-R [5, 6, 21]. While we have focused our analyses and discussion on circular RNA using deep sequencing data with high coverage of most linear junctions, in shallow sequencing experiments both circular and linear junctions can have low coverage and still be statistically significant.

### Statistical model decreases false positive identification of highly expressed circular RNA

To further evaluate our statistical approach, we performed a global assessment of the performance of our algorithm in the RNA-Seq data for poly(A) + and poly(A)- fractions of multiple cell lines generated by the ENCODE consortium. Although poly(A) + libraries are greatly enriched for RNA with poly(A) tails, a small number of RNAs that lack poly(A) tails, such as small nucleolar RNAs (snoRNAs), are still present. For example, in RNA-Seq data from H9 embryonic stem cells (ESCs), although snoRNA is depleted during poly(A) selection as expected (25 % of snoRNAs have an average coverage of 0.6 or less in poly(A) + libraries compared with an average coverage of 1 or less in poly(A)- and of 2 or less in RNase-R+ libraries), some snoRNA are still readily detected in poly(A) + samples (25 % of snoRNA have an average coverage of 3.9 or more compared with 6.3 or more in poly(A)- and 31.9 or more in RNase-R+ libraries) (see “Materials and methods”; Additional file 2). As exemplified by this survey of snoRNA in RNA-Seq data generated from different library preparations of the same cell line, biochemical methods used to enrich for specific types of RNA significantly alter the composition of RNA sequenced, but do not result in a purified sample containing only the RNAs of interest.

Assessing circular RNA expression in poly(A)+/− datasets, compared with benchmarking against RNase-R-treated RNA-Seq, allows a completely orthogonal approach to assessing computational methodology. As our previous work [1] and the above assessment of snoRNAs show, most, but not all, apparent circular junctions in poly(A) + libraries are likely to be artifacts of the type described in our discussion of decoys, as circular RNAs lack poly(A) tails, and are generally not purified by poly(A) selection. Under this model, purely computational methods should detect very different expression levels of circular RNA in the two types of libraries [1, 6]. Our model, blinded to whether input data were poly(A) selected or depleted, flagged approximately 90 % of circles with mapped reads in the poly(A) + data as false positives (Fig. 2b). At the same time, the model was able to identify circular RNAs that were not completely depleted in the poly(A) + data; as expected, these true positives were less abundant than the false positives. Examples include circular RNAs that have been validated in other cell lines, such as RNF220, HIPK3 and MAN1A2 in HeLa cells [1], CRKL in H9 ESCs [22], LRP6 and SPECC1 in 293 T cells [2], and FAT3 [3].

Examples of the effectiveness of our approach in identifying false positive junctions with many mapped reads are depicted in Fig. 2c. GAPDH is not expected to express a circular isoform as it is sensitive to RNase-R [2]. Also, in the absence of statistical checks, previous reports have excluded putative circles from paralogous genes such as TUBA1A/1B and HLA genes as being false positives based on heuristics rather than quantitative modeling [6, 23]. Our algorithm detects these false positives without manual ad hoc filtering.

Another example is highlighted in the analysis of our own RNA-Seq data from fetal heart tissue introduced below. In multiple samples, a putative circle formed by splicing MYH6 exon 7 to MYH7 exon 8 appears to be one of the two most highly expressed circular RNAs, and is a putative circle predicted by find_circ [2]. MYH6 and MYH7 are highly homologous myosin heavy chain proteins, both of which are highly expressed in the heart and essential for cardiac function. Gene homology, convolved with sequencing errors, could cause some reads from linear junctions in MYH6 to have a better match to junctions comprised of one exon from MYH6 and one from MYH7. Our method predicted that MYH6-MYH7 is a false positive, despite its apparently high expression level. As orthogonal support, more than one-third of the reads aligned to this junction in each sample were decoys which were not considered in the calculation of the posterior probability of the putative MYH6-MYH7 circle, but supported the conclusion that it is a false positive due to the high homology between the two genes. Furthermore, linear isoforms of MYH6 and MYH7 were readily detected by PCR in our hands, while several attempts to amplify a MYH6-MYH7 junction all gave negative results.

We have also compared our method to a naïve method to calculate the per-junction *p* value for having the observed number of mismatches in the reads aligned to that junction under the null hypothesis that all mismatches are due to sequencing errors [14]. Given this null, a low *p* value represents a junction identified as a false positive while those with high *p* values represent junctions identified as true positives. As demonstrated by comparing the results of applying both methods to the ENCODE poly(A) + and poly(A)- RNA-Seq data for H1 ESCs (Fig. 2b, d), the naïve method provides *p* values that can be useful in flagging likely false positive junctions, which is our algorithm’s default when only single-end data are available, but our GLM approach provided much stronger discrimination.

### Statistical algorithm increases sensitivity of circular RNA detection compared with published algorithms and influences genome-wide analyses

**Fig. 3:**
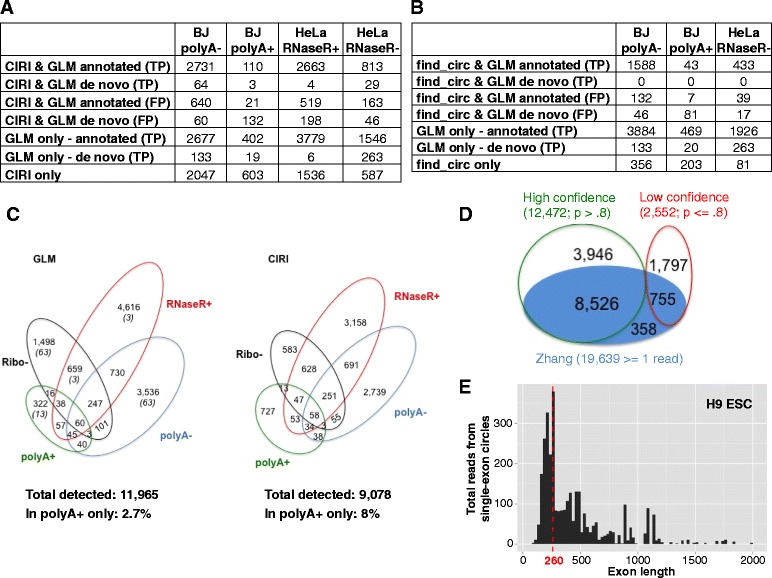
Statistical algorithm improves the sensitivity of circular RNA detection. **a, b** Circular RNA detected by both algorithms are divided into false positives (*FP*; flagged as false positives due to low posterior probability) or true positives (*TP*; our posterior probability ≥ 0.9). **a** Number of circular RNAs detected by our GLM or CIRI in ENCODE BJ poly(A)+/− data and HeLa RNase-R+/− data generated by Gao et al. [23]. CIRI results are based on all default parameters except the -E flag set to exclude false positives resulting from identical colinear exons. **b** Number of circular RNAs detected by our GLM or find_circ in ENCODE BJ poly(A)+/− data and HeLa RNase-R- data generated by Gao et al. [23]. **c** Circular RNAs detected in HeLa RNase-R+ and Ribo- data generated by Gao et al. [23] and poly(A)+, and poly(A)- data generated by ENCODE. Number of circular RNAs detected by our GLM method (one or more reads, posterior probability ≥ 0.9) compared with CIRI (default parameters except -E). For GLM results, the first number is the total number of circles and the number of those which were detected by the de novo portion of the algorithm are listed in parentheses. **d** Venn diagram comparing the number of putative circular RNAs identified by our annotation-dependent algorithm in Rnase-R-treated H9 cells and the results published by Zhang et al. [22]. *Green circles* and *red circles* show circular RNA identified by our algorithm with high and low confidence, respectively; the *blue circle* shows those identified by Zhang et al. **e** Total junctional reads for circles comprised of a single exon (posterior probability ≥ 0.9, read count > 1) shown by size for same data as in panel (d). Median exon length is shown in *red*. The x-axis is truncated at 2000 excluding 31 long exons, all but one with total read counts < 50

To evaluate the sensitivity of our approach, we compared the number of circles detected in ENCODE poly(A) + and poly(A)- RNA-Seq data as well as RNase-R-treated RNA-Seq data generated in [23] with the number detected by the de novo circular RNA algorithms CIRI [23] and find_circ [2] (Fig. 3a–c; Additional file 1). We ran CIRI v.1.2 with default parameters except for adding the -E flag to exclude false positives resulting from identical colinear exons. We used default parameters for find_circ and followed recommendations in the README file provided with the source code to filter results to obtain high quality circles. For our algorithm, we reported circles comprised of annotated exons with at least one junctional read and a posterior probability of 0.9 or higher. Since our counts reflect only cases where read 1 aligned to the junction, a threshold of 1one junctional read for our algorithm is comparable to a threshold of two junctional reads for algorithms that count read 1 and read 2 from paired-end reads. Additionally, as pointed out in [24], counting both reads could result in double counting individual reads if both sides of a read are junctional, as would be expected for long reads and small inserts or small circles. In reporting circles detected by our de novo algorithm, we applied the filters described in “Materials and methods” that were used for all subsequent analyses.

In data from HeLa RNA treated with RNase-R to enrich for circular RNA, we detected approximately 30 % more circles than CIRI, and comparable levels in poly(A)- data from BJ cells. However, in the RNase-R- HeLa cells, we detected an additional 984 circular RNAs, 1.6 times the number of circles reported by CIRI. Although these 984 circular RNAs were depleted by RNase-R, it has been demonstrated by PCR that some circular RNAs are particularly sensitive to RNase-R, although certainly much less sensitive than linear mRNA [2, 8, 25]. CIRI found almost twice as many circular RNA as our algorithm in the BJ poly(A) + sample, but many of these additional findings were likely false positives given that circular RNA which lack poly(A) tails are generally not purified by poly(A) selection. Of note, 100 circles detected only by CIRI had 50 or more aligned reads, many of which were in highly homologous genes, such as the HLA and collagen genes, further suggesting that they may have been false positives (Additional file 1).

As reported in [23], find_circ detected fewer circles than CIRI in all samples tested. In the poly(A)- and RNase-R- samples, our algorithm detected over 2000 more circles than find_circ. We received errors when attempting to run find_circ on the RNase-R+ HeLa sample, and so were unable to directly compare the algorithms on these data. Taken together, these results support the idea that our balanced use of annotations and de novo discovery is more sensitive than these strictly de novo approaches.

In order to assess whether our increased sensitivity is accompanied by an increase in detection of likely false positive circular RNA, we quantified the overlap of circular RNA predicted in poly(A)+, poly(A)-, ribosomal RNA-depleted, and RNase-R-treated libraries generated from HeLa cells (Fig. 3c; Additional file 1). Our GLM algorithm reported 11,965 high confidence circular RNAs. As expected, the largest number were found in the sample treated with RNase-R, which should be enriched for circular RNA, while the fewest were found in the poly(A) + sample enriched for RNA with poly(A) tails. This result is not due to sequencing depth, as the number of mapped reads in the poly(A) + sample was more than twice the number of mapped reads in the RNase-R+ sample. In comparison, CIRI detects only 75 % of the circular RNAs detected by our GLM method in the same dataset: 9078 circular RNAs in the HeLa cells, reporting fewer circular RNAs than our algorithm in each of the samples except in the poly(A) + sample. We repeated this comparison using single-end data from poly(A)+, poly(A)- and RNase-R+ libraries from H9 ESCs and found that our naïve algorithm also matches predictions based on the method of biochemical purification better than CIRI (Additional file 3). Finally, the GLM algorithm developed in this paper also represents a great improvement over our previously published algorithms [1, 3], increasing sensitivity by 40–50 % with a negligible change (~2 %) in the rate of false positive detection (Additional file 4).

We also analyzed the human H9 ESC data used by [22] to benchmark their circular RNA detection method, which they found to be more sensitive than MapSplice [5] and comparable to Segemehl [26]. In this dataset, we detected a total of 15,024 circles, of which 12,472 were high-confidence, compared with 9639 circles with at least one read identified by the authors [22]. There were 9281 circles detected by both algorithms, with 3946 additional high-confidence circles detected by our algorithm and 358 circles only detected by their algorithm, of which only 152 had more than one read (Fig. 3d; Additional file 5).

We tested whether these improvements in detection sensitivity could have implications for inferences regarding mechanisms underlying species-specific circular RNA production by considering two examples highlighted in [22]: 1) that circularized exons are larger than average exon sizes in humans; and 2) that presence or absence of repeats can predict whether circular RNA production occurs in orthologous genes in human and mouse.

Previous analyses suggest that short exons are less likely to be circularized [6, 22]. We considered the possibility that this observation may reflect a size bias in both experimental techniques and existing informatics pipelines for the detection of circular RNA. By minimizing size bias at the alignment step by using junctional reference sequences of the same length regardless of flanking exon length (see “Materials and methods”), we found evidence of numerous small, circularized exons. For example, in H9 human ESCs we found that the median length of circularized exons with a high posterior probability of being a true circle was 260 nucleotides (Fig. 3e; number of circular RNAs by exon length shown in Additional file 6). When we applied a threshold based on read count instead of posterior probability to select high-confidence circles, the median length of circularized exons was much larger. Consistent with a recent report [22], we observed that using a threshold of five or more reads resulted in a median circularized exon length of 353 in H9 cells. This may be due to small circles being prevalent but detected at low levels due to selection for fragments > 200 nucleotides during sample preparation, and excluded from analyses that impose hard filters on read counts. The distribution of lengths of circularized exons was highly similar in all tissues and cell types examined, suggesting that circles form more readily from smaller exons than previously hypothesized. Our analysis is consistent with the true expression level of these small circles being higher than currently estimated, resulting in an overestimation of the true median length of circular RNA comprised of a single exon.

Testing the extent of evolutionary conservation between circular RNA production in orthologous genes has the potential to uncover mechanisms underlying circular RNA biogenesis and to infer function of these molecules [1, 3, 4, 6, 22]. Examples where an orthologous gene in human and mouse produces circular RNA in only one of the species may yield insights into the mechanism of its production [22]. For example, Zhang et al. [22] found that while mouse ESCs had detectable levels of circular RNA from the gene Zwilch, a circular isoform was not detected in humans. Conversely, human H9 ESCs had detectable levels of circular POLR2A whereas this isoform was not detected in mouse, and a mechanistic explanation for these observations was suggested [22].

In contrast to this previous report, our algorithm identified expression of circular RNA from Polr2a in mouse ESCs, including in exons lacking flanking inverted repeats (Additional file 7). Using the same RNA-Seq data as analyzed by [22], we also detected and validated circular isoforms from the human ZWILCH gene at the comparable, and low, levels expressed in mouse ESCs, although none were the exon 6–exon 7 circle homologous to the circle detected in mouse [22]. We have also detected many circular isoforms of ZWILCH across larger ENCODE datasets (results available in the supplemental file of accession GSE64283). More study will be necessary to test whether the newly detected circular isoforms of ZWILCH are compatible with the model put forth by [22] because, for example, in ENCODE poly(A)- mononuclear cells, we detected a circular isoform of ZWILCH which lacks flanking inverted repeats.

### Statistical de novo assembly identifies significantly expressed circular RNAs

To expand methods to quantify circular RNA, we also developed a statistical algorithm to discover un-annotated splice sites used in circular RNA production. If circular RNAs use splice sites distinct from those used in mRNA, they may be missed using annotations, as most annotated splicing boundaries are based on cDNA libraries derived from poly(A) selected RNA [23]. Furthermore, exon annotations for most genomes are much lower quality than the highly scrutinized human genome; in such organisms, circle ascertainment using annotations will be correspondingly poorer. We sought to test this directly by developing a statistical algorithm that analyzes reads that fail to align to the genome or any annotated splice site (“unaligned” reads).

In its first step, the algorithm proceeds like many previously published methods: splitting each unaligned read into two parts and aligning each part separately to the genome. The algorithm then selects reads where the two parts aligned to the genome on the same strand, but in a relative orientation inconsistent with a linear RNA splice. Next, the algorithm bins the genome into non-overlapping 50-nucleotide bins and computes a list of all pairs of bins (A, B) where two separate fragments of the same read have aligned. For each such pair, the algorithm compiles all sequences which have split reads aligning to the bin pair (A, B) and performs a statistical test of whether these reads are consistent with being independent realizations of a single splicing event, producing a score statistic (see “Materials and methods”). To reduce false positives (for example, due to reverse transcription [RT] artifacts), we do not consider bin pairs where the statistic suggests that multiple distinct breakpoints/putative splices have occurred. This step of the algorithm also produces a consensus sequence for the putative splice represented by each (A, B) bin pair. In the final step of the algorithm, we align all unaligned reads to a Bowtie2 index containing these de novo junction consensus sequences and use the framework developed for our annotation-dependent analysis to classify reads that align to a de novo junction into putative circles or decoys and assign confidence to each junction (Fig. 1), with a slight modification (see “Materials and methods”).

Using this de novo method, we detected 38 of the junctions missed by our annotation-dependent algorithm when comparing our results with [22] on their human H9 ESC data (Fig. 3d), including all with more than 13 reads and all but 13 with 5 or more reads reported by [22] (Additional file 5). Our algorithm also unveiled exons that were annotated subsequent to the creation of our comprehensive exon–exon database, or were clear incomplete annotations of the human genome. For example, we discovered and validated a circular isoform of the gene RMST that was not present in our curated list of annotated splice junctions because the UCSC annotation available at the time contained an RMST isoform extending only to position 97,927,544 while the circle is formed from a recently annotated exon further downstream. In addition, by manual inspection, we found examples of circular isoforms using exons that were annotated in expressed sequence tags (ESTs) but not included in curated gene models (Additional files 8 and 9; gel shown in Additional file 10; data for RNase-R sensitivity in Additional file 11).

### Un-annotated splice sites are enriched for canonical U2 exon boundaries

To benchmark our algorithm, we examined the global distribution of the dinucleotides flanking the backspliced junctions identified by our de novo pipeline. This distribution is an unbiased assessment of its ability to discover splicing events as, importantly, generation of candidates has no bias to discover splicing events at canonical U2 “GT-AG” boundaries. However, in cases where the breakpoint of a circular junction read was ambiguous, we assigned it to a “GT-AG” boundary (or analogous consensus for the U12 spliceosome; see below) if such existed. We focused our analysis on ENCODE tissue data (ENCODE accession GSE24565), and found a strong enrichment for the consensus motifs that flank canonical introns recognized by the major spliceosome: “GT” at the 5′ end of the intron and “AG” at the 3′ end. After re-alignment to the de novo junctional database (see “Materials and methods”), ~70 % of junctions were annotated as having canonical flanking nucleotides (supplemental file in accession GSE64283), as defined by consensus motifs of the major or minor spliceosome [27], and similar results were found in all other datasets we analyzed. Others are likely a combination of artifacts, lariat junctions and possibly bona fide splicing events.

### Global induction of circular RNA during fetal development

Complex alternative splicing is a hallmark of multicellular development. For example, a handful of splicing regulators are known to control essential alternative splicing programs in the brain and heart [28–31]. To test whether regulated circular RNA splicing is a feature of early developmental gene expression programs, we tested rRNA-depleted RNA-Seq libraries across a panel of human fetal tissues sampled at developmental time points ranging from 10 to 21 weeks of development (Additional file 12) and used our statistical algorithm to detect linear and circular splicing events in these data. The most complete developmental time courses were available for the heart and lung.

We used our algorithm’s quantitative estimate of junctional splice expression and a posterior probability of the confidence of this estimate to identify circular and linear RNAs that were expressed with high confidence (see “Materials and methods”; accession GSE64283), and subsequently used these quantitative estimates to test for evidence of circular RNA induction during fetal development. Each circular RNA is uniquely identified by its “backsplice” and its expression is therefore estimated by counting reads aligned to this diagnostic junctional sequence. Since linear splice junctions contained within a circle could be present in circular or linear isoforms, we considered only linear splice junctions strictly outside of the boundaries defined by the circular backsplice (“exterior” splices) for the purpose of quantifying linear splicing expression. In cases with multiple circular RNAs from a single gene, we took the intersection of all exterior splices to define the composite “exterior” category.

**Fig. 4:**
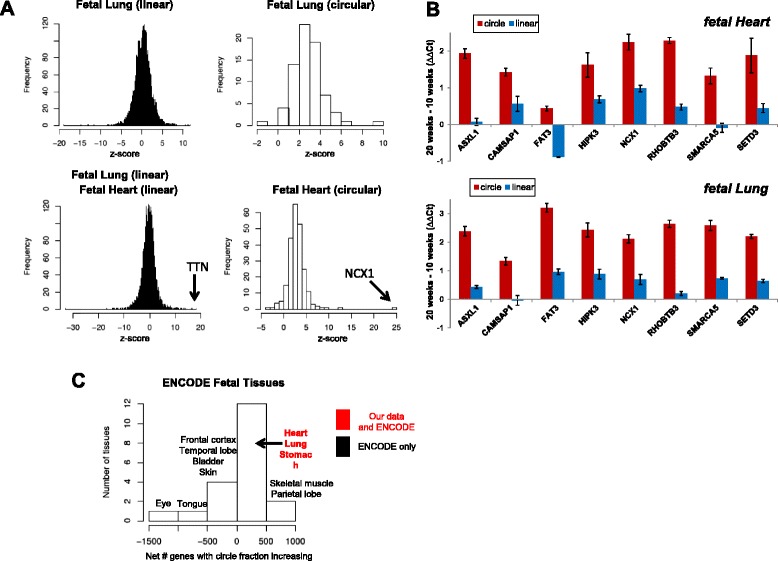
Circular RNAs are induced during development. **a** Genome-wide distributions of z scores for linear and circular junctions in our heart and lung data show significant skewing of z scores in circular junctions towards positive values corresponding to circular RNA induction. **b** Quantitative RT-PCR confirms greater induction of circular RNA in several organs; heart and lung are shown here (intestine and stomach in Additional file 14). Plotted values are ΔΔCt = ΔCt(Age 20 weeks) – ΔCt(Age 10 weeks), where ΔCt = Ct(ACTB) – Ct(target); error-bars are standard error of the mean of technical replicates. Positive ΔΔCt indicates increased expression later in development, and is log2 scale. **c** A similar trend is seen in the ENCODE data: 14 out of 20 tissues, including heart and lung, have a majority of genes with increasing circular:linear expression compared with decreasing circular:linear expression (genes called with *p* < 0.05). Net # genes with circle fraction increasing is defined as Number of genes with circle fraction increasing from early to late timepoint – Number of genes with circle fraction decreasing. Tissues not labeled in the figure contained in the 0–500 bar are spinal cord, thyroid, metanephros, liver, umbilical cord, occipital lobe, cerebellum, diencephalon, uterus (all with data only available from ENCODE)

For each organ and each gene with a circular isoform(s), we derived a z score for each circular splice and each cognate exterior category of linear splices (Additional file 13). At a fixed sampling depth, this z score is expected to reach its maximum when junctional reads (corrected for sequencing depth per library) increase linearly as a function of fetal age, and should be distributed as a standard normal under a model where there is no relationship between expression and age. As expected from theory, the z score captures variable expression of linear junctions with age and is roughly normally distributed (see Fig. 4a left panels for representative examples), with outliers in well-known developmentally induced genes, such as TTN in the heart, serving as positive controls. Note that the size of a z score represents a combination of effect size and sample size: for example, NCX1 had the highest z score among circular RNA in the fetal heart (Fig. 4a lower right panel) because its expression level is high and therefore very well-sampled, resulting in high statistical confidence that it increases over time.

Compared with linear RNA junctions, circular RNA junctions in the heart and lung display a strong bias towards positive z scores, representing relative induction over time (Fig. 4a right panels); similar induction is seen in the intestine and stomach (Additional file 14). This trend can be readily seen in the developing heart where virtually all z scores are positive, even if they do not reach statistical significance at this sequencing depth. Quantitative PCR (qPCR) validated our RNA-Seq predictions (Fig. 4b; Additional files 9, 15 and 16), and in most cases induced circular RNA expression cannot be explained by the level of induction of the mRNA, as measured by z scores. To check the robustness of this finding, we repeated this analysis using the subset of linear junctions with the same donor (or acceptor) as used in the circular junction but that splice downstream (or upstream) rather than “back”. Results were very similar (Additional file 17).

### Independent datasets corroborate global induction of circular RNA during fetal development

To examine the generality of the results from our fetal data set, we performed a comparable experiment on ENCODE data collected from fetal samples at similar developmental times as our own data (splice quantification results in supplemental file of accession GSE64283). The ENCODE RNA-Seq data were, like our own, produced from ribosomal-depleted RNA, and allow us to survey a wider array of tissues for evidence of circular RNA induction during fetal development. However, the ENCODE data do not have as much temporal sampling as our data; for most organs, there are only two developmental time points, collected between 2 and 10 weeks apart (Additional file 18); hence, z scores cannot be used to evaluate temporal induction.

We used another statistical test to evaluate induction of circular RNA in these data: for each gene, we quantified the maximum expression of any circular splice (C) and the maximum expression of any exterior mRNA splice (E). We quantified the number of genes where the ratio of circular RNA expression compared with mRNA was higher (*p* < 0.05) in the later developmental time point compared with the earlier time point using binomial confidence intervals for the ratio of C/(E + C), which intrinsically normalizes for sequencing depth (see “Materials and methods”). Fourteen of the 20 tissues, including all of the organs in which we identified global circular induction in our own dataset, exhibited an increase in the ratio of circular RNA to linear mRNA over developmental time in at least 50 % of genes, although some other tissues available only in the ENCODE data exhibited the opposite trend (Fig. 4c).

### Dominant circular RNAs are expressed in a tissue-specific manner and enriched in the brain

**Fig. 5:**
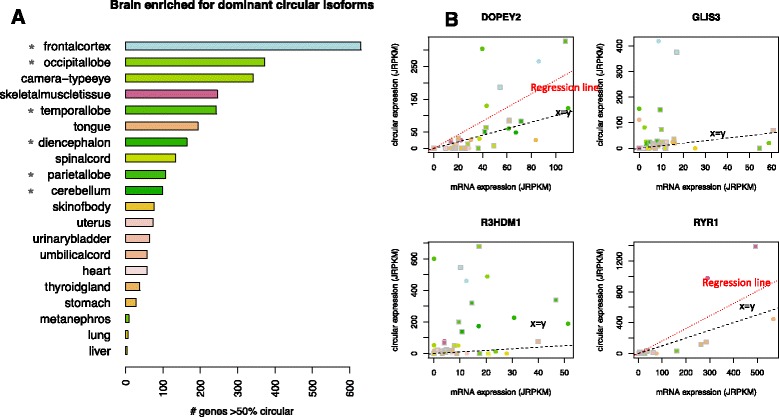
Circular RNAs have high abundance in many tissues and tissue-specific expression programs. **a** In many fetal tissues, especially regions of the brain, hundreds of genes have dominant circular isoforms in early and late time point samples. Late time point depicted for simplicity: for each organ, the total number of genes with greater circular RNA compared with linear RNA is plotted (*p* < 0.05). Asterisks indicate regions of the brain. **b** Many genes with tissue-specific increases in expression are also more highly expressed as circular compared with linear isoforms. Normalized expression levels from two samples, early (*circles*) and late time points (*squares*), in four genes illustrate this phenomenon (see “Materials and methods”). Statistically significant outliers (*p* < 0.001) include several subregions of the brain (DOPEY2 and the RNA binding protein R3HDM1), the frontal cortex (GLIS3) and skeletal muscle (RYR1); regression line (*red*) is plotted if there is a significant relationship with circular expression; x = y line plotted in *black*. JRPKM (junctional reads per kilobase per read mapped) have a comparable interpretation to RPKM (see “Materials and methods”)

We used the ENCODE data to test whether the increased abundance of circular RNA over time was driven by induction of linear RNA, particularly whether the most highly expressed circular isoforms were positively correlated with the cognate linear isoforms and whether they had tissue-specific expression. First, for each sample, we quantified the number of genes where circular RNA was estimated to exceed linear RNA (estimated by the exterior category). Under the assumption that at least one splice junction is constitutive in each linear transcript, this will be a fair estimate of circular RNA:mRNA expression, if not an underestimate. These estimators revealed hundreds of genes with higher expression of circular RNA compared with linear RNA in many fetal tissues (Fig. 5a), including genes essential for development, such as NCX1 in the heart, and genes with genetic links to severe neurodevelopmental phenotypes such as RMST and FBXW7 [11, 12, 32, 33]. RNA-Seq quantification was orthogonally supported by qPCR (Additional files 9, 15, and 16; data showing RNase-R resistance in Additional file 11). The enrichment of circular RNA in the brain was not due to simply detecting larger total numbers of genes or splicing events as demonstrated by computing the total number of such events in each ENCODE dataset (supplemental file for accession GSE64283).

We also tested for a relationship across all samples between the level of circular:linear isoform expression, which is intrinsically normalized for sequencing depth, and the level of linear isoform expression normalized to the total number of reads mapping to linear splice junctions. Specifically, for the 100 genes having the highest levels of circular RNA expression across all datasets, we computed the fraction of circular counts C/(C + E), and tested for a significant relationship with normalized exterior linear count expression (E) using regression (see “Materials and methods”). The majority of genes (77) did not have a significant regression coefficient (*p* < 0.001), suggesting a general lack of a predictive relationship between circular and linear expression, as previously reported in cell lines [1, 3, 5, 6].

Even when a regression coefficient is not significant, residuals from regression analysis can be used to assess whether the variation in the dependent variable, here circular RNA expression, is greater than can be explained by “noise” (see “Materials and methods”). We used such a residual analysis to identify genes with a higher level of circular RNA expression in a sample than would be predicted based on linear isoform levels or noise alone (outliers). Using a conservative FDR of 0.001, we found 30 of the above 100 genes with two or more statistically significant tissue outliers, including 19 genes where both the early and late samples from the same tissue were outliers, indicating tissue-specific expression (see Fig. 5b for representative examples, all of which have higher circular RNA expression than linear). DOPEY2 had the second largest number of outliers, all in subregions of the brain; R3DHM1, an RNA-binding protein, also had consistent outliers in the brain. Other examples of tissue-specific outliers detected in this analysis included skeletal muscle samples with outliers in the gene RYR1, responsible for calcium extrusion in skeletal muscle, and samples in the frontal cortex in the gene GLIS3. Many of the outliers were detected in the frontal cortex and temporal and occipital lobes (Additional file 19).

### Expression of NCX1 circular RNA increases more rapidly than linear RNA during fetal heart development

**Fig. 6:**
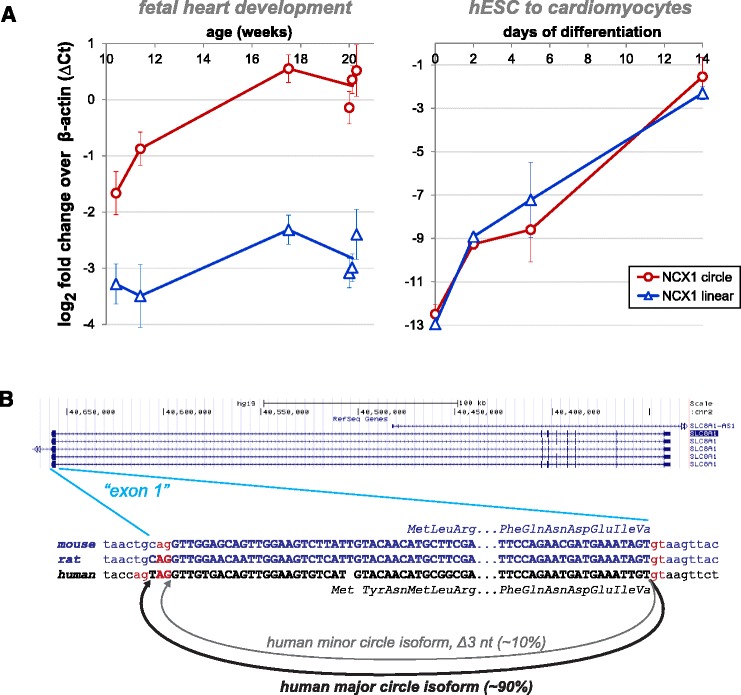
NCX1 is a highly expressed and conserved circular RNA. **a** qPCR agrees with sequencing estimates and shows that circular isoforms of NCX1 are induced in the fetal heart and during in vitro cardiomyocyte differentiation. Plotted values are ΔCt = Ct(ACTB) – Ct(target); error bars are standard error of the mean of technical replicates for fetal heart, and of biological triplicates for human ESC (*hESC*) to cardiomyocytes. **b** Our de novo sequencing algorithm predicted a minor circular isoform differing by a deletion of three nucleotides from the dominant circular isoform; it arises from use of a splice-acceptor just downstream of the annotated splice-acceptor. The minor circular isoform was confirmed by PCR and clone sequencing. In the diagram, exonic sequences from genome annotations are given in bold uppercase, and splice-signal dinucleotides are highlighted in red; the mouse and rat NCX1 sequences are shown in blue. In the rat, the NCX1 circular isoform was only detected with the aid of our de novo algorithm, as the circle junction does not coincide with the annotated start of the first exon. Notably, in the mouse the start of this exon is annotated as exactly where we see circular splicing in the rat and mouse

While the human fetal brain has the most remarkable level of total circular RNA expression, the single gene with the highest level of developmental induction of a circular RNA and highest expression across all of our RNA-Seq data was the dominant circular isoform of NCX1 in the heart. NCX1 is a sodium/calcium exchanger responsible for transporting calcium out of the cardiomyocyte after contraction [34]. We observed a consistent increase in circular RNA production from this locus in the heart across all developmental time points, in both our own datasets (Fig. 6a) and in the ENCODE dataset (supplemental file for accession GSE64283).

The dominant circular isoform of NCX1 contains a single exon encompassing the boundary between the 5′ untranslated region (UTR) and translation start site and is expressed at roughly two to five times the level of the linear isoform. Using our de novo algorithm, we also identified a highly expressed circular RNA differing from the dominant isoform by a three nucleotide deletion at the backsplice junction, which is present at roughly 10 % of the level of the major circular isoform and is also induced during development (Fig. 6b). We validated both of these predicted events by TOPO cloning and PCR (Additional file 10).

### Human ESC-derived cardiomyocyte differentiation recapitulates induction of expression of circular and linear NCX1 RNAs

Human tissues are comprised of a mixture of different cell types. The remarkable abundance of the circular isoform of NCX1 in the fetal heart samples suggests that it must be highly expressed in the majority of developing heart cells, and that its induction might be recapitulated by in vitro directed differentiation of human ESCs to cardiomyocytes. We tested this by harvesting human ESCs at sequential stages of differentiation — undifferentiated (day 0), mesoderm (day 2), cardiac progenitor (day 5) and definitive cardiomyocyte (day 14) — although these cells still lack some gene expression characteristics of fetal cardiomyocytes. We performed RNA sequencing in biological triplicate and the same analysis to test for temporal induction of circular RNA as we did for our human fetal samples (Additional files 20 and 21).

The gene with the most significant z score in this time course of cardiac induction coincided with the most significant one in the human fetal data from the heart: NCX1. The second most significant z scores in the ESC-derived cardiomyocytes and heart also coincided, corresponding to the gene RHOBTB3. Both of these circles are expressed at high levels, comparable to the level of beta-actin by day 15 (NCX1 shown in Fig. 6a).

### Evolutionary conservation of circular NCX1 expression discovered with de novo algorithm

While human genomes are extensively annotated, the genomes of other organisms have not been as well studied, and splicing and/or gene models are not complete. In order to assess evolutionary conservation of circular RNA splicing, we require accurate unbiased identification of circular isoform expression. We specifically focused on NCX1, as a broader study of evolutionary conservation is beyond the scope of this work. Our pipeline that quantifies circular RNA at annotated exon boundaries identified a circular isoform of NCX1 (Fig. 6b) as the most highly expressed circular RNA in mouse heart RNA-Seq data (SRA:SRP029464). The circle was also induced in development, suggesting that upregulation of the circular isoform of NCX1 is evolutionarily conserved. Our de novo pipeline identified expression of NCX1 circular RNA in the rat heart (SRA:SRP037986; Fig. 6b) mirroring circular RNA expression in human and mouse (circular:linear ratio of 0.23). The 5′ acceptor splice site identified by our algorithm is flanked by a canonical U2 splice site motif, but is not present in current gene models, explaining why our annotation-dependent pipeline failed to identify this circle. This evidence points to an incomplete annotation of the rat NCX1 gene model and demonstrates the importance of using an annotation-independent algorithm when assessing evolutionary conservation of circular RNA expression.

### Un-annotated human circular RNAs are highly expressed at cryptic splice sites

To expand our search for expression of developmentally regulated circular RNA, we also used our de novo statistical algorithm to test for un-annotated splice sites used in circular RNA production in human fetal samples. In total, we identified more than 300 circular RNA splicing events using either an un-annotated donor or acceptor in the ENCODE fetal tissue samples (ENCODE accession GSE24565). These junctions had a similar distribution of expression to those using annotated splice sites (Additional file 22). More than 65 circular RNAs identified by this de novo pipeline have more than 50 reads compared with roughly 2700 such circular RNAs which use canonical splice sites, increasing the number of highly expressed circular RNAs by ~2 %. We validated five out of five de novo junctions predicted from the ENCODE data in our own samples (Additional files 10 and 11).

Several examples of circular isoforms reported by our de novo pipeline involved splicing events that used donors or acceptors very close (i.e., < 10 nucleotides) to annotated splice sites, and flanked by canonical U2 dinucleotides. This phenomenon has been reported as a mechanism to expand the proteome through mRNA splicing [35], but to our knowledge has not been described for circular RNA. One of these includes a splice of the gene NCX1 (Fig. 6b). Estimates of the prevalence of this isoform from both RNA-Seq and qPCR suggest that roughly 10 % of the molecules of NCX1 circular RNA are comprised of this variant.

### U12 circular RNAs are spliced at un-annotated boundaries, some developmentally induced

The U12, or minor, spliceosome is highly evolutionarily diverged from the U2 spliceosome and splices a small minority of human genes [27]. However, U12 splicing is known to be essential for development in some metazoans and its disruption causes developmental disorders in humans [36–38]. Because of its evolutionary divergence from the U2 spliceosome, and because only roughly 800 human genes are known to have a U12 intron and only five have more than one, U12-dependent circular RNA expression is not expected and has not been previously identified [6]. We identified a group of roughly 60 circular splicing events using a U12 annotated donor and acceptor in the ENCODE dataset supported by small numbers of reads (Additional file 23), compared with the 636 unique linear splice events at annotated U12 junctions (Additional file 24).

**Fig. 7:**
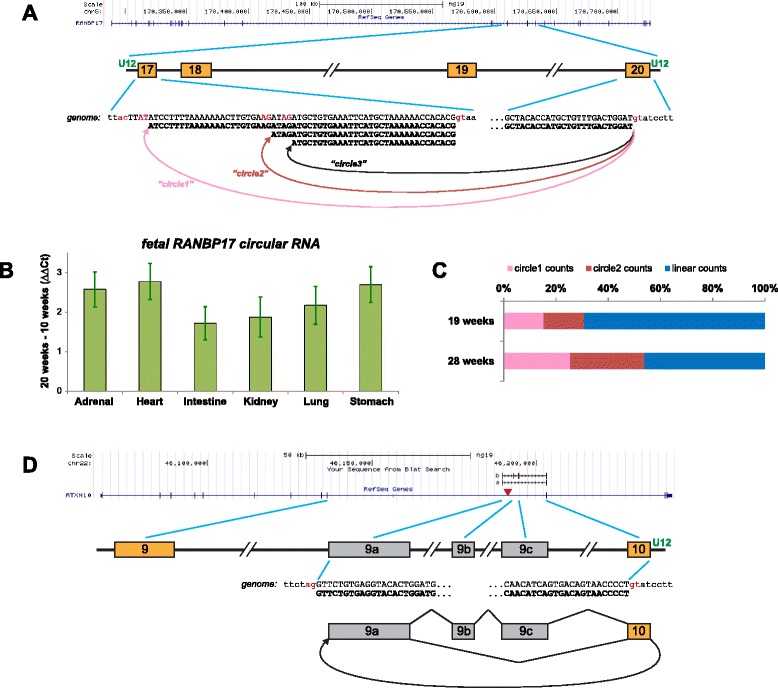
U12 circular RNA has tissue-specific increases in development. In the gene diagrams of (a, d), annotated exons are shown as *gold boxes*, un-annotated “cryptic” exons as *gray boxes*. Definitive U12-type introns are indicated by “*U12*” in *green*; other introns are U2-type (or possibly cryptic U12-type). Splice-signal dinucleotides are shown in *red*. **a** Our de novo algorithm identified two circular isoforms in RANBP17 that use the U12-type splice signal following exon 20; these were validated by PCR and clone sequencing (which also identified the third isoform shown). **b** By RT-qPCR, the de novo RANBP17 circular isoforms show induction during fetal development in all tissues examined; the expression varies between tissues, for example, being significantly higher in the heart than the intestine. Values plotted are ΔΔCt = ΔCt(20 weeks) – ΔCt(10 weeks), where ΔCt = Ct(ACTB) – Ct(RANBP17); error bars are standard error of the mean of technical replicates. **c** The fraction of RANBP17 transcripts that are circular isoforms increases over developmental time. Shown are the percentages of each RANBP17 isoform, based on RNA-Seq junctional reads, at two different time points in fetal heart development. “*circle1*” = chr5:170632616:170610174, “*circle2*” = chr5:170632616 :170610198 (hg19 genome junctional coordinates; the third circle was not included in this analysis). Total junctional read counts were 240 and 267 for 19 and 28 week samples, respectively. **d** The de novo algorithm identified a circular junction in ATXN10, between the U12-type splice signal following exon 10 to a specific site within intron 9. PCR and clone sequencing with outward-facing primers in exon 10 verified the junction and also showed that additional un-annotated exonic sequences also form part of these circular isoforms, which show alternative splicing. Pathological expansion of a short repeat within intron 9 is a genetic hallmark of spinocerebellar ataxia type 10; the repeat region, marked with a *red triangle*, lies close to exonic sequences that we have identified as contributing to the circle

However, analysis of U12 splicing with our de novo pipeline in the ENCODE data revealed a group of 17 predicted U12-dependent circular RNAs that use at least one splice site not currently annotated as exonic. The U12 circular RNAs using un-annotated splice sites were four-fold more highly expressed than U12 circular RNA at annotated exons. In some cases, U12 acceptors found by the de novo pipeline were in very close proximity to the mRNA transcript in the gene, such as in RANBP17 (Fig. 7a). Validation of these U12 circular isoforms of RANBP17, as well as others (Additional files 10 and 11), showed even more diverse splicing than we detected by our algorithm; for example, we only predicted two of three RANBP17 splicing variants found by PCR. Validation also confirmed induction of circular isoform expression, which we predicted in several genes, with circular isoform levels comparable to linear by 28 weeks (RANBP17 shown in Fig. 7c). We also detected a U12 circular RNA in the gene ATXN10 backspliced from exon 10 to a cryptic exon in the preceding intron (Fig. 7d); a repeat expansion in this intron is the causative genetic change responsible for spinocerebral ataxia type 10 [39].

## Discussion and conclusions

In this paper we have developed a new statistical algorithm for detecting and quantifying circular RNA and applied this algorithm to discover new biology of circular RNA expression. Our methods constitute a contribution to quantification of splicing events from RNA-Seq data by assigning statistical confidence to the expression of each junctional sequence by which we can identify both false positive junctional expression and improve detection sensitivity. Our approach to de novo detection of circular RNA is conceptually different from previous approaches that treat these events as binary without an associated statistical confidence. While we focus on applying this algorithm to discover circular RNA, we anticipate that it will be widely applicable to a range of experimental questions being addressed by RNA-Seq. The methods are also applicable to linear RNA splicing and we have used them to discover and validate novel linear splicing events (data not shown). We believe further algorithmic development along the conceptual lines we developed in this paper will lead to more powerful inferences from sequencing studies.

Given the wide variety of RNA isolation and library preparation techniques used and the biases inherent in each [18], many RNA isoforms will be underrepresented in any given RNA-Seq experiment. Therefore, informatics analysis of such data must be able to distinguish between noise and true reads present at low levels. Our algorithm is agnostic to library preparation method, and, as predicted from biochemical rationale, revealed significantly different posterior probability distributions of putative circular RNA detected in poly(A) + versus poly(A)- data despite being blinded to the data input type. Importantly, the increased sensitivity of our algorithm for isoforms that are small and/or present at low levels is not accompanied by an increase in false positives, suggesting that our algorithm can be applied widely to libraries generated by diverse biochemical methods. Highlighting the importance of statistical approaches to circular RNA detection, we discovered and validated examples of circular RNA missed by other algorithms, and demonstrated that such results can have important implications for models of circular RNA biogenesis.

Our new methods allowed us to rapidly screen diverse datasets and identify biological contexts where circular RNA is regulated. We discovered global and specific examples of circular RNA variation over human fetal development. The global trend of increasing circular RNA expression during fetal development points to the possibility of up-regulation of a *trans*-acting factor that generally promotes circular RNA expression. Another possible explanation for this phenomenon is that circular RNA degradation is a slow process, and, therefore, as cells become quiescent and post-mitotic, they accumulate circular RNA while maintaining relatively constant levels of mRNA. A trivial explanation for our finding of developmental induction of circular RNA could be derived from sample handling, resulting in older samples having a bias towards ascertaining circular RNA at a higher rate than linear RNA. In principle, this could be due to, for example, higher activity of endogenous endonucleases in early fetal samples. However, our orthogonal tests did not support this hypothesis, as we were able to reproduce our findings in the ENCODE datasets and recapitulate induction of circular RNAs such as NCX1 and RHOBTB3 in the heart using an in vitro system where induced pluripotent stem cells are differentiated into cardiomyocytes.

There is increasing evidence that circular RNA is involved in biochemical pathways, including acting as miRNA sponges [2, 7] and binding proteins [2, 40]. This suggests that there could be functional consequences of developmental variations in levels of circular RNA, especially in the brain where circular RNAs constitute the dominant isoform of hundreds of genes. Uncovering a previously unknown landscape of circular RNA production has important implications for future work studying mechanisms of circular RNA biogenesis and its function. As we have shown that circular RNAs can be spliced to cryptic exons and these circles can accumulate to levels comparable to linear mRNAs, it may be that genomic instability could trigger cryptic splicing of circular RNA. It is tempting to speculate that, in the case of ATXN10, the expanded repeat (a form of genomic instability) in diseased patients is contained in some circular RNA isoforms, especially considering the aggregation of repeat-containing RNA in the cytoplasm of patient-derived cell lines without any detectable change in mRNA levels [39].

Considering our results that circular RNAs are induced during human fetal development, and a recent study in *Drosophila* that suggests accumulation of circular RNA during aging [8], developmentally timed expression may be a conserved feature of circular RNA expression. If circular RNA continues to accumulate during late life in post-mitotic cells in long-lived organisms like humans, we hypothesize two testable explanations: 1) a higher rate of production compared with degradation of circular RNA, resulting in a consistent increase in circular RNA over time, or 2) regulation of *trans*-acting factors controlling the production or degradation as an organism ages.

Developmental regulation of circular RNA could potentially function to change protein–protein interactions (scaffolded by RNA) or the kinetics of bound protein. Either of these hypothetical functions could be important in development as gene expression programs change rapidly. Our finding of dynamic expression of circular RNA during development is a first step toward experiments that may reveal functions of circular RNA: it has already led us to find in vitro differentiation systems that recapitulate circular RNA expression over time and highlighted circular RNAs which we are now studying in the development of cardiac and neural lineages.

## Materials and methods

### Fetal sample processing and RNA-Seq data generation

Fetal tissue samples were collected under a protocol approved by the UCSD Institutional Review Board. Fetal tissue samples <100 mg in size were placed into DNase- and RNase-free 1.5 ml microfuge tubes containing 1 ml of RNAlater RNA Stabilization Reagent (Qiagen) within 1 h of the pregnancy termination procedure. After storage at room temperature for a period of 24–72 h, excess RNAlater was removed from the microfuge tubes and the samples were placed in the −80 °C freezer for storage until RNA isolation was performed. The tissues were lysed in mirVana (Life Technologies) lysis buffer, using a Mini-Beadbeater-16 (Biospec), with agitation for 1 min in the presence of 1 mm zirconia beads. Samples were then centrifuged at maximum speed in a tabletop microcentrifuge for 1 min and the lysed solution was transferred to a fresh microfuge tube. The remainder of the extraction was per the manufacturer’s protocol for the mirVana kit (Life Technologies). After extraction, RNA was quantified using the Quant-iT RNA BR Assay Kit (Life Technologies). RNA quality was assessed using the Agilent 2100 Bioanalyzer (Agilent) using RNA Nano-chips.

Following RNA isolation (mirVana miRNA Isolation Kit, Life Technologies, Inc.), the RNA was quantified (Qubit RNA Assay Kit, Life Technologies, Inc.), and quality controlled (RNA6000 Nano Kit and BioAnalyzer 2100, Agilent). We used 200–1000 ng as input for the Illumina TruSeq Stranded Total RNA with Ribo-Zero Gold sample prep kit (Illumina, Inc.) and sequencing libraries were created according to the manufacturer’s protocol. Briefly, first both cytoplasmic and mitochondrial rRNA was removed by selectively hybridizing biotinylated probes to target sequences and using magnetic beads to capture the bound products. Following rRNA removal, the RNA was fragmented and copied into first strand cDNA using random primers and reverse transcriptase. Next, second strand cDNA synthesis was completed using DNA polymerase I and RNase H. The cDNA was then ligated to Illumina supplied adapters and enriched by PCR to create the final cDNA libraries. The libraries were then pooled and sequenced on a HiSeq 2000 (Illumina, Inc.) instrument as per the manufacturer’s instructions. Sequencing was performed up to 2 × 101 cycles. Data are available under accession GSE64283.

### RT-PCR, qPCR, and Sanger sequencing

cDNA synthesis was done with random hexamers and M-MLV Reverse Transcriptase RNase H Minus Point Mutant (Promega or NEB) with the following program: 25 °C for 10 min, 42 °C for 50 min, 45 °C for 5 min, 50 °C for 5 min, 85 °C for 5 min, 4 °C hold. Pre-treatment with RNase-R followed the protocol in [4]. cDNA reactions were diluted with water and used as template for PCR. Standard PCR was done with Taq or Phusion DNA polymerase (New England Biolabs); qPCR was done with GreenStar qPCR Mix (Bioneer, with ROX added to final 500 nM) on an ABI 7900HT using the default program. PCR primers are listed in Additional file 17. PCR products were either Sanger-sequenced directly using the amplification primers, or cloned into a TOPO vector (Invitrogen/Life Technologies) and sequenced with vector primers. Gel images of PCR products used for cloning are shown in Additional file 10 and RNase-R resistance of circular isoforms is presented in Additional file 11.

### Data used

#### ENCODE cell lines

Raw fastq files available on 8 August 2014 were downloaded from the ENCODE project website. We selected all whole-cell long poly(A)- and poly(A) + reads banked at [41]. At that time, two poly(A) + replicates from each of 18 cell types were available, with the exceptions that one HMEC, three NHEK, and four SKNSHRA replicates were downloadable. Two poly(A)- replicates from each of 17 cell types were available, with exceptions that one HMEC and three NHEK replicates were downloadable. Read 1 and read 2 reflect directionality of original RNA and were not processed symmetrically.

#### ENCODE tissue (GSE24565)

Raw fastq files available on 9 October 2014 were downloaded from [42]. We selected paired-end RNA-Seq for human tissue. At that time, one sample from each of two biologic replicates was available for 22 tissues, with exceptions that one mononuclear cell and three heart samples were downloadable.

#### Rat tissue (SRP037986)

Raw fastq files available on 1 November 2014 were downloaded from the Sequence Read Archive (SRA). There were two to four technical replicates per sample and we selected the run that generated the most reads for each sample. All 32 samples available for adrenal, brain, heart, kidney, liver, lung, and muscle, and the 16 available for uterus were downloaded.

#### Mouse heart tissue (SRP029464)

Raw fastq files for ribo-minus experiments available on 12 December 2014 for ventricle tissues were downloaded from SRA. One sample from each of five time points ranging from postnatal day 1 to postnatal day 90 were available.

#### Mouse R1 ESC (SRR1552726)

The raw fastq file for poly(A)- RNA available on 6 December 2014 was downloaded from SRA.

#### Human H9 ESC (SRR901967)

The raw fastq file for poly(A)-/RNase-R-treated RNA available on 8 December 2014 was downloaded from SRA.

#### HeLa RNase-R+/−

The raw fastq files under SRR1637089 and SRR1637090 were downloaded from SRA on 28 January 2015.

### Data processing

Raw fastq files were processed using TrimGalore [43] version 0.3.7 and cutAdapt [44] version 1.5 to remove adapter sequences and trim poor-quality bases. As for all RNA-Seq analysis tools, our algorithm may perform poorly on low quality data. Therefore, we strongly recommend using our algorithm only on reads that have been processed by a tool such as cutAdapt as predictions will be adversely affected by low-quality reads. All default values were used, with the exceptions that the length parameter was set to exclude reads where either mate had a trimmed length of less than 50 and the phred64 flag was passed for the ENCODE cell lines.

### Annotation-based junction index design and Bowtie2 alignments

We start with an annotated genome (hg19 UCSC annotation downloaded from [45]) to create a database of junction sequences. Each junction sequence is comprised of 150 bases from each of the two exons (or the 3′ and 5′ ends of a single exon), and shorter exons are padded with Ns so that all junctions have a length of 300, thereby reducing the bias against finding circles comprised of small exons. We include sequences representing circularization of a single exon as well as all pairwise combinations of exons within a sliding window of one million bases across each chromosome. We use the subset of canonical-order junctions from this database to build a Bowtie2 [46] index of linear isoforms and the remaining junctions are used to build a Bowtie2 index of scrambled isoforms. We use Bowtie2.2.2 to align reads to the genome, linear, and scrambled indices simultaneously. We impose a high gap penalty in order to obtain only ungapped alignments and set the minimum score threshold to allow an equivalent of up to four mismatches at high-quality positions per 100 bases in a read. Because short exons are padded with Ns in the junction indices, we also reduce the N-penalty to 0.0001 when aligning to the junction indices. Naïve application of Bowtie2 using the default N penalty of 1 prevents alignment of simulated reads originating from genes with short exons to these small junction sequences, whereas completely removing the N penalty creates a bias toward the small junctions. Even a small N penalty results in a slight bias toward longer exons, as Bowtie2’s behavior in this context is to preferentially align to the longest possible junctional sequence, but we found the chosen N penalty mitigates the bias for or against small exons. Default values are used for all other parameters.

### GLM algorithm for detecting circular junctions in paired-end RNA-Seq

We align the mate reads from a paired-end RNA-Seq experiment independently to the genome index, linear junction index, and scrambled junction index as if they were single-end reads and subsequently aggregate the mate information for all diagnostic reads, those where read 1 (R1) aligned across a junctional sequence. To be conservative, diagnostic reads where R1 also aligned to the genome are discarded, and if R1 aligned to both a scrambled junction and a linear junction, it is assigned to the linear junction only. Similarly, if read 2 (R2) aligns to more than one of the indices, we select the one with the highest Bowtie2 alignment score and in the case of ties we give preference to the genomic alignment, then the linear junction, and finally the scrambled junction. Based on the alignment position of its mate (R2), each diagnostic read is categorized as linear, circular, or decoy. Linear reads are those where R1 aligns to the linear junction index and R2 aligns concordantly in the genome or linear junction index to support a linear transcript. All reads where R1 aligns to the scrambled index are categorized as circular if R2 aligns within the genomic region of the presumed circle defined by the junctional exons (with a buffer of 15 nucleotides to account for potential technical artifacts introduced during RT), or decoy if R2 aligns outside of this region. We allow for the possibility that the presumed circle may include or exclude any introns and exons within the genomic coordinates involved in the junction.

In the absence of a ground truth, we proceeded with the premise that the large majority of linear reads most likely represent true alignments and the decoy reads are more likely to be false alignments due to either sequencing or alignment errors. Supporting the validity of this premise, we do observe that the linear reads tend to have higher alignment scores (indicating fewer mismatches) and higher mapping qualities (meaning Bowtie2 assigns higher confidence to the reported alignment) compared with the decoy reads. We also observed a difference in the distribution of the number of nucleotides in a junction-aligned read that extend across the junction boundary. The distribution of these alignment features for the two categories of reads in a representative sample is shown in Additional file 25.

We build a statistical model for each sample that combines the information contained in these three alignment features to estimate the probability that each read potentially representing a circular RNA is a true alignment, as opposed to an artifactual alignment, using a logistic GLM. We use this model to achieve an estimated probability that each read *i* is a correct alignment (*Y*_*i*_ = 1) or an artifact (*Y*_*i*_ = 2). Using a binomial distribution with probability $P\left({Y}_i=1\right) = {\widehat{p}}_i$, this probability is modeled with predictors being the number of nucleotides in the read that overlap the junction, the Bowtie2 alignment score, and the Bowtie2 mapping quality. Since read 2 is used to categorize each read as linear, circular, or decoy, we only use the value of these predictors from read 1 and fit the model with linear reads as class 1 and decoy reads as class 2. Because there are likely some errors in our category assignments (some reads in the linear category that are actually artifacts and some decoy reads that are actually true alignments), the model is fit in two steps. In the first step, all of the reads within a category are given equal weight. The linear category is generally much larger than the decoy category, so the reads are weighted such that the sum of the weights in each category is equal. In the second step, the weight of each read, *w*_*i*_, is adjusted by an amount proportional to the probability predicted by the GLM in step 1, ${\widehat{p}}_i$, as follows: the weight of the *i*^*th*^ read in the linear category, ${w}_i=\frac{{\widehat{p}}_i}{{\displaystyle \sum {\widehat{p}}_j}}$ for all linear reads j, and the weight of the *i*^*th*^ read in the decoy category, ${w}_i=\frac{{\widehat{p}}_i}{{\displaystyle \sum {\widehat{p}}_j}}$ for all decoy reads j. The result is that reads are downweighted if the category assignment based on genomic coordinates of the mates conflicts with that of the GLM category assignment. Specifically, linear reads with ${\widehat{p}}_i$ close to 0 receive less weight than linear reads with ${\widehat{p}}_i$ close to 1, and conversely decoy reads with ${\widehat{p}}_i$ close to 0 are given more weight than decoy reads with ${\widehat{p}}_i$ close to 1, but the sum of weights within a class remains equal.

The model is then fit using these updated weights and this final model is used to predict ${\widehat{p}}_i$, the probability that each circular or linear read is a true positive alignment. The per-read probabilities are then used to calculate a posterior probability that each junction is truly expressed based on the cumulative evidence from ${\widehat{p}}_i$ for all reads aligning to the junction:

$$ P = \frac{{\displaystyle \prod \widehat{p}i}}{{\displaystyle \prod \widehat{p}i+}{\displaystyle \prod \left(1-\widehat{p}i\right)}} $$

To be conservative, we use the lower bound of the 95 % confidence interval on ${\widehat{p}}_i$ for reads in class 1 and the upper bound of the 95 % confidence interval on ${\widehat{p}}_i$ for reads in class 2.

### *p* value calculation for linear junction posterior probabilities

As outlined in the text, we compute a *p* value for the posterior probability based on the null hypothesis that the probability ${\widehat{p}}_i$ computed for each read by the model is independent of the junction to which the read aligned by a permutation distribution that randomly assigns reads to junctions. This controls for the possibility that junctions with many reads are more likely to have extreme (high or low) posterior probabilities due to chance and allows us to detect circular and linear splice events with high confidence across the broad range of expression levels. For a junction with n reads, the posterior probability *P*_*n*_ is a function of:

$$ {y}_n={\displaystyle \prod \frac{\left(1 - {p}_i\right)}{p_i}} $$

where the product is over *p*_*i*_ for each read as predicted from fitting the GLM, and thereby a *p* value computed for *y*_*n*_ or log(*y*_*n*_) is equivalent to a *p* value for *P*_*n*_. We use this transformation so the empirical variance can be obtained in closed form. We compute the null distribution for each *l*_*n*_ = log(*y*_*n*_) as follows: a normalized z-score is computed as:

$$ {z}_n = \frac{l_n-E\left({l}_n\right)}{\sqrt{var\left({l}_n\right)}} $$

where the mean and variance are calculated from the data with *E*(*l*_*n*_) = *n* ∗ *μ* and *μ* is the empirical average of the random variable *l*_*n*_ across all decoys and linear junctions and *var*(*l*_*n*_) is the empirical variance of *l*_*n*_. Since *z*_*n*_ is normally distributed under the null hypothesis, we report a *p* value based on a one-sided test on this z score that is low if the posterior probability that the junction is truly expressed is higher than expected by chance.

### *p* value and FDR calculation for circular junction posterior probabilities

For circular junctions, the *p* value as calculated for linear junctions is overly conservative, since the GLM predicts on data that was not used in fitting the GLM. Thus, for circular junctions, we use a simple model to estimate the *p* value (and Benjamini-Hochberg corrected FDR) for each junction. Under this model, we assume a null model that if a junction is an artifact, with 95 % probability, a read will have a predicted probability of 0.1, and with 5 % probability (due to chance), it will have a predicted probability of 0.9. Using this model, we compute a z score and *p* value as described above where:

$$ \mu = \log \left(\frac{.9}{.1}\right) $$ and $$ \operatorname{var}\left({l}_n\right)=\mathrm{nlog}\left(\frac{.9}{.1}\right)\hat{\mkern6mu} 2 $$.

The resulting *p* value is conservatively computed using the minimum posterior probability for each junction to be considered in our report processing: 0.9. Using this lower bound for all posterior probabilities allows us to estimate a conservative Benjamini-Hochberg FDR which compares the *i*^*th*^ smallest *p* value to $\frac{i}{total\  junctions\  tested}$.

### Naive algorithm for detecting junctions

We model the null hypothesis that all mismatches are due to sequencing error using Poisson(0.01) for Illumina reads. We sum the number of mismatches observed in all reads aligned to a junction and calculate the *p* value for observing this many or fewer mismatches given the total number of bases in the aligned reads for this junction. This method is used by our pipeline when only single-end RNA-Seq data are available, and for de novo discovery.

### GLM report processing

Junctions with a posterior probability exceeding *p* and at least *n* reads were carried forward in the analysis and are reported in Additional file 13 and the processed reports available under accession GSE64283 for our fetal data and ENCODE fetal data. For our fetal data, *n* = 2; for the ENCODE data, *n* = 2; for cardiomyocyte reports, *n* = 1; *p* = 0.9 for all datasets.

### Algorithm for detecting de novo junctions

Reads that failed to align to any reference in the annotated circular RNA pipeline were culled for further analysis. In this paper, we only consider R1 for building the candidate list of novel junctions. To do this, each R1 was aligned to the genome using Bowtie version 0.12.7 in two segments via a ‘split read’ approach: the 5′ end of the read was aligned using the flag --trim5 65 and the 3′ end of the read was aligned using the flag --trim3 65. Each such segment must align uniquely with no more than two mismatches (Bowtie flags -v 2 -m 1).

After this step, the algorithm bins the genome into disjoint 50-nucleotide bins (this is a parameter that can be easily updated in scripts we provide by users desiring to evaluate optimal bin size). Note that CIRI [23] circumvents the requirement to select a bin size by using a different dynamic mapping strategy (Burrows-Wheeler Aligner), but does not provide a score-based filtering of novel junctions to reduce false positive results. It then computes all pairs of bins where two separate fragments of the same read have aligned in the same orientation. After this step, the algorithm compiles a list of the offsets among all reads assigned to 50-nucleotide bins based on their offset. If there are at least three reads with unique offsets mapping to the bin pair, the algorithm proceeds as follows: using the offsets of each split pair alignment, the algorithm aligns all reads to each other according to the offset position with respect to the bin. It then calculates a score S, used to screen putative de novo junctions for later consideration, as follows: for each offset with more than one overlapping read, the algorithm counts the most frequent nucleotide (consensus) at each position, and adds $\frac{number\  of\  nucleotide s\  that\  do\  not\  match\  consensus}{total\  number\  of\  sequences\  overlapping\  at\  this\  nucleotide}$ to S. A lower score therefore indicates that more ‘consensus’ has been reached. Under null models that lack of consensus is due to sequencing error alone, the expectation of this score should be approximately:

$$ {\displaystyle \sum \frac{N_ip}{N_i}=}{\displaystyle \sum p} $$

where the sum is over all nucleotides with more than one overlapping sequence, *N*_*i*_ is the number of such sequences at position *i* and *p* is the technical error associated with misreading the wild-type base. For Illumina reads, if *p* = 0.01 and the sum is taken over roughly 100 nucleotides, the expected score is 1. We do not consider the variance of this score in this paper, but only consider de novo junctions where the score is lower than 5. For each pair of bins, the algorithm returns the consensus assembly of all sequences mapping to the (bin A, bin B) pair. A name is assigned to each de novo junction consensus sequence based on a heuristic that if the junction falls within 1 kb of an annotated gene, it is named by that gene and “UNAN” (un-annotated) is assigned otherwise. A de novo index is created from these consensus sequences.

### Processing of de novo reports

All unaligned reads from the annotation-dependent pipeline are realigned to the de novo index using Bowtie2.2.2 and the same alignment criteria described above for annotated junction indices. A read was considered a decoy if the mate aligned to a different chromosome, in the same orientation instead of opposite as expected for paired-end reads, or outside the circle defined by the junction boundary (with a 50-nucleotide buffer due to the fact that mates were assigned to 50-nucleotide bins. Junctions with *p* values > 0.9 using the naïve algorithm detailed above, decoy/circle ratios < 0.1, and bins with breakpoints greater than 200 nucleotides were carried forward in analysis, and bins were subsequently collapsed to unique chromosomal breakpoints.

### Estimating circular RNA read counts in RNase-R+/− data

We applied our algorithm and CIRI to H9 RNase-R+ [SRA:SRR901967] and RNase-R- [SRA:SRR1552724] single-end data and to HeLa RNase-R+ [SRA:SRR1637089] and RNase-R- [SRA:SRR1637090] paired-end data. Our estimate of 70–80 % is with respect to the number of circular junctions supported by at least two reads.

### snoRNA analysis in H9 cells

We obtained a bed file containing genomic locations of snoRNA from the UCSC table browser by selecting Gene Predictions for sno/microRNA gene regions and filtering out type = miRNA. We then downloaded bigWig files from accessions GSM1164885, GSM1480599, and GSM1480600 and used the UCSC utility bigWigAverageOverBed to obtain the average coverage of each snoRNA in the three samples. Of 402 snoRNAs, the 311 that had an average coverage > 0 in at least one of the samples were used in the quantile analysis. For quantification, we used the mean of covered bases as reported by bigWigAverageOverBed.

### Comparison with our previous algorithms

Custom scripts were used to parse data from Table S7 in [3] to determine circular RNA reported by the algorithms in [1] and [3]. The Salzman et al. [1] results are the subset of this table that use RefSeq exon boundaries. The Salzman et al. [3] true positive results are the subset of this table where the sum of the alignment score for read 1 and the alignment score for read 2 is at least −20, while those where the sum is less than −20 are false positives (see [3] for a more thorough discussion of these scores and methods). For our current algorithm, a threshold of 0.9 is applied to the posterior probability to determine true positive and false positive circular RNA candidates.

### Assignment to exterior splicing

Linear junctions where both splice sites are interior to any circular RNA expressed at greater than *k* counts were excluded from the analysis (*k* = 10 for our fetal data and ENCODE data; *k* = 0 for cardiomyocyte data). Less stringent lower bounds were imposed for cardiomyocyte data because sampling depth was much more shallow.

### Calculation of z scores per junction in our fetal data

All circular junctions with at least ten reads were used as “circle boundaries” and all linear reads exterior to these circle boundaries were included in the analysis. Using the calculation described above (*p* value and FDR calculation for circular junction posterior probabilities), this results in an estimated FDR of < 0.001. We repeated this analysis without the minimum read count of 10 (data not shown) and also using linear junctions that shared a splice site with a circular RNA (Additional file 10) and found very similar results. Each sample was normalized for sequencing depth using the median count among all junctions exceeding five counts under a simplifying and common assumption that summed over all genes, both linear and circular, junctions were equally highly expressed at each time point. This method normalizes for junctional read depth and is conceptually similar to RPKM normalization and is achieved with the code below.

Under the assumption that every read aligning to a junction is a random sample from a Poisson distribution (or many other distributions used to model sequencing data), the sufficient statistics (data) for the expression of each junction are the sum of the read counts. Analysis of change of junctional expression over time was computed by z statistics that were functions of these summed counts and defined as:

${x}_i = \frac{r_i}{c_i}$, where *r*_*i*_ are the raw values and *c*_*i*_ are the per-sample normalizing constants above.

Under the null hypothesis of no change over time, {*x*_*i*_} are independent and identically distributed (i.i.d.). Therefore, defining:

*y*_*i*_ = *x*_*i*_ − *μ* for any function *o* with sum ||*o*||_2_^2^ = 1 and ||*o*||_1_ = 0, define the statistic:

$$ z=\frac{<o,y>}{\sqrt{\left|\left|y/c\right|\right|\_1}} $$

Under the null hypothesis that *x*_*i*_ are i.i.d., the numerator of *z*_*i*_ is a linear combination of exchangeable random variables. The mean of the numerator is 0 by construction, so *z* has mean zero as *o* is orthogonal to the unit vector. By independence:

$$ var\left(<o,y>\right)={\left|\left|o\right|\right|}_2^2{\left|\left|\frac{r}{c_s^2}\right|\right|}_1={\left|\left|\frac{x}{c_s}\right|\right|}_1 $$

under the assumption that {*r*_*i*_} are Poisson, the constant *c*_*s*_ has small but nonzero covariance with {*x*_*i*_} and the plug in estimator for *μ* is mean {*x*_*i*_}. We approximate *c*_*s*_ and *x* as being independent. Because ||*o*||_2_^2^ = 1 by construction, under the null hypothesis, *z* is approximately N(0,1). We chose *o*_*i*_ to be proportional to the sample age in weeks of sample *i* so that large values of z can be interpreted as corresponding to consistent and large changes over time.

Figure 4 and Additional files 13 and 17 represent the marginal distributions of z scores per junction for all regular junctions in the exterior category and scatter plots represent each circular junction’s z score versus either the maximum or median of all z scores across all linear junctions in the exterior category. Since in most cases there are many more than one linear junction in the exterior category, the median or maximum z score has a distribution that is the distribution of the median or maximum of normal random variables and therefore has a mean greater than 0. Therefore, comparing the z score for the circular junction to the median or maximum is a conservative comparison for testing that circular RNA is induced compared with linear RNA expression.

### Analysis of relative circular:linear expression in the ENCODE fetal tissue data

All circular junctions with at least ten reads were used as “circle boundaries” and all linear reads exterior to these circle boundaries were included in the analysis. Using the calculation described above (*p* value and FDR calculation for circular junction posterior probabilities), this results in an estimated FDR of < 0.001. After linear reads are assigned to the exterior category for each gene, the maximum count for any exterior linear junction is taken. For each gene, a pair of numbers, *n*_*c*_ (circle count) and *n*_*l*_ (maximum linear count), is used in the following analysis. For each tissue with samples from only two time points, both fetal times, the sample from the earliest time point is considered “early” and the sample from the latest time point is considered “late”. For the heart, where there were samples from three time points, we used the two earliest time points as early and late (see sample key in Additional file 18). For each gene in each of the two samples, a 95 % binomial confidence interval for $\widehat{p} = \frac{n_c}{n_c+{n}_l}$ is calculated. If the confidence intervals in the early and late samples do not overlap, the gene contributes to the count in the barplot in Fig. 5a.

Normalization for plotting was performed as follows: we normalized all counts by dividing by the total number of reads mapped to linear junctions. In analogy with the RPKM, we calculated JRPKM (junctional reads per million mapped) as follows:

we took all reads mapping to a junction and divided by $\frac{total\  reads\  mapped\ to\  linear\  junctions}{\frac{180}{1000}\ *\ {10}^6}$ since by requiring a minimum ten-nucleotide overlap of the junction boundary our effective junction length for 100-nucleotide reads is a window size of 180. Dividing by 180/1000 corrects for this length since if our effective junctional length were 1 kb, this approach would correspond to dividing by 1.

### Outlier analysis methodology

We chose the genes with the 100 most highly expressed circular RNAs (at the level of absolute counts) in the ENCODE fetal dataset and performed the following analysis. First, the fraction of circular reads was calculated where:

$$ \widehat{p} = \frac{n_c}{n_c+{n}_l} $$

*n*_*c*_ being the circular reads and *n*_*l*_ being the exterior linear reads described above. Under the assumption that *n*_*c*_ and *n*_*l*_ are Poisson, conditioned on *n* = *n*_*l*_ + *n*_*c*_, *n*_*c*_ has the binomial distribution with success $\widehat{p}$. We calculated the value:

$$ {z}_n = \frac{\left(\widehat{p}-{\widehat{p}}_{MLE}\right)\sqrt{n}}{\sqrt{\widehat{p}\left(1-\widehat{p}\right)}} $$

estimating $\widehat{p}$ by the maximum likelihood estimator (MLE) if p did not depend on the sample. We further tested whether this value, which should be normally distributed, had any underlying relationship to *n*_*l*_, normalized to the total number of reads. Normalization was performed by dividing the number of reads mapped to each junction by the number of reads mapping to linear junctions. It should be noted that normalization does not impact the relative circular:linear ratio for any gene in a given sample since the circular and linear counts are divided by the same number.

To identify significant outliers and test if there were tissue-consistent patterns in such outliers if they existed, we took the residuals from using the normalized linear reads in a linear model for estimating $\widehat{p}$ (even more conservative than using the MLE described above); this estimate is denoted ${\widehat{p}}_{OLS}$. Using this estimate ${\widehat{p}}_{OLS}$, which like $\widehat{p}$ is a vector, we took the vector of statistics:

$$ {z}_o = \frac{\left(\widehat{p}-{\widehat{p}}_{OLS}\right)\sqrt{n}}{\sqrt{{\widehat{p}}_{OLS}\left(1-{\widehat{p}}_{OLS}\right)}} $$

which should be approximately standard normal under the null hypothesis. To account for the overdispersion in the Poisson, we additionally divided *z*_*o*_ by the empirical median estimated standard deviation of *z*_*o*_, sd(*z*_*o*_), always > 1, and referred the values *z*_*o*_/sd(*z*_*o*_) to the t distribution with *n-2* degrees of freedom where *n* = number of data points. We called outliers by transforming *z*_*o*_/sd(*z*_*o*_) to *p* values and used Benjamini-Hochberg multiple testing correction to control the FDR at level 0.001, identifying samples with circular RNA expression that exceeded what was predicted by the model. Out of 30 genes with more than one outlier, 19 genes were represented by both samples from the same organ (and possibly other outliers also).

### Human ESC cardiac directed differentiation

RUES2 human ESCs were maintained in mouse embryonic fibroblast-conditioned medium. Standard cardiomyocyte directed differentiation using a monolayer platform was performed with a modified protocol based on previous reports [47–49]. The differentiation setup was initiated by plating undifferentiated human ESCs as single cells as described previously [50–52]. The cultures were treated with CHIR-99021 (Cayman chemical,13122) for 24 h before reaching confluence. Cells were induced to differentiate (designated day 0) by replacing the culturing medium with RPMI medium (Invitrogen, 11875–119) containing 100 ng/mL Activin A (R&D Systems, 338-AC-050), 1:60 diluted Matrigel (BD), and insulin-free B27 supplement (Invitrogen, 0050129SA). An RPMI medium change the following day (17 h) included different 5 ng/mL BMP4 (R&D Systems, 314-BP-050), 1 μM CHIR-99021, and insulin-free B27 supplement. On day 3 of differentiation, medium was changed to RPMI medium containing 1 μM XAV-939 (Tocris, 3748) and insulin-free B27 supplement. RPMI containing insulin-free B27 supplement was utilized until differentiation day 7 in which the medium was replaced with RPMI containing a B27 supplement that includes insulin (Invitrogen, 17504044). Subsequent media changes included the insulin-containing supplement. Data are available under accession GSE64417.

### Flow cytometry

Wild-type RUES2 cells were labeled for flow cytometry using an anti-cardiac troponin T antibody (Pierce, MA5-12960) or corresponding isotype control to assess the purity of purified cells. Cells were analyzed using a BD FACSCANTO II with FACSDiva software (BD Biosciences). Instrument settings were adjusted to avoid spectral overlap. Data analysis was performed using FlowJo (Tree Star, Ashland, Oregon). Statistics were: replicate 1, 93 ± 0.3 % cTnT^+^; replicate 2, 92 ± 1.2 % cTnT^+^; replicate 3, 91 ± 0.3 % cTnT^+^.

### Annotation of U2 or U12 flanking motifs

All de novo sequences with score S (described above) < 4 were culled for further analysis. Each junctional read representing the consensus sequence of the de novo junction associated with the bin (see above) was split into all possible sequence fragments of at least 20, modeling each potential breakpoint, and each fragment was separately aligned to the subset of the genome 500 nucleotides upstream and downstream of the 50-nucleotide binned coordinates to reduce complexity of the index, using the Bowtie parameters --all -m 40 -f -v 1 (align fasta files and report all alignments for reads with 40 or fewer alignments with up to one mismatch). The flanking dinucleotides were then computed for each potential junction break point. Candidate junctions where a breakpoint was successfully mapped were carried forward. A Bowtie2 index was built with these sequences and reads were realigned using the same parameters initially used to align to the annotation-dependent junction indices. After realignment of all unaligned reads to the index of de novo assembled sequences where breakpoints could be mapped (as above), we computed the total number of reads mapping to these de novo junctions. Note that in some cases there is ambiguity in exact breakpoint for a junction as the consensus at the 5′ splice site is “ag-GT” (intronic sequence in caps, exonic sequence in lower case). Therefore, if the mapped donor and acceptors ever had a flanking GT-AG (or TATCCT one nucleotide downstream of the donor), the read was called canonical U2 (or U12). To test whether this default assignment biased our results, we repeated the di-nucleotide enrichment analysis for all 4^4^ possible pairs of “donors” and “acceptors”. All of these decoy flanking dinucleotides were significantly less, if at all, enriched than “GT/AG”. Although several U12 flanking dinucleotides were identified, as with linear RNA, these account for a small minority of circular RNA splicing.

### Data availability

RNA-Seq data and processed reports for our fetal samples, as well as processed reports for the ENCODE fetal samples analyzed, are available under accession GSE64283. RNA-Seq data and processed reports for induced cardiomyocytes are available under accession GSE64417.

### Code availability

The algorithm for the quantification of splicing events and assigning a confidence to each junction is written in a set of custom bash, Python, Perl, and R scripts. Running the entire pipeline requires invoking two bash commands, described in a README file available with the code. Code is provided to execute the analysis pipeline on a single Linux machine or on a cluster using SLURM. The code is available at [53].

## Additional files

Additional file 1:
**Comparison to CIRI and find_circ.** Data used to generate Fig. 3a–c comparing our sensitivity to the sensitivity of de novo algorithms. Note that due to numerical precision, *p* values and posterior probabilities > 0.999999999 are reported as 1 and values < 1.0 * 10^−45^ are reported as 0. Additional file 2:
**snoRNA in H9 cells.** Mean coverage for covered bases as reported by UCSC bigWigAverageOverBed utility. Additional file 3:
**Comparison to CIRI on H9 cells.** Overlap in results from our GLM algorithm on single-end data from H9 poly(A)+, poly(A)-, and RNase-R+ samples compared with overlap on these samples reported by CIRI. Additional file 4:
**Comparison to our previous algorithms.** Our 2012 (PLosOne) algorithm [1] identified circularRNA candidates without applying a statistical filter. Our 2013 (PLosGenetics) algorithm [3] introduced an FDR that reduced false positive (FP) results. The GLM method presented here increases sensitivity while also increasing specificity. Comparisons of the three algorithms on ENCODE poly(A)- data are shown, with circles flagged as FPs by the algorithm shown in shaded regions (FDR > 0.025 for PLosGenetics or posterior probability < 0.9 for GLM) and those reported as circular RNA candidates are shown in non-shaded regions. For GLM results, the total number of circular RNAs is shown, with the count of those circles identified by the de novo portion of the algorithm called out in parentheses. a In HeLa poly(A)- cells (Rep1) 6002 circular RNA candidates were identified in PLosOne 2012. Our PLosGenetics 2013 algorithm identified 6831 circles, most also identified by the previous algorithm, but used the FDR to flag 3668 of these candidates as FPs. The GLM method has increased sensitivity and identifies 4761 circular RNAs as likely true positives and 1656 circular RNAs with aligned reads were flagged as FPs. b In H1 poly(A)- cells (Rep1) 8622 circular RNA candidates were identified in by our PLosOne 2012 algorithm. Our PlosGenetics 2013 algorithm identified 6831 circles, most also identified by the previous algorithm, but used the FDR to flag 3668 of these candidates as FPs. The GLM method has increased sensitivity and identifies 4761 circular RNAs as likely true positives and 1656 circular RNAs with aligned reads were flagged as FPs. Additional file 5:
**Comparison to Zhang algorithm on H9 cells.** Data used to generate Fig. 3d comparing our sensitivity to the sensitivity of Zhang et al. 2014 [22] on H9 RNase-R-treated cells. Table S1 Circles identified by both our annotation-dependent algorithm and the Zhang algorithm. Table S2 Circles identified only by the Zhang algorithm. Table S3 Circles identified only by our annotation-dependent algorithm. Table S4 Circles that were identified by Zhang et al. and missed by our annotation-dependent algorithm, but subsequently detected using our de novo algorithm. Note that due to numerical precision, *p* values > 0.999999999 are reported as 1. Additional file 6:
**Number of circles by exon size in H9 cells.** Data used in plot for Fig. 3d, instead showing number of distinct circles by length. Additional file 7:
**Circular and linear splicing identified in mouse R1 ESCs.** Linear and circular RNA splices identified in mouse ESCs, including circular Polr2a. Report generated from analysis using the naïve method because this is single-end RNA-Seq. Note that due to numerical precision, *p* values > 0.999999999 are reported as 1. Additional file 8:
**Additional circular RNAs identified by the de novo pipeline.** Exonic sequences from genome annotation are given in *uppercase*, and intronic sequences in *lowercase* with splice-signal dinucleotides highlighted in *red*. Definitive U12-type introns are indicated by “*U12*” in *green*. a TCEA3 backsplices from a U12 splice donor to a cryptic acceptor slightly upstream of the annotated exon; the dinucleotides used are AT-AC. b MORC3 backsplices from a U12 splice donor to several different locations close to, but not including, the annotated exon boundary (even though the annotated splice acceptor is also U12 type). c RMST circular isoform was identified only by the de novo pipeline, as it involves exons not present in the RefSeq gene model. Three UCSC gene models are shown in the Genome Browser snapshot. Below that is a hybrid gene model with 13 exons, combining exons from different UCSC gene models. The RefSeq gene model consists of exons 1–9 (same as the first UCSC gene model). The observed circle is a backsplice between exons 11 and 3. Additional file 9:
**Primers and sequencing results.**
Additional file 10:
**Gel analysis of RT-PCR of circular isoforms.** a Agarose gels of RT-PCR products (after 40 cycles) of U12-type circular isoforms for TCEA3, RANBP17, MORC3, and ATXN10, which were TOPO-cloned and Sanger-sequenced (Additional file 8), which demonstrated multiple circular isoforms for most of these genes (in particular, explaining the multiple bands seen for ATXN10). The outward-facing primers were located in the same exon, so products are nearly the full size of the circle. The asterisk marks a primer-dimer band in the MORC3 lane. b Agarose gel of bands after RT-qPCR (45 cycles) for circular (*cir*) and linear (*lin*) isoforms of genes regulated in fetal development. The NCX1 circular isoform product was directly Sanger-sequenced and also TOPO-cloned and sequenced; note that it appears as a single band, since the variant isoform is only 3 bp shorter than the main circular isoform. Additional file 11:
**RNase-R resistance of circular isoforms.** Circular isoforms show resistance to the exoribonuclease RNase-R, compared with linear isoforms, in the fibroblast cell line BJ. Values plotted are ΔΔCt = ΔCt(circle) – ΔCt(linear), where ΔCt = Ct(mock-treated) – Ct(RNase-R-treated); error bars are standard error of the mean of technical replicates. The absolute Ct values shown are for mock-treated RNA. Additional file 12:
**Our fetal data sample key.** Sample_ID, index sequence, and gestational age for the fetal data we generated. Additional file 13:
**Z score plots for each of our fetal samples.** For our fetal tissue data, z score plots per organ, leaving out earliest time point to be conservative. Clockwise from top left, histogram of linear z scores, circular z scores, plots of circular z score versus median and maximum linear z score per gene. Linear junctions were used if a splice site was excluded from any splice site used in circular RNA having at least ten counts. Points with most positive z score are labeled for visualization. Additional file 14:
**Circular induction in fetal intestine and stomach.** RT-qPCR confirms greater induction of circular RNA in several organs; intestine and stomach are shown here (heart and lung in Fig. 3b). Plotted values are ΔΔCt = ΔCt(age 20 weeks) – ΔCt(age 10 weeks), where ΔCt = Ct(ACTB) – Ct(target). Error bars are standard error of the mean of technical replicates. Positive ΔΔCt indicates increased expression later in development, and is log2 scale. Additional file 15:
**qPCR validation of sequencing-based quantification.**
Additional file 16:
**correlation of qPCR and sequencing-based quantification.**
Additional file 17:
**Z score plots per tissue for our fetal samples.** For our fetal tissue data, z score plots per organ, leaving out earliest time point to be conservative. Clockwise from top left, histogram of linear z scores, circular z scores, plots of circular z score versus median and maximum linear z score per gene. Linear junctions were used if a splice site was shared with any splice site used in circular RNA having at least ten counts. Points with most positive z score are labeled for visualization. Additional file 18:
**ENCODE fetal data sample key.** Tissue and age in weeks. Additional file 19:
**Outlier list by tissue.** Outlier list by tissue; “early” and “late” correspond to earliest and second earliest time points per tissue. Additional file 20:
**Cardiomyocyte processed reports.** Junctions with a posterior probability exceeding *p* = 0.9 and at least one read are reported for each of three biological replicates of the cardiomyocyte time course. Note that due to numerical precision, posterior probabilities > 0.999999999 are reported as 1. Additional file 21:
**Z score plots for cardiomyocytes.** Clockwise from top left, histogram of linear z scores, circular z scores, plots of circular z score versus median and maximum linear z score per gene. Linear junctions were used if a splice site was shared with any circular RNA having at least one count. Points with most positive z score are labeled for visualization. Additional file 22:
**Comparison of de novo and annotated circular RNA counts.** Many de novo junctions have high expression, comparable to circular RNA expression from RNA spliced at canonical boundaries. We plotted empirical cumulative distributions of total circular counts per gene from all annotated junctions and separately for all de novo junctions after reports had been screened as described in the methods; total expression estimates were collapsed across samples and reported at the gene level. *ECDF* empirical cumulative distribution function. Additional file 23:
**ENCODE circular junctions detected at canonical U12 splice sites.** Note that due to numerical precision, posterior probabilities > 0.999999999 are reported as 1. Additional file 24:
**ENCODE linear junctions detected at canonical U12 splice sites.** Note that due to numerical precision, posterior probabilities > 0.999999999 are reported as 1. Additional file 25:
**Cumulative distribution of predictors used in GLM.** Bowtie2 alignment score, mapping quality, and the amount of junction overlap are distributed differently in the two categories of reads used to fit the model: 1) that map to canonical linear isoforms (real alignments); 2) that are likely artifacts because their relative alignment orientations are inconsistent with coming from a linear or circular RNA (decoy alignments).

## Abbreviations

ESC: embryonic stem cell
FDR: false discovery rate
GLM: generalized linear model
i.i.d.: independent and identically distributed
PCR: polymerase chain reaction
qPCR: quantitative PCR
RT: reverse transcription
snoRNA: small nucleolar RNA
SRA: Sequence Read Archive

## References

[1] Salzman J, Gawad C, Wang PL, Lacayo N, Brown PO (2012). Circular RNAs are the predominant transcript isoform from hundreds of human genes in diverse cell types. PLoS One.

[2] Memczak S, Jens M, Elefsinioti A, Torti F, Krueger J, Rybak A (2013). Circular RNAs are a large class of animal RNAs with regulatory potency. Nature.

[3] Salzman J, Chen RE, Wang PL, Olsen M, Brown PO (2013). Cell-type specific regulation of circular RNA expression. PLoS Genet.

[4] Wang PL, Bao Y, Yee MC, Barrett SP, Hogan GJ, Olsen MN (2014). Circular RNA Is Expressed across the eukaryotic Tree of Life. PLoS One.

[5] Jeck WR, Sorrentino JA, Wang K, Slevin MK, Burd CE, Liu J (2013). Circular RNAs are abundant, conserved, and associated with ALU repeats. RNA.

[6] Guo JU, Agarwal V, Guo H, Bartel DP (2014). Expanded identification and characterization of mammalian circular RNAs. Genome Biol.

[7] Hansen TB, Jensen TI, Clausen BH, Bramsen JB, Finsen B, Damgaard CK (2013). Natural RNA circles function as efficient microRNA sponges. Nature.

[8] Westholm JM, Miura P, Olson S, Shenker S, Joseph B, Sanfilippo P (2014). Genome-wide analysis of Drosophila circular RNAs reveals their structural and sequence properties and age-dependent neural accumulation. Cell Rep.

[9] Liang D, Wilusz JE (2014). Short intronic repeat sequences facilitate circular RNA production. Genes Dev.

[10] Capel B, Swain A, Nicolis S, Hacker A, Walter M, Koopman P (1993). Circular transcripts of the testis-determining gene Sry in adult mouse testis. Cell.

[11] Ng SY, Bogu GK, Soh BS, Stanton LW (2013). The long noncoding RNA RMST interacts with SOX2 to regulate neurogenesis. Mol Cell.

[12] Cho CH, Kim SS, Jeong MJ, Lee CO, Shin HS (2000). The Na + −Ca2+ exchanger is essential for embryonic heart development in mice. Mol Cell.

[13] Li XF, Lytton J (1999). A circularized sodium-calcium exchanger exon 2 transcript. J Biol Chem.

[14] Salzman J, Datta SaN D (2014). Statistical analysis of next generation sequencing data. Frontiers in probability and the statistical sciences.

[15] Shen S, Lin L, Cai JJ, Jiang P, Kenkel EJ, Stroik MR (2011). Widespread establishment and regulatory impact of Alu exons in human genes. Proc Natl Acad Sci U S A.

[16] Yu CY, Liu HJ, Hung LY, Kuo HC, Chuang TJ (2014). Is an observed non-co-linear RNA product spliced in trans, in cis or just in vitro?. Nucleic Acids Res.

[17] van Gurp TP, McIntyre LM, Verhoeven KJ (2013). Consistent errors in first strand cDNA due to random hexamer mispriming. PLoS One.

[18] Lahens NF, Kavakli IH, Zhang R, Hayer K, Black MB, Dueck H (2014). IVT-seq reveals extreme bias in RNA sequencing. Genome Biol.

[19] McCullagh P, Nelder JA (1989). Generalized linear models.

[20] Starke S, Jost I, Rossbach O, Schneider T, Schreiner S, Hung LH (2015). Exon circularization requires canonical splice signals. Cell Rep.

[21] Jeck WR, Sharpless NE (2014). Detecting and characterizing circular RNAs. Nat Biotechnol.

[22] Zhang XO, Wang HB, Zhang Y, Lu X, Chen LL, Yang L (2014). Complementary sequence-mediated exon circularization. Cell.

[23] Gao Y, Wang J, Zhao F (2015). CIRI: an efficient and unbiased algorithm for de novo circular RNA identification. Genome Biol.

[24] Roberts A, Pachter L (2013). Streaming fragment assignment for real-time analysis of sequencing experiments. Nat Methods.

[25] Ivanov A, Memczak S, Wyler E, Torti F, Porath HT, Orejuela MR (2015). Analysis of intron sequences reveals hallmarks of circular RNA biogenesis in animals. Cell Rep.

[26] Hoffmann S, Otto C, Doose G, Tanzer A, Langenberger D, Christ S (2014). A multi-split mapping algorithm for circular RNA, splicing, trans-splicing and fusion detection. Genome Biol.

[27] Turunen JJ, Niemela EH, Verma B, Frilander MJ (2013). The significant other: splicing by the minor spliceosome. Wiley Interdiscip Rev RNA.

[28] Gehman LT, Stoilov P, Maguire J, Damianov A, Lin CH, Shiue L (2011). The splicing regulator Rbfox1 (A2BP1) controls neuronal excitation in the mammalian brain. Nat Genet.

[29] Kalsotra A, Xiao X, Ward AJ, Castle JC, Johnson JM, Burge CB (2008). A postnatal switch of CELF and MBNL proteins reprograms alternative splicing in the developing heart. Proc Natl Acad Sci U S A.

[30] Kalsotra A, Wang K, Li PF, Cooper TA (2010). MicroRNAs coordinate an alternative splicing network during mouse postnatal heart development. Genes Dev.

[31] Giudice J, Xia Z, Wang ET, Scavuzzo MA, Ward AJ, Kalsotra A (2014). Alternative splicing regulates vesicular trafficking genes in cardiomyocytes during postnatal heart development. Nat Commun.

[32] Almeida AD, Wise HM, Hindley CJ, Slevin MK, Hartley RS, Philpott A (2010). The F-box protein Cdc4/Fbxw7 is a novel regulator of neural crest development in Xenopus laevis. Neural Dev.

[33] Matsumoto A, Onoyama I, Sunabori T, Kageyama R, Okano H, Nakayama KI (2011). Fbxw7-dependent degradation of Notch is required for control of “stemness” and neuronal-glial differentiation in neural stem cells. J Biol Chem.

[34] Jordan MC, Henderson SA, Han T, Fishbein MC, Philipson KD, Roos KP (2010). Myocardial function with reduced expression of the sodium-calcium exchanger. J Card Fail.

[35] Bradley RK, Merkin J, Lambert NJ, Burge CB (2012). Alternative splicing of RNA triplets is often regulated and accelerates proteome evolution. PLoS Biol.

[36] Otake LR, Scamborova P, Hashimoto C, Steitz JA (2002). The divergent U12-type spliceosome is required for pre-mRNA splicing and is essential for development in Drosophila. Mol Cell.

[37] Markmiller S, Cloonan N, Lardelli RM, Doggett K, Keightley MC, Boglev Y (2014). Minor class splicing shapes the zebrafish transcriptome during development. Proc Natl Acad Sci U S A.

[38] Jafarifar F, Dietrich RC, Hiznay JM, Padgett RA (2014). Biochemical defects in minor spliceosome function in the developmental disorder MOPD I. RNA.

[39] White MC, Gao R, Xu W, Mandal SM, Lim JG, Hazra TK (2010). Inactivation of hnRNP K by expanded intronic AUUCU repeat induces apoptosis via translocation of PKCdelta to mitochondria in spinocerebellar ataxia 10. PLoS Genet.

[40] Ashwal-Fluss R, Meyer M, Pamudurti NR, Ivanov A, Bartok O, Hanan M (2014). circRNA biogenesis competes with pre-mRNA splicing. Mol Cell.

[41] CHSL Long RNA-seq Downloadable Files. http://genome.ucsc.edu/cgi-bin/hgFileUi?db=hg19&g=wgEncodeCshlLongRnaSeq.

[42] ENCODE Experiment Search. https://www.encodeproject.org/search/?type=experiment.

[43] Trim Galore!. http://www.bioinformatics.babraham.ac.uk/projects/trim_galore/.

[44] cutAdapt. https://code.google.com/p/cutadapt/.

[45] Illumina iGenomes. http://support.illumina.com/sequencing/sequencing_software/igenome.html.

[46] Bowtie2. http://bowtie-bio.sourceforge.net/bowtie2/index.shtml.

[47] Laflamme MA, Chen KY, Naumova AV, Muskheli V, Fugate JA, Dupras SK (2007). Cardiomyocytes derived from human embryonic stem cells in pro-survival factors enhance function of infarcted rat hearts. Nat Biotechnol.

[48] Paige SL, Osugi T, Afanasiev OK, Pabon L, Reinecke H, Murry CE (2010). Endogenous Wnt/beta-catenin signaling is required for cardiac differentiation in human embryonic stem cells. PLoS One.

[49] Lian X, Hsiao C, Wilson G, Zhu K, Hazeltine LB, Azarin SM (2012). Robust cardiomyocyte differentiation from human pluripotent stem cells via temporal modulation of canonical Wnt signaling. Proc Natl Acad Sci U S A.

[50] Gantz JA, Palpant NJ, Welikson RE, Hauschka SD, Murry CE, Laflamme MA (2012). Targeted genomic integration of a selectable floxed dual fluorescence reporter in human embryonic stem cells. PLoS One.

[51] Palpant NJ, Pabon L, Rabinowitz JS, Hadland BK, Stoick-Cooper CL, Paige SL (2013). Transmembrane protein 88: a Wnt regulatory protein that specifies cardiomyocyte development. Development.

[52] Chong JJ, Yang X, Don CW, Minami E, Liu YW, Weyers JJ (2014). Human embryonic-stem-cell-derived cardiomyocytes regenerate non-human primate hearts. Nature.

[53] Source code. https://github.com/lindaszabo/KNIFE.

