# Erratum to: Statistically based splicing detection reveals neural enrichment and tissue-specific induction of circular RNA during human fetal development

**DOI:** 10.1186/s13059-016-1123-9

**Published:** 2016-12-19

**Authors:** Linda Szabo, Robert Morey, Nathan J. Palpant, Peter L. Wang, Nastaran Afari, Chuan Jiang, Mana M. Parast, Charles E. Murry, Louise C. Laurent, Julia Salzman

**Affiliations:** 1 Stanford Department of Biochemistry and Stanford Cancer Institute, Stanford, CA USA; 2 UC San Diego Department of Reproductive Medicine, San Diego, CA USA; 3 Center for Cardiovascular Biology, Institute for Stem Cell and Regenerative Medicine, Departments of Pathology, Bioengineering and Medicine/Cardiology, University of Washington, Seattle, WA 98109 USA; 4 UC San Diego Department of Pathology, San Diego, CA USA

## Erratum

In this version of this article that was originally published [1] the authors had analysed two HeLa samples, SRR1637089 and SRR1637090, in Fig. 3 of the original publication. The authors had respectively analysed the samples as RNaseR+ and Ribominus, due to their incorrect annotations in a public database, but they were both Ribominus samples. The authors have now analysed appropriate positive and negative controls using their method, KNIFE, and find_circ. The results are presented in an amended version of Fig. 3c, please see updated version below. Furthermore, the authors have now provided a list of accession codes for the ENCODE data they analysed, please see the Table 1 below. Please note this was not part of the original article.

**Fig. 3:**
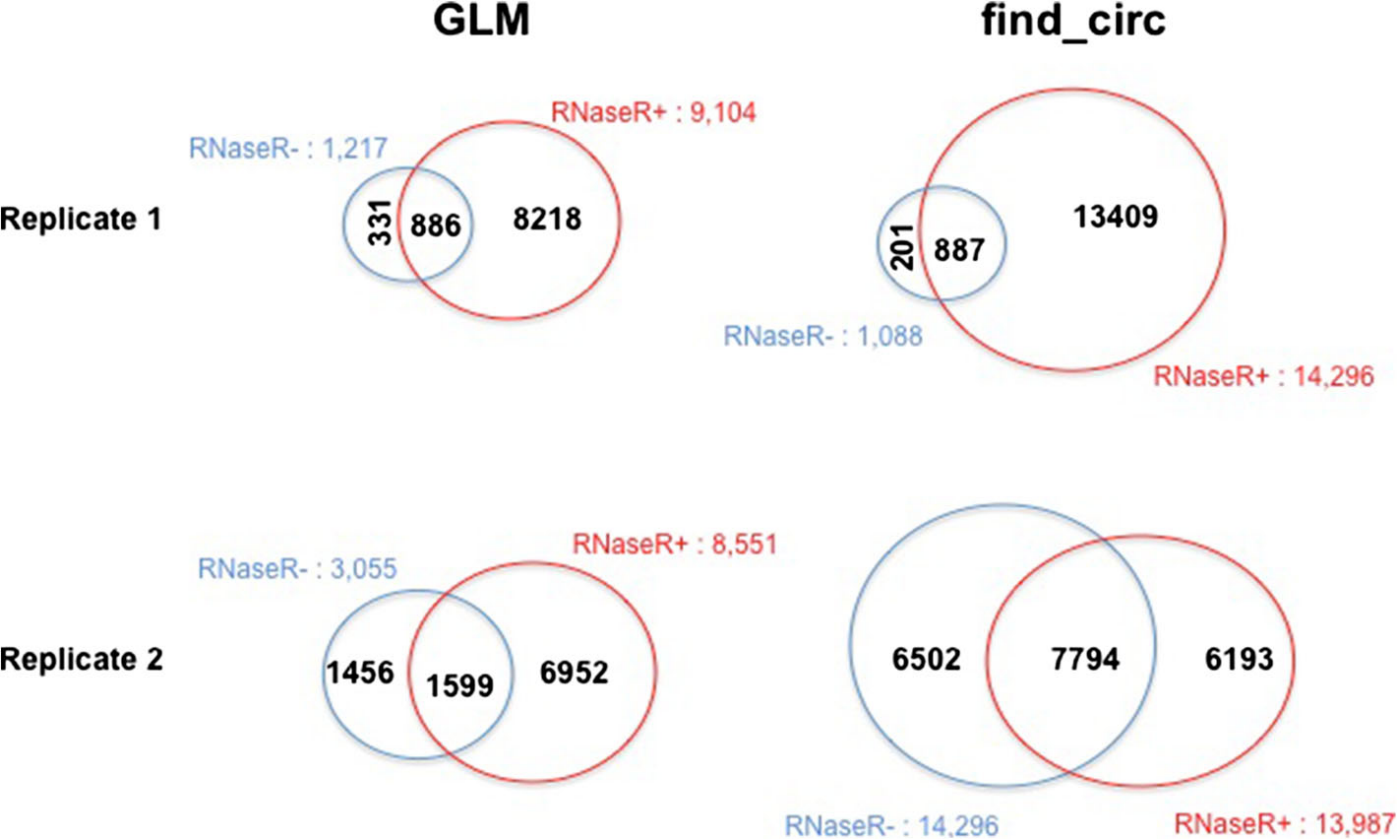
Statistical algorithm improves the sensitivity of circular RNA detection. **a**, **b** Circular RNA detected by both algorithms are divided into false positives (*FP*; flagged as false positives due to low posterior probability) or true positives (*TP*; our posterior probability ≥ 0.9). **a** Number of circular RNAs detected by our GLM or CIRI in ENCODE BJ poly(A)+/− data and HeLa RNase-R+/− data generated by Gao et al. [23]. CIRI results are based on all default parameters except the -E flag set to exclude false positives resulting from identical colinear exons. **b** Number of circular RNAs detected by our GLM or find_circ in ENCODE BJ poly(A)+/− data and HeLa RNase-R- data generated by Gao et al. [23]. **c** Circular RNAs detected in HeLa RNase-R+ and Ribo- data generated by Gao et al. [23] and poly(A)+, and poly(A)- data generated by ENCODE. Number of circular RNAs detected by our GLM method (one or more reads, posterior probability ≥ 0.9) compared with CIRI (default parameters except -E). For GLM results, the first number is the total number of circles and the number of those which were detected by the de novo portion of the algorithm are listed in parentheses. **d** Venn diagram comparing the number of putative circular RNAs identified by our annotation-dependent algorithm in Rnase-R-treated H9 cells and the results published by Zhang et al. [22]. *Green circles* and *red circles* show circular RNA identified by our algorithm with high and low confidence, respectively; the *blue circle* shows those identified by Zhang et al. **e** Total junctional reads for circles comprised of a single exon (posterior probability ≥ 0.9, read count > 1) shown by size for same data as in panel (d). Median exon length is shown in *red*. The x-axis is truncated at 2000 excluding 31 long exons, all but one with total read counts < 50]

**Table 1:** ENCODE accession codes

Source	Type	ACCESSION
A549	cell line, polyA+	GSM758564
AGO4450	cell line, polyA+	GSM758561
BJ	cell line, polyA+	GSM758562
GM12878	cell line, polyA+	GSM758559
H1	cell line, polyA+	GSM758566
HMEC	cell line, polyA+	GSM758571
HeLa	cell line, polyA+	GSM765402
HepG2	cell line, polyA+	GSM758575
HSSM	cell line, polyA+	GSM758578
HUVEC	cell line, polyA+	GSM758563
IMR90	cell line, polyA+	GSM981249
K562	cell line, polyA+	GSM765405
MCF7	cell line, polyA+	GSM765388
NHEK	cell line, polyA+	GSM765401
NHLF	cell line, polyA+	GSM765394
SKNSHRA	cell line, polyA+	GSM765395
A549	cell line, polyA-	GSM767854
AGO4450	cell line, polyA-	GSM765396
BJ	cell line, polyA-	GSM767855
GM12878	cell line, polyA-	GSM758572
H1	cell line, polyA-	GSM758573
HMEC	cell line, polyA-	GSM765397
HeLa	cell line, polyA-	GSM767847
HepG2	cell line, polyA-	GSM758567
HSSM	cell line, polyA-	GSM765391
HUVEC	cell line, polyA-	GSM767856
K562	cell line, polyA-	GSM758577
MCF7	cell line, polyA-	GSM767851
NHEK	cell line, polyA-	GSM765398
NHLF	cell line, polyA-	GSM765389
SKNSHRA	cell line, polyA-	GSM767845
camera-type eye	tissue	ENCSR000AFO
cerebellum	tissue	ENCSR000AEW
diencephalon	tissue	ENCSR000AEX
frontal cortex	tissue	ENCSR000AEY
heart	tissue	ENCSR000AEZ
heart	tissue	ENCSR000AHH
liver	tissue	ENCSR000AEU
liver	tissue	ENCSR000AFB
lung	tissue	ENCSR000AFC
metanephros	tissue	ENCSR000AFA
mononuclear cell	tissue	ENCSR000CUT
occipital lobe	tissue	ENCSR000AFD
parietal lobe	tissue	ENCSR000AFE
skeletal muscle	tissue	ENCSR000AFF
skin of body	tissue	ENCSR000AFG
spinal cord	tissue	ENCSR000AFH
stomach	tissue	ENCSR000AFI
temporal lobe	tissue	ENCSR000AFJ
thyroid gland	tissue	ENCSR000AFK
tongue	tissue	ENCSR000AFL
umbilical cord	tissue	ENCSR000AFM
urinary bladder	tissue	ENCSR000AEV
uterus	tissue	ENCSR000AFN

Source (cell line name or tissue type), Type of sample (tissue, polyA+ cell line, or polyA- cell line), and Accession code for all ENCODE data analyzed.]
