# Supplementary figures and images for: Statistically based splicing detection reveals neural enrichment and tissue-specific induction of circular RNA during human fetal development
Source: Genome Biol. 2015 Jun 16;16(1):126. doi: 10.1186/s13059-015-0690-5 (PMC4506483; doi:10.1186/s13059-015-0690-5)

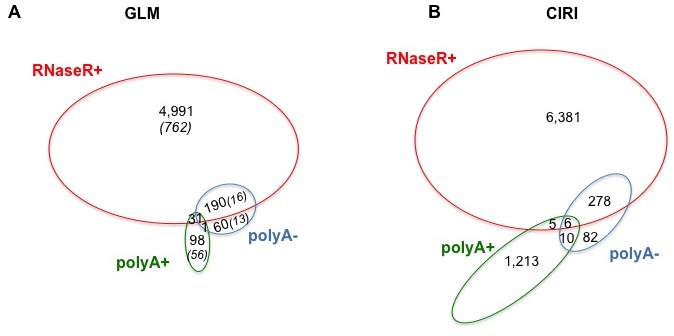

Supplement: Additional file 3: — Comparison to CIRI on H9 cells. Overlap in results from our GLM algorithm on single-end data from H9 poly(A)+, poly(A)-, and RNase-R+ samples compared with overlap on these samples reported by CIRI. [file 13059_2015_690_MOESM3_ESM.jpg]

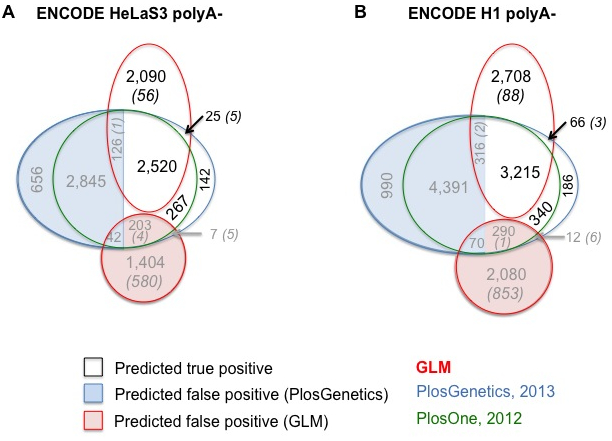

Supplement: Additional file 4: — Comparison to our previous algorithms. Our 2012 (PLosOne) algorithm [1] identified circularRNA candidates without applying a statistical filter. Our 2013 (PLosGenetics) algorithm [3] introduced an FDR that reduced false positive (FP) results. The GLM method presented here increases sensitivity while also increasing specificity. Comparisons of the three algorithms on ENCODE poly(A)- data are shown, with circles flagged as FPs by the algorithm shown in shaded regions (FDR > 0.025 for PLosGenetics or posterior probability < 0.9 for GLM) and those reported as circular RNA candidates are shown in non-shaded regions. For GLM results, the total number of circular RNAs is shown, with the count of those circles identified by the de novo portion of the algorithm called out in parentheses. a In HeLa poly(A)- cells (Rep1) 6002 circular RNA candidates were identified in PLosOne 2012. Our PLosGenetics 2013 algorithm identified 6831 circles, most also identified by the previous algorithm, but used the FDR to flag 3668 of these candidates as FPs. The GLM method has increased sensitivity and identifies 4761 circular RNAs as likely true positives and 1656 circular RNAs with aligned reads were flagged as FPs. b In H1 poly(A)- cells (Rep1) 8622 circular RNA candidates were identified in by our PLosOne 2012 algorithm. Our PlosGenetics 2013 algorithm identified 6831 circles, most also identified by the previous algorithm, but used the FDR to flag 3668 of these candidates as FPs. The GLM method has increased sensitivity and identifies 4761 circular RNAs as likely true positives and 1656 circular RNAs with aligned reads were flagged as FPs. [file 13059_2015_690_MOESM4_ESM.jpg]

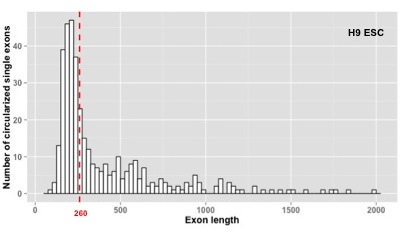

Supplement: Additional file 6: — Number of circles by exon size in H9 cells. Data used in plot for Fig. 3d, instead showing number of distinct circles by length. [file 13059_2015_690_MOESM6_ESM.jpg]

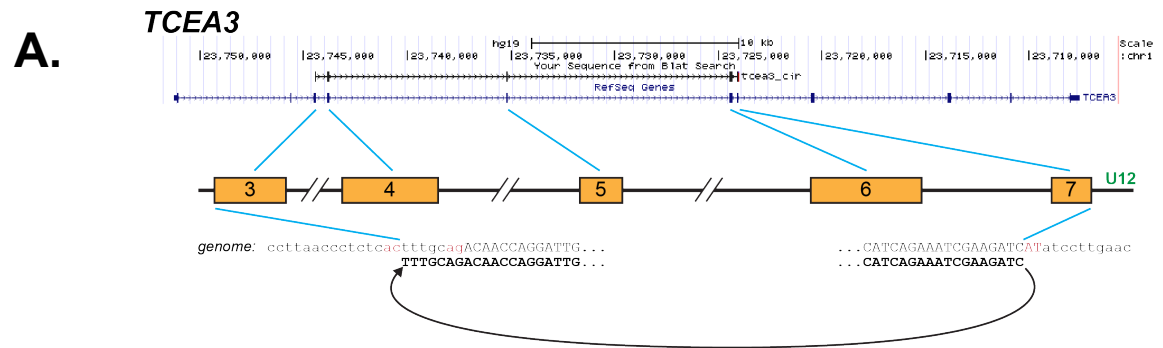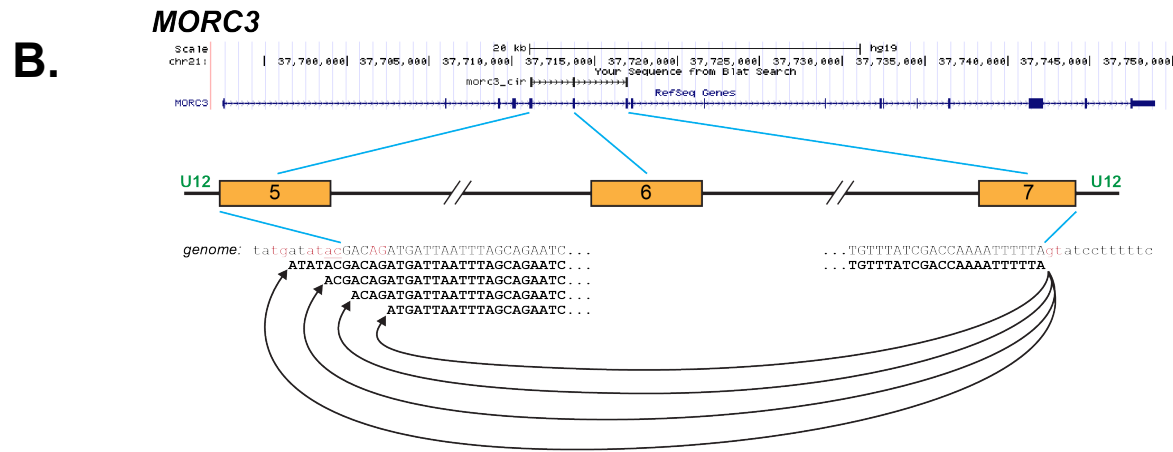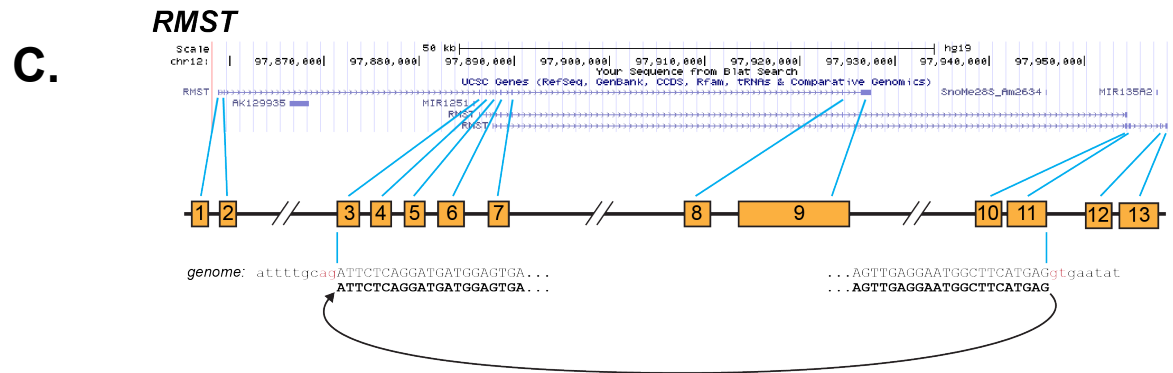

Supplement: Additional file 8: — Additional circular RNAs identified by the de novo pipeline. Exonic sequences from genome annotation are given in uppercase, and intronic sequences in lowercase with splice-signal dinucleotides highlighted in red. Definitive U12-type introns are indicated by “U12” in green. a TCEA3 backsplices from a U12 splice donor to a cryptic acceptor slightly upstream of the annotated exon; the dinucleotides used are AT-AC. b MORC3 backsplices from a U12 splice donor to several different locations close to, but not including, the annotated exon boundary (even though the annotated splice acceptor is also U12 type). c RMST circular isoform was identified only by the de novo pipeline, as it involves exons not present in the RefSeq gene model. Three UCSC gene models are shown in the Genome Browser snapshot. Below that is a hybrid gene model with 13 exons, combining exons from different UCSC gene models. The RefSeq gene model consists of exons 1–9 (same as the first UCSC gene model). The observed circle is a backsplice between exons 11 and 3. [file 13059_2015_690_MOESM8_ESM.pdf]

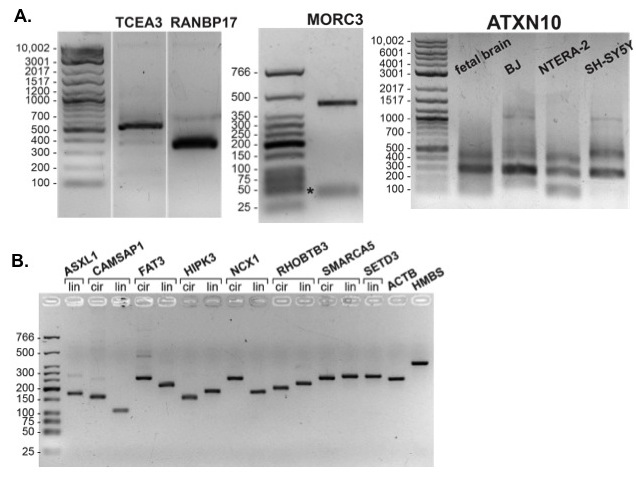

Supplement: Additional file 10: — Gel analysis of RT-PCR of circular isoforms. a Agarose gels of RT-PCR products (after 40 cycles) of U12-type circular isoforms for TCEA3, RANBP17, MORC3, and ATXN10, which were TOPO-cloned and Sanger-sequenced (Additional file 8), which demonstrated multiple circular isoforms for most of these genes (in particular, explaining the multiple bands seen for ATXN10). The outward-facing primers were located in the same exon, so products are nearly the full size of the circle. The asterisk marks a primer-dimer band in the MORC3 lane. b Agarose gel of bands after RT-qPCR (45 cycles) for circular (cir) and linear (lin) isoforms of genes regulated in fetal development. The NCX1 circular isoform product was directly Sanger-sequenced and also TOPO-cloned and sequenced; note that it appears as a single band, since the variant isoform is only 3 bp shorter than the main circular isoform. [file 13059_2015_690_MOESM10_ESM.jpg]

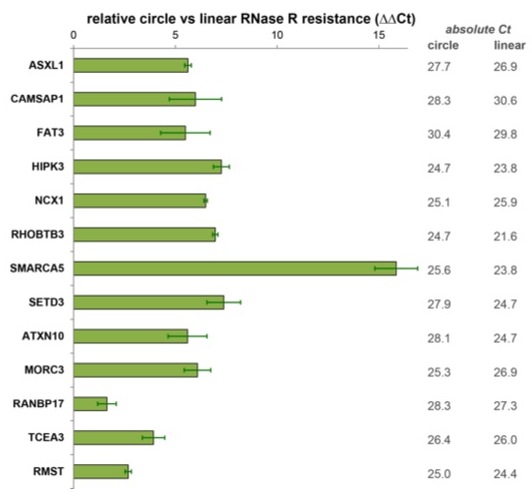

Supplement: Additional file 11: — RNase-R resistance of circular isoforms. Circular isoforms show resistance to the exoribonuclease RNase-R, compared with linear isoforms, in the fibroblast cell line BJ. Values plotted are ΔΔCt = ΔCt(circle) – ΔCt(linear), where ΔCt = Ct(mock-treated) – Ct(RNase-R-treated); error bars are standard error of the mean of technical replicates. The absolute Ct values shown are for mock-treated RNA. [file 13059_2015_690_MOESM11_ESM.jpg]

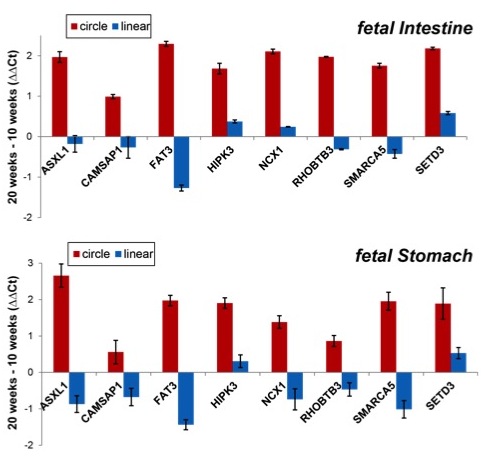

Supplement: Additional file 14: — Circular induction in fetal intestine and stomach. RT-qPCR confirms greater induction of circular RNA in several organs; intestine and stomach are shown here (heart and lung in Fig. 3b). Plotted values are ΔΔCt = ΔCt(age 20 weeks) – ΔCt(age 10 weeks), where ΔCt = Ct(ACTB) – Ct(target). Error bars are standard error of the mean of technical replicates. Positive ΔΔCt indicates increased expression later in development, and is log2 scale. [file 13059_2015_690_MOESM14_ESM.jpg]

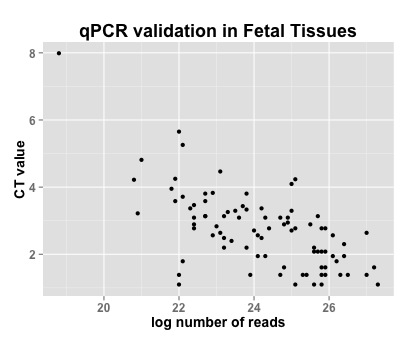

Supplement: Additional file 16: — correlation of qPCR and sequencing-based quantification. [file 13059_2015_690_MOESM16_ESM.jpeg]

**ECDF of log annotated circle counts per gene**

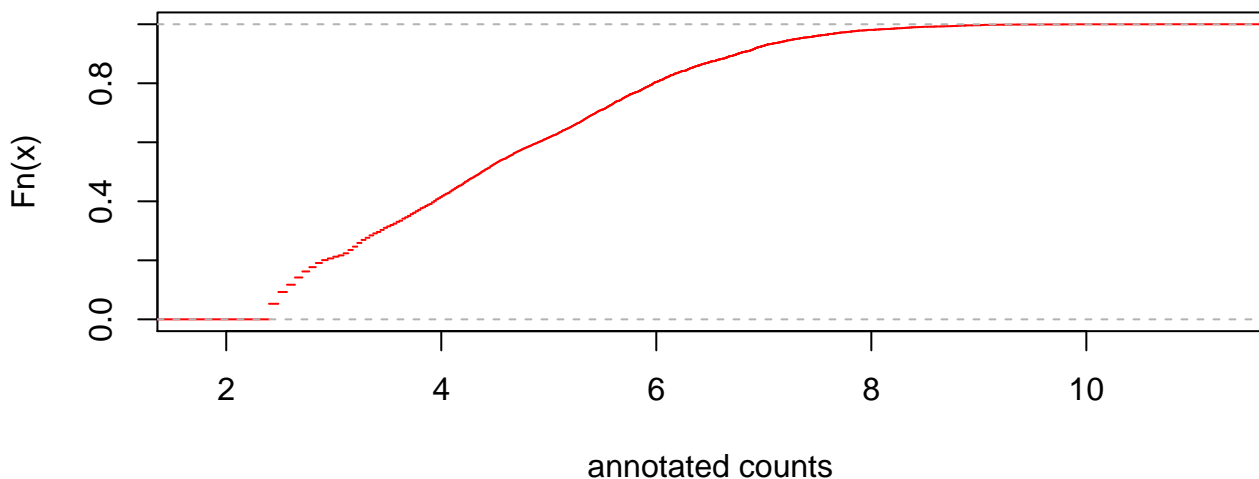

**ECDF of log denovo circle counts per gene**

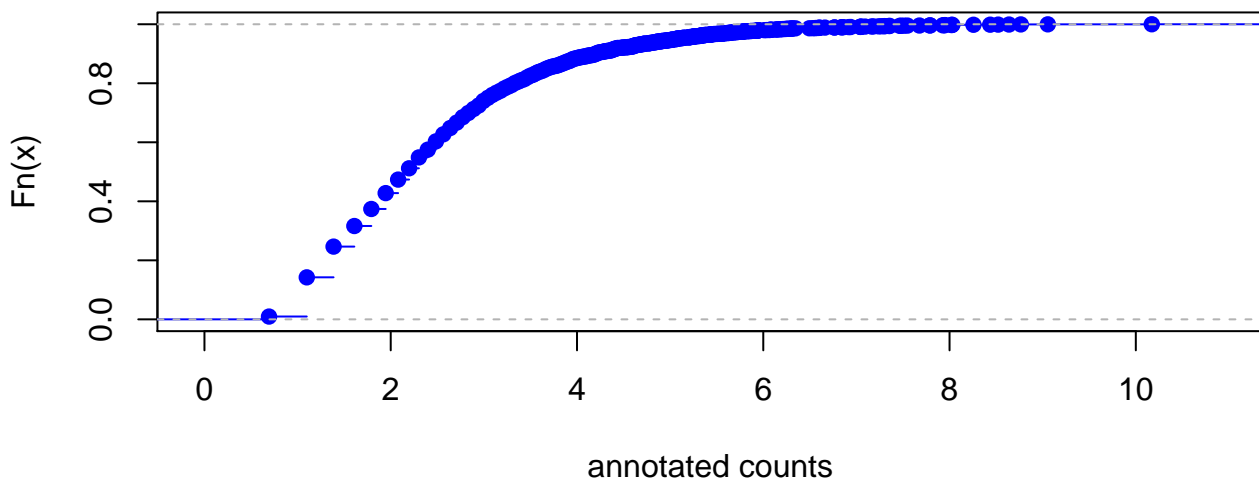

Supplement: Additional file 22: — Comparison of de novo and annotated circular RNA counts. Many de novo junctions have high expression, comparable to circular RNA expression from RNA spliced at canonical boundaries. We plotted empirical cumulative distributions of total circular counts per gene from all annotated junctions and separately for all de novo junctions after reports had been screened as described in the methods; total expression estimates were collapsed across samples and reported at the gene level. ECDF empirical cumulative distribution function. [file 13059_2015_690_MOESM22_ESM.pdf]

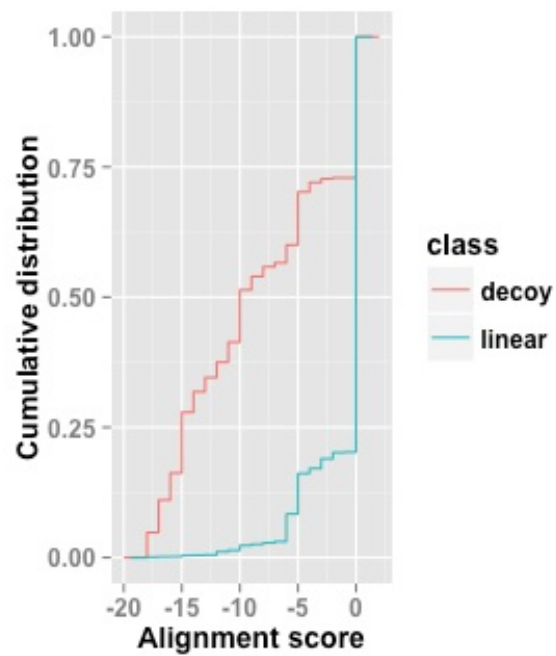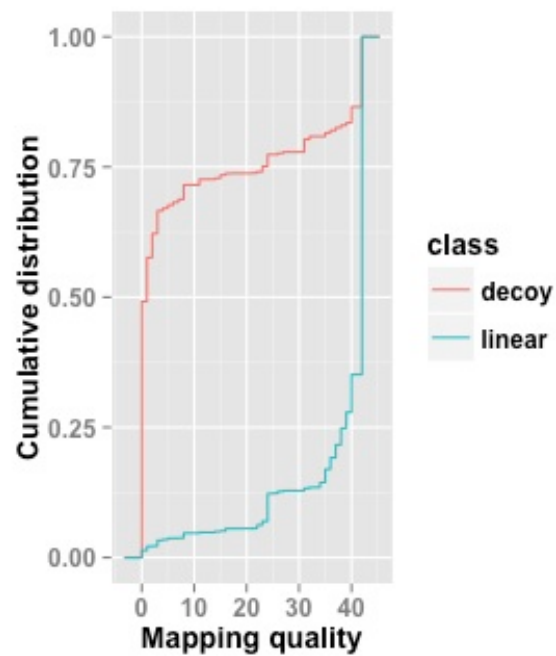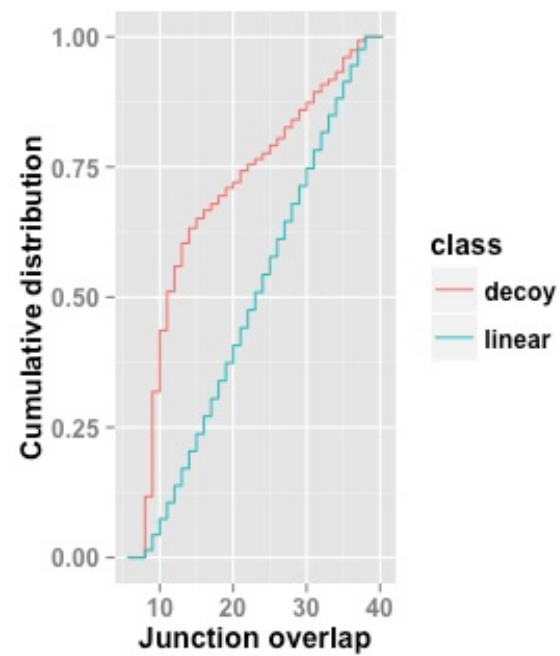

Supplement: Additional file 25: — Cumulative distribution of predictors used in GLM. Bowtie2 alignment score, mapping quality, and the amount of junction overlap are distributed differently in the two categories of reads used to fit the model: 1) that map to canonical linear isoforms (real alignments); 2) that are likely artifacts because their relative alignment orientations are inconsistent with coming from a linear or circular RNA (decoy alignments). [file 13059_2015_690_MOESM25_ESM.pdf]
