# Supplementary material for: Statistically based splicing detection reveals neural enrichment and tissue-specific induction of circular RNA during human fetal development
Source: Genome Biol. 2015 Jun 16;16(1):126. doi: 10.1186/s13059-015-0690-5 (PMC4506483; doi:10.1186/s13059-015-0690-5)
Supplement: Additional file 9: — Primers and sequencing results. [file 13059_2015_690_MOESM9_ESM.doc]

PRIMERS:

***qPCR primers:***

| **gene / isoform** | **sense** | **primer #** | **sequence** |
| --- | --- | --- | --- |
| ASXL1  circle | forward | oWB1380 | CATTCCAATTCAAGAGGAGGAGA |
|  | reverse | oWB1381 | GCCTCTATGACCTGCAGAATC |
| ASXL1  linear | forward | oWB1380 | CATTCCAATTCAAGAGGAGGAGA |
|  | reverse | oWB1382 | CACAGTGCTGGCTTCATTAGAC |
| ATXN10  circle | forward | oWB1436 | CTGCAACATCAGTGACAGTAACC |
|  | reverse | oWB1480 | CAAAGGCTGGTGGAACAGAC |
| ATXN10  linear | forward | oWB1436 | CTGCAACATCAGTGACAGTAACC |
|  | reverse | oWB1479 | CCCTGTTCCTCCATCTTTGC |
| CAMSAP1  circle | forward | oWB1395 | AAGGTGGTGGCCAGTGTCAA |
|  | reverse | oWB1371 | GCTTAATGTGCTCCTGCTCATAC |
| CAMSAP1  linear | forward | oWB844 | GCCAACCTGCAGTGGATC |
|  | reverse | oWB1371 | GCTTAATGTGCTCCTGCTCATAC |
| FAT3  circle | forward | oWB1383 | AGGGATGGAGAGATCCAGTA |
|  | reverse | oWB1384 | GCTGCTGAGTTCTCATACAC |
| FAT3  linear | forward | oWB1383 | AGGGATGGAGAGATCCAGTA |
|  | reverse | oWB1385 | GGGGCATTGTCATTCACATCTTC |
| HIPK3  circle | forward | oWB1379 | CGGCCAGTCATGTATCAAAGAC |
|  | reverse | oWB1377 | GCTTGGCTCTACTTTGAGTTTCTTC |
| HIPK3  linear | forward | oWB1379 | CGGCCAGTCATGTATCAAAGAC |
|  | reverse | oWB1378 | CTGATCATACTCCAAGGCTCCT |
| MORC3  circle | forward | oWB1424 | CACAGCTGGTTTCGAAGAGTC |
|  | reverse | oWB1423 | CAGAGAATGTTCCAGAATTGCAGCA |
| MORC3  linear | forward | oWB1480 | GCGGAGCATGTTGTTGTTCCA |
|  | reverse | oWB1423 | CAGAGAATGTTCCAGAATTGCAGCA |
| NCX1  circle | forward | oWB1372 | GTGGAGAGCTCGAATTCCAGA |
|  | reverse | oWB1373 | TGGGGTTCCCAAATGGGCAA |
| NCX1  linear | forward | oWB1374 | CACTTTGGCTGCACCATTGG |
|  | reverse | oWB1375 | CGATTCCCAGGAAGACATTCAC |
| RANBP17  circle | forward | oWB1417 | GAACACAAAGACCAGCTACACCA |
|  | reverse | oWB1419 | GCATGAATTTCACAGCATCTATCTTC |
| RANBP17  linear | forward | oWB1481 | CTGGGGAAGATATGAGCCTGT |
|  | reverse | oWB1419 | GCATGAATTTCACAGCATCTATCTTC |
| RHOBTB3  circle | forward | oWB1386 | CAGGTGCTTTTCAGTGGGAAGA |
|  | reverse | oWB1387 | TCCACACACTGGCAGCAGA |
| RHOBTB3  circle | forward | oWB1386 | CAGGTGCTTTTCAGTGGGAAGA |
|  | reverse | oWB1388 | AGACAACATCGGCAAGCATCG |
| RMST  circle * | forward | oWB1402 | ATTACCCACACGGAGTCAGC |
|  | reverse | oWB1403 | TGATTATATTTCCCCGCAGC |
| RMST  linear | forward | oWB1410 | GGGTTCCATGAGAAGTGACATC |
|  | reverse | oWB1403 | TGATTATATTTCCCCGCAGC |
| SMARCA5  circle | forward | oWB1389 * | GAATGAAAAGCTCTCCAAGATGGG |
|  | reverse | oWB1390 ^ | CAGTCTTCTTTGCACCTCTTTC |
| SMARCA5  linear | forward | oWB1389 * | GAATGAAAAGCTCTCCAAGATGGG |
|  | reverse | oWB1391 | GAGGAAAGAACTGGAAATCCTGAAC |
| SETD3  circle | forward | oWB1392 | GTCTTCAGCCAGTATAAAAACACAGC |
|  | reverse | oWB1410 | CTTTGGTGACACAGTTGCTGTAG |
| SETD3  linear | forward | oWB1392 | GTCTTCAGCCAGTATAAAAACACAGC |
|  | reverse | oWB1394 | ACAGCGGTCATCTTCCAGGTT |
| TCEA3  circle | forward | oWB1414 | GCAACTCCAATCCTGGTTGTCT |
|  | reverse | oWB1416 | CAAGGACTATGGAGTCAACTGTG |
| TCEA3  linear | forward | oWB1482 | AGGAAGAGGAGCTGCTGAG |
|  | reverse | oWB1416 | CAAGGACTATGGAGTCAACTGTG |
| ZWILCH  circle | forward | --- | ACGGTAGCCTGGAAGAAAGG |
|  | reverse | --- | TGAAAGGCATGCAACTGACT |
| ZWILCH  linear | forward | --- | TGGAACCATGGACACAGTTT |
|  | reverse | --- | TGAAAGGCATGCAACTGACT |
| ACTB  linear | forward | oWB1210 | GGCATCCTCACCCTGAAGTA |
|  | reverse | oWB1211 | AGAGGCGTACAGGGATAGCA |
| HMBS  linear | forward | oWB792 | CTGCAGTTTGAAATCATTGCTATGTC |
|  | reverse | oWB793 | GAGGTTTCCCCGAATACTCCT |

* RMST circle primers are the same as the trans-splicing primers used in [Wu CS *et al.*, *Genome Res.* 2014 24:25-36].

***PCR primers for cloning / sequencing:***

These are all outward-facing primers within a single exon, so able to capture most of the sequence of circles containing that exon.

| **gene / isoform** | **sense** | **primer #** | **sequence** |
| --- | --- | --- | --- |
| ATXN10  circle | forward | oWB1436 | CTGCAACATCAGTGACAGTAACC |
|  | reverse | oWB1437 | CCAGGATCAACGGGATACCA |
| MORC3  circle | forward | oWB1422 | CGGAACAGAAGTTACTGGCAGA |
|  | reverse | oWB1423 | CAGAGAATGTTCCAGAATTGCAGCA |
| RANBP17  circle | forward | oWB1417 | GAACACAAAGACCAGCTACACCA |
|  | reverse | oWB1418 | TCAGTGCAAAGGCAATCCCT |
| TCEA3  circle | forward | oWB1414 | GCAACTCCAATCCTGGTTGTCT |
|  | reverse | oWB1415 | TAATGGGGTCCGCAAGCAC |

SEQUENCING RESULTS:

***SLC8A1 (NCX1)***

Direct Sanger sequencing of several qPCR products from fetal tissues showed mixed peaks beginning at the point of the alternative cricular splice site identified from RNA-Seq analysis; this alternative splice gives a circle with a 3 nt deletion compared to the major circle isoform that splices to the annotated 5' end of exon1. The alternative isoform peaks are 10-20% of the height of the major isoform.


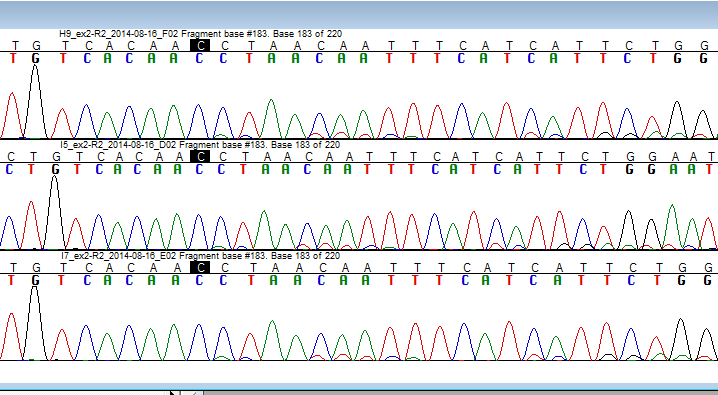


This was further analyzed by TOPO-cloning and sequencing a qPCR product from fetal heart.

1 of 12 clones showed the sequence of the alternative circle. The sequence of that clone, #13, is shown below, with ':::' marking the 3 nt deletion.

The sequence of a representative major isoform clone, #1, is also shown.

**>NCX1#13**

GTGGAGAGCTCGAATTCCAGAATGATGAAATTGT:::GTTGTGACAGTTGGAAGTGTCATGTACAACATGCGGCGATTAAGTCTTTCACCCACCTTTTCAATGGGATTTCATCTGTTAGTTACTGTGAGTCTCTTATTTTCCCATGTGGACCATGTAATTGCTGAGACAGAAATGGAAGGAGAAGGAAATGAAACTGGTGAATGTACTGGATCATATTACCGTAAGA

**>NCX1#1**

GTGGAGAGCTCGAATTCCAGAATGATGAAATTGTTAGGTTGTGACAGTTGGAAGTGTCATGTACAACATGCGGCGATTAAGTCTTTCACCCACCTTTTCAATGGGATTTCATCTGTTAGTTACTGTGAGTCTCTTATTTTCCCATGTGGACCATGTAATTGCTGAGACAGAAATGGAAGGAGAAGGAAATGAAACTGGTGAATGTACTGGATCATATTACTGTAAGA

***MORC3***

These Sanger sequences derive from TOPO-clones from RT-PCR of fetal heart.

**>MORC#14**

CGGAACAGAAGTTACTGGCAGAACTTGATGCTATTATAGGCAAGAAGGGGACGAGGATCATCATTTGGAATCTTAGAAGCTACAAAAATGCAACAGAGTTCGATTTTGAAAGGGATAAATATGATATCAGAATTCCCGAGGATTTAGATGAGATAACAGGGAAGAAGGGGTACAAGAAGCAGGAAAGGATGGACCAGATTGCCCCTGAGAGTGACTATTCCCTGAGGGCTTATTGCAGTATATTATATCTAAAGCCAAGAATGCAGATCATCCTACGTGGACAGAAAGTGAAGACACAGCTGGTTTCGAAGAGTCTTGCCTACATCGAACGTGATGTTTATCGACCAAAATTTTTAATATACGACAGATGATTAATTTAGCAGAATCAAAAGCCAGCCTTGCTGCAATTCTGGAACATTCTCTG

**>MORC#2,4a,6,15**

CGGAACAGAAGTTACTGGCAGAACTTGATGCTATTATAGGCAAGAAGGGGACGAGGATCATCATTTGGAATCTTAGAAGCTACAAAAATGCAACAGAGTTCGATTTTGAAAAGGATAAATATGATATCAGAATTCCCGAGGATTTAGATGAGATAACAGGGAAGAAGGGGTACAAGAAGCAGGAAAGGATGGACCAGATTGCCCCTGAGAGTGACTATTCCCTGAGGGCTTATTGCAGTATATTATATCTAAAGCCAAGAATGCAGATCATCCTACGTGGACAGAAAGTGAAGACACAGCTGGTTTCGAAGAGTCTTGCCTACATCGAACGTGATGTTTATCGACCAAAATTTTTAACGACAGATGATTAATTTAGCAGAATCAAAAGCCAGCCTTGCTGCAATTCTGGAACATTCTCTG

**>MORC#4b,5,8**

CGGAACAGAAGTTACTGGCAGAACTTGATGCTATTATAGGCAAGAAGGGGACGAGGATCATCATTTGGAATCTTAGAAGCTACAAAAATGCAACAGAGTTCGATTTTGAAAAGGATAAATATGATATCAGAATTCCCGAGGATTTAGATGAGATAACAGGGAAGAAGGGGTACAAGAAGCAGGAAAGGATGGACCAGATTGCCCCTGAGAGTGACTATTCCCTGAGGGCTTATTGCAGTATATTATATCTAAAGCCAAGAATGCAGATCATCCTACGTGGACAGAAAGTGAAGACACAGCTGGTTTCGAAGAGTCTTGCCTACATCGAACGTGATGTTTATCGACCAAAATTTTTAACAGATGATTAATTTAGCAGAATCAAAAGCCAGCCTTGCTGCAATTCTGGAACATTCTCTG

**>MORC#1**

CGGAACAGAAGTTACTGGCAGAACTTGATGCTATTATAGGCAAGAAGGGGACGAGGATCATCATTTGGAATCTTAGAAGCTACAAAAATGCAACAGAGTTCGATTTTGAAAAGGATAAATATGATATCAGAATTCCCGAGGATTTAGATGAGATAACAGGGAAGAAGGGGTACAAGAAGCAGGAAAGGATGGACCAGATTGCCCCTGAGAGTGACTATTCCCTGAGGGCTTATTGCAGTATATTATATCTAAAGCCAAGAATGCAGATCATCCTACGTGGACAGAAAGTGAAGACACAGCTGGTTTCGAAGAGTCTTGCCTACATCGAACGTGATGTTTATCGACCAAAATTTTTAATGATTAATTTAGCAGAATCAAAAGCCAGCCTTGCTGCAATTCTGGAACATTCTCTG

***RANBP17***

These Sanger sequences derive from TOPO-clones from RT-PCR of fetal heart.

**>RANBP17#9**

GAACACAAAGACCAGCTACACCATGCTGTTTGACTGGATaTGCTGTGAAATTCATGCTAAAAAACCACACGAGTGAACACTTCCCTTTTCTTGGCATCAGTGACAATCATAGTCTCAGCGACTTCAGGTGTCGAACAACCTTCTACACAGCGCTCACTCGCCTTCTGATGGTAGATCTGGGTGAAGATGAGGATGAATTTGAGAATTTCATGCTGCCTCTTACAGTTGCTTTTGAAACAGTATTACAAATATTCAACAACAACTTTAAACAAGAAGATGTAAAGCGTATGTTGATCGGGCTGGCAAGAGATCTTCGAGGGATTGCCTTTGCACTGA

**>RANBP17#11,12,20**

GAACACAAAGACCAGCTACACCATGCTGTTTGACTGGATaTAGATGCTGTGAAATTCATGCTAAAAAACCACACGAGTGAACACTTCCCTTTTCTTGGCATCAGTGACAATCATAGTCTCAGCGACTTCAGGTGTCGAACAACCTTCTACACAGCGCTCACTCGCCTTCTGATGGTAGATCTGGGTGAAGATGAGGATGAATTTGAGAATTTCATGCTGCCTCTTACAGTTGCTTTTGAAACAGTATTACAAATATTCAACAACAACTTTAAACAAGAAGATGTAAAGCGTATGTTGATCGGGCTGGCAAGAGATCTTCGAGGGATTGCCTTTGCACTGA

**>RANBP17#4,5**

GAACACAAAGACCAGCTACACCATGCTGTTTGACTGGATaTCCTTTTAAAAAAACTTGTGAAGATAGATGCTGTGAAATTCATGCTAAAAAACCACACGAGTGAACACTTCCCTTTTCTTGGCATCAGTGACAATCATAGTCTCAGCGACTTCAGGTGTCGAACAACCTTCTACACAGCGCTCACTCGCCTTCTGATGGTAGATCTGGGTGAAGATGAGGATGAATTTGAGAATTTCATGCTGCCTCTTACAGTTGCTTTTGAAACAGTATTACAAATATTCAACAACAACTTTAAACAAGAAGATGTAAAGCGTATGTTGATCGGGCTGGCAAGAGATCTTCGAGGGATTGCCTTTGCACTGA

**>RANBP17#2,10**

GAACACAAAGACCAGCTACACCATGCTGTTTGACTGGATGTACCCAACGTACCTTCCCCTTCTTCAGAATGCTGTTGAACGGTGGTATGGAGAGCCAACATGTACAACTCCCATCTTGAAACTTATGGCAGAACTTATGCAAAACAGATCCCAGCGTTTGAATTTTGATGTATCATCTCCTAATGGAATTCTTCTCTTCAGAGAAGCTAGTAAAATGGTTTGCACTTATgGTGAAGATGAGGATGAATTTGAGAATTTCATGCTGCCTCTTACAGTTGCTTTTGAAACAGTATTACAAATATTCAACAACAACTTTAAACAAGAAGATGTAAAGCGTATGTTGATCGGGCTGGCAAGAGATCTTCGAGGGATTGCCTTTGCACTGA

***TCEA3***

This derives from direct Sanger sequencing of purified RT-PCR product from fetal heart.

**>TCEA3**

CGCAAGCACTGCTCAGACAAGGAGGTGGTGTCCTTGGCCAAAGTCCTTATCAAAAACTGGAAGCGGCTGCTAGACTCCCCTGGACCCCCAAAAGGAGAAAAAGGAGAGGAAAGAGAAAAGGCAAAGAAGAAGGAAAAAGGGCTTGAGTGTTCAGACTGGAAGCCAGAAGCAGGCCTTTCTCCACCAAGGAAAAAACGAGAAGACCCCAAAACCAGGAGAGACTCTGTGGACTCCAAGTCTTCTGCCTCCTCCTCTCCAAAAAGACCATCGGTGGAAAGATCAAACAGCAGCAAATCAAAAGCGGAGAGCCCCAAAACACCTAGCAGCCCCTTGACCCCCACGTTTGCCTCTTCCATGTGTCTCCTGGCCCCCTGCTATCTCACAGGGGACTCTGTCCGGGACAAGTGTGTGGAGATGCTGTCAGCAGCCCTGAAGGCGGACGATGATTACAAGGACTATGGAGTCAACTGTGACAAGATGGCATCAGAAATCGAAGATCTTTGCAGACAACCAGGTTGGGGAGTTGC

***ATXN10***

These Sanger sequences derive from TOPO-clones of RT-PCR product from the neuroblastoma cell line SH-SY5Y. They show a remarkable complexity of circular isoforms from the ATXN10 gene, including cryptic exons not currently annotated.

**>ATXN10#4.3,4.14**

CTGCAACATCAGTGACAGTAACCCCTCTCTTCAATCTTTCTGTGACAAATGACTAGAATTCTGGAGCCTGCACCTTCATTTTAGGCAGCTATTGCTTCAGAAGTCACAGTCTGTTCCACCAGCCTTTGCCAAGGATGCAGGTGCCACAGCATCTCAAGAGAAGTCACCAATCACACAGCTCCATGCTGAGGCCCTCTTGCCTGCTACTGAGGAGCTCTTGCCACCCCCGATTCCTGCCTTTCCCCAGTGATGCAGGACAGGTAAATGAGCTGGATGGTATCCCGTTGATCCTGG

**>ATXN10#2.3**

CTGCAACATCAGTGACAGTAACCCCTGTTCTGTGAGGTACACTGGATGAAGAAAGGCAGGATAAGTGCTCGATTGTTTTCCCTCTTGATTACCAATCTTCAGAATAATGAGTTGGTTTTGTGGCATCATTCAAAGATGACCAGAGGCACTGGCTACAATTAGGCTTCTCGACGTCCTGTGCGAAATGACTGTGAATACTGAGCTGCTCGGCTATCTGCAGGTTTTCCCTGGCTTGCTGGAAAGAGTGATTGATCTTTTGCGGGTGATTCATGTAGCTGGAAAAGAAACCACAAACATCTTCAGTAATTGTGGTTGCGTGAGAGCAGAAGGTGACATCTCCAATGTGGCCAATGGGTTTAAGTCTCATCTCATTCGTCTGATTGGAAATCTGTGTTACAAGAATAAAGATAACCAAGACAAGGTAAATGAGCTGGATGGTATCCCGTTGATCC

**>ATXN10#4.13**

CTGCAACATCAGTGACAGTAACCCCTTTCTGACCCAGTGGGTGATATATGCCATCCGAAACCTTACCGAAGACAACAGCCAAAACCAAGATTTGATTGCAAAGATGGAGGAACAGGGGCTGGCAGATGCATCCCTACTTAAAAAAGTGGGTTTTGAAGTTGAAAAGAAAGGCGAAAAGCTGATCCTGAAATCTACTAGAGACACCCCTAAGCCATGAATGAACTACATCCAAATACCTGAATTTTTGGAATCTGTTTCATGGATTTTTCATCTTCTACCGTAAATGAGCTGGATGGTATCCCGTTGATCCTGG

**>ATXN10#4.6A**

CTGCAACATCAGTGACAGTAACCCCTCTCTTCAATCTTTCTGTGACAAATGACTAGAATTCTGGAGCCTGCACCTTCATTTTAGGCAGCTATTGCTTCAGAAGTCACAGTCTGTTCCACCAGCCTTTGCCAAGGATGCAGGTGCCACAGCATCTCAAGAGAAGTCACCAATCACACAGCTCCATGCTGAGGCCCTCTTGCCTGCTACTGAGGAGCTCTTGCCACCCCCGATTCCTGCCTTTCCCCAGTGATGCAGGACAGAACAAGCTGAAAGAAAAGCAGGAGGAATATGGAAAACTGGAATACCATCAGCCACCTTGATCAGGAACAAGGCAAGAATTTCTGCTCTCACCACTCCAGTAAACAACGTGCTGAAGGTCCAGAGCAACTCACTAAGCCAAGAAAAATCAGATGCATGCAGATTGGAAAGAAGTAAATGAGCTGGATGGTATCCCGTTGATCCTGG

**>ATXN10#4.6B**

CTGCAACATCAGTGACAGTAACCCCTCTCTTCAATCTTTCTGTGACAAATGACTAGAATTCTGGAGCCTGCACCTTCATTTTAGGCAGCTATTGCTTCAGAAGTCACAGTCTGTTCCACCAGCCTTTGCCAAGGATGCAGGTGCCACAGCATCTCAAGAGAAGTCACCAATCACACAGCTCCATGCTGAGGCCCTCTTGCCTGCTACTGAGGAGCTCTTGCCACCCCCGATTCCTGCCTTTCCCCAGTGATGCAGGACAGAACAAGCTGAAAGAAAAGCAGGAGGAATATGGAAAACTGGAATACCATCAGCCACCTTGATCAGGAACAAGGCAAGAATTTCTGCTCTCACCACTCCAGTAAACAACGTGCTGAAGGTCCAGAGCAACTCACTAAGCCAAGAAAAATCAGATGCATGCAGATTGGAAAGAAGTAAAACTGGCTTTATTCATAAATGAGGTAAATGAGCTGGATGGTATCCCGTTGATCCTGG

**>ATXN10#4.8A**

CTGCAACATCAGTGACAGTAACCCCTGTTCTGTGAGGTACACTGGATGAAGAAAGGCAGGATAAGTGCTCGATTGTTTTCCCTCTTGATTACCAATCTTCAGAATAATGAGTTGGTTTTGTGGCATCATTCAAAGATGACCAGAGGCACTGGCTACAATTAGGCTTCTCGACGTCCTGTGCGAAATGACTGTGAATACTGAGCTGCTCGGCTATCTGCAGGTTTTCCCTGGCTTGCTGGAAAGAGTGATTGATCTTTTGCGGGTGATTCATGTAGCTGGAAAAGAAACCACAAACATCTTCAGTAATTGTGGTTGCGTGAGAGCAGAAGGTGACATCTCCAATGTGGCCAATGGGTTTAAGTCTCATCTCATTCGTCTGATTGGAAATCTGTGTTACAAGAATAAAGATAACCAAGACAAGGTAAATGAGCTGGATGGTATCCCGTTGATCCTGG

**>ATXN10#4.8B**

CTGCAACATCAGTGACAGTAACCCCTGTTCTGTGAGGTACACTGGATGAAGAAAGGCAGGATAAGTGCTCGATTGTTTTCCCTCTTGATTACCAATCTTCAGAATAATGAGTTGGTTTTGTGGCATCATTCAAAGATGACCAGAGGCACTGGCTACAATTAGGCTTCTCGACGTCCTGTGCGAAATGACTGTGAATACTGAGCTGCTCGGCTATCTGCAGGTTTTCCCTGGCTTGCTGGAAAGAGTGATTGATCTTTTGCGGGTGATTCATGTAGCTGGAAAAGAAACCACAAACATCTTCAGTAATTGTGGTTGCGTGAGAGCAGAAGGTGACATCTCCAATGTGGCCAATGGGTTTAAGTCTCATCTCATTCGTCTGATTGGAAATCTGTGTTACAAGAATAAAGATAACCAAGACAAGAACAAGCTGAAAGAAAAGCAGGAGGAATATGGAAAACTGGAATACCATCAGCCACCTTGATCAGGTAAATGAGCTGGATGGTATCCCGTTGATCCTGG

This table summarizes the content of the ATXN10 circles identified by PCR/sequencing:

| **clone** | **insert (bp)** | **description** |
| --- | --- | --- |
| 3.1, 3.8 | 139 | artifact, same primer both ends. |
| 4.3, 4.14 | 294 | in9a / ex10 |
| 2.3 | 452 | in7 / ex8 / ex9 / ex10 |
| 3.7 | 119 | artifact? ex10 / ex11-partial |
| 4.10 | 449 | artifact. same primer both ends, minus and plus strand seq |
| 4.13 | 313 | ex10 / ex11 / ex12-partial(cryptic splice-donor in 3'UTR) |
| 4.6A | 465 | in9a / in9b / in9c / ex10 |
| 4.6B | 492 | in9a / in9b / in9c'(splice-donor distal to 4.6A) / ex10 |
| 4.8A | 455 | in7 / ex8 / ex9 / ex10 |
| 4.8B | 519 | in7 / ex8 / ex9 / in9b / ex10 |

"in" = intron; "ex" = exon; numbering of introns & exons starts from 1. letter suffixes on "in9" denote distinct cryptic exons in intron 9.

***ZWILCH***

These Sanger sequences derive from TOPO-clones of RT-PCR product from the H9 embryonic stem cell line.

Clones #2 and 4 consist of exons 18-15 (RefSeq gene model NM_001287821.1).

Clone #3 consists of exons 18-15-16-18-15, a longer insert presumably resulting from rolling circle reverse transcription, and suggesting the existence of alternatively spliced circular isoforms.

**>ZWILCH_#2,4**

ACGGTAGCCTGGAAGAAAGGATATTCTTTACTAACATGGTTACCTGCAGCCAGGTGCATTTCAAGTGAAGTGTGCTGATGAAGTCCTCTATAAGGAATACTTCATTGCTCCATCAGTAGATATACAAGAACAGGTTTATCGTGTCCAAAAACTCCACCATATTCTAGAAATATTAGTCAGTTGCATGCCTTTCA

**>ZWILCH_#3**

ACGGTAGCCTGGAAGAAAGGATATTCTTTACTAACATGGTTACCTGCAGCCAGGTGCATTTCAAGTGAAGTGTGCTGATGAAGTCCTCTATAAGGAATACTTCATTGCTCCATCAGTAGATATACAAGAACAGGTTTATCGTGTCCAAAAACTCCACCATATTCTAGAAATATTAGTCAGTTGCATGCCTTTCAttaaatctcaacatgaactcctcttttctttaacacagatctgcataaagtattacaaacaaaatcctcttgatgagcaacacatttttcagctgccagtcagaccaactgctgtaaagaacttatatcaaagatttttcggaattaacactaaACGGTAGCCTGGAAGAAAGGATATTCTTTACTAACATGGTTACCTGCAGCCAGGTGCATTTCAAGTGAAGTGTGCTGATGAAGTCCTCTATAAGGgATACTTCATTGCTCCATCAGTAGATATACAAGAACAGGTTTATCGTGTCCAAAAACTCCACCATATTCTAGAAATATTAGTCAGTTGCATGCCTTTCA
