# Supplementary material for: Statistically based splicing detection reveals neural enrichment and tissue-specific induction of circular RNA during human fetal development
Source: Genome Biol. 2015 Jun 16;16(1):126. doi: 10.1186/s13059-015-0690-5 (PMC4506483; doi:10.1186/s13059-015-0690-5)
Supplement: Additional file 13: — Z score plots for each of our fetal samples. For our fetal tissue data, z score plots per organ, leaving out earliest time point to be conservative. Clockwise from top left, histogram of linear z scores, circular z scores, plots of circular z score versus median and maximum linear z score per gene. Linear junctions were used if a splice site was excluded from any splice site used in circular RNA having at least ten counts. Points with most positive z score are labeled for visualization. [file 13059_2015_690_MOESM13_ESM.pdf]

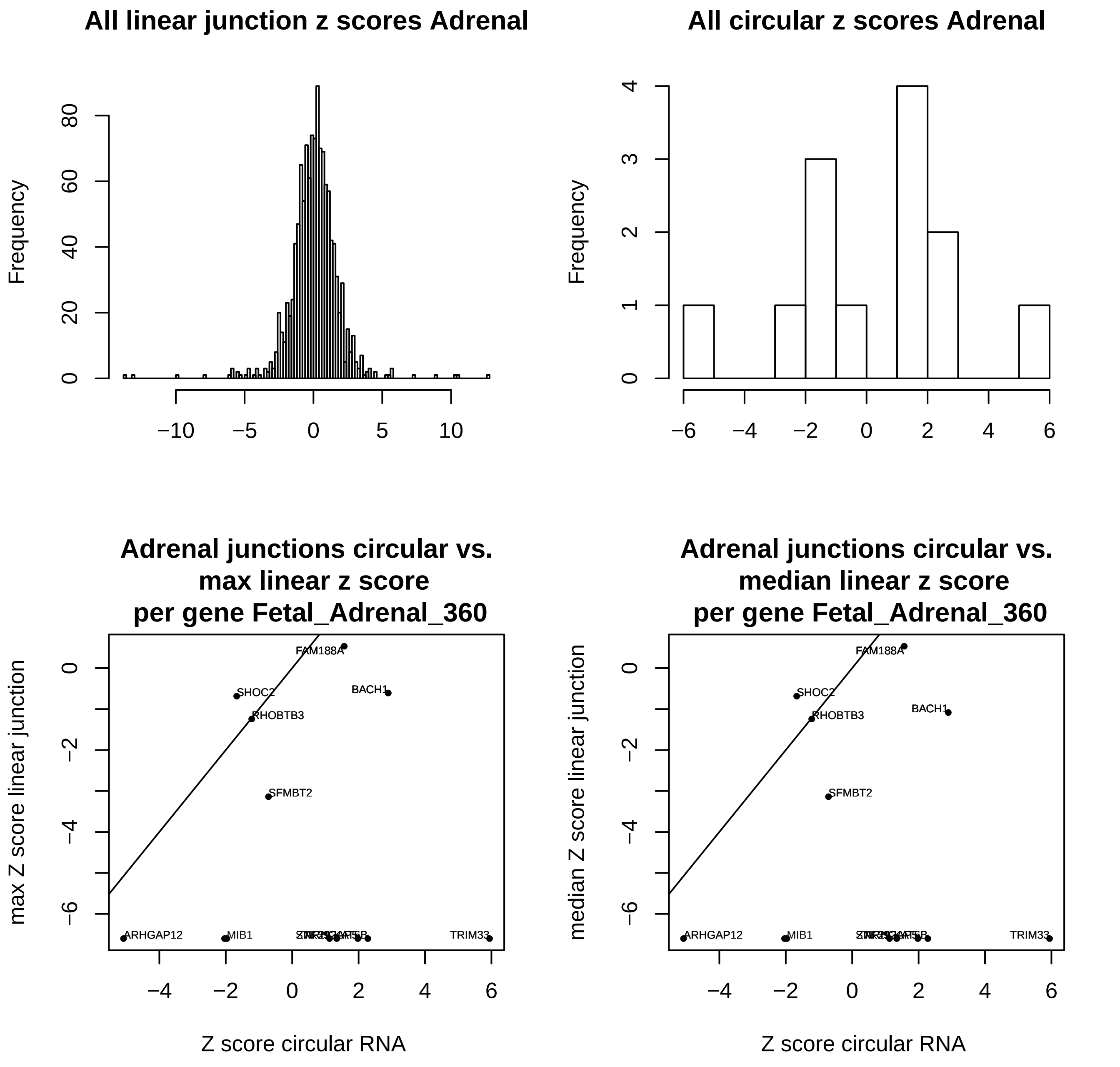

All linear junction z scores Adrenal
Frequency
All circular z scores Adrenal
Frequency
Adrenal junctions circular vs. max linear z score per gene Fetal_Adrenal_360
max Z score linear junction
Z score circular RNA
FAM188A
SHOC2
BACH1
RHOBTB3
SFMBT2
ARHGAP12
MIB1
TRIM33
Adrenal junctions circular vs. median linear z score per gene Fetal_Adrenal_360
median Z score linear junction
Z score circular RNA
FAM188A
SHOC2
RHOBTB3
BACH1
SFMBT2
ARHGAP12
MIB1
TRIM33

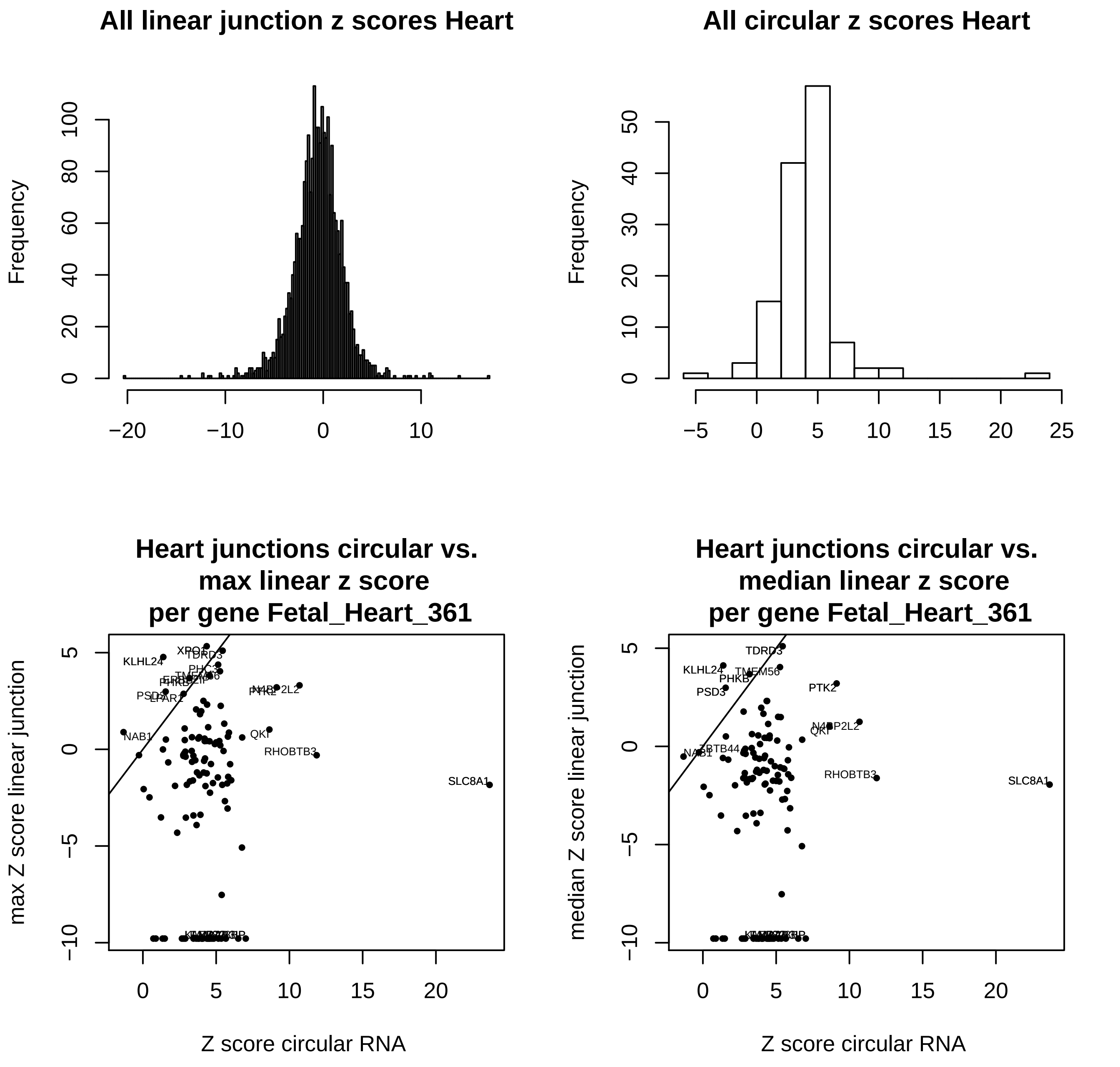
All linear junction z scores Heart
Frequency
0
20
40
60
80
100
−20
−10
0
10
All circular z scores Heart
Frequency
0
10
20
30
40
50
−5
0
5
10
15
20
25
Heart junctions circular vs. max linear z score per gene Fetal_Heart_361
max Z score linear junction
Z score circular RNA
KLHL24
XPO1
TDRD3
PHC3
PSD3
LPAR1
NAB1
QKI
PTK2
RHOBTB3
SLC8A1
−10
−5
0
5
0
5
10
15
20
Heart junctions circular vs. median linear z score per gene Fetal_Heart_361
median Z score linear junction
Z score circular RNA
TDRD3
KLHL24
TMEM56
PHKB
PSD3
PTK2
N4BP2L2
QKI
ZBTB44
NAB1
RHOBTB3
SLC8A1
−10
−5
0
5
0
5
10
15
20

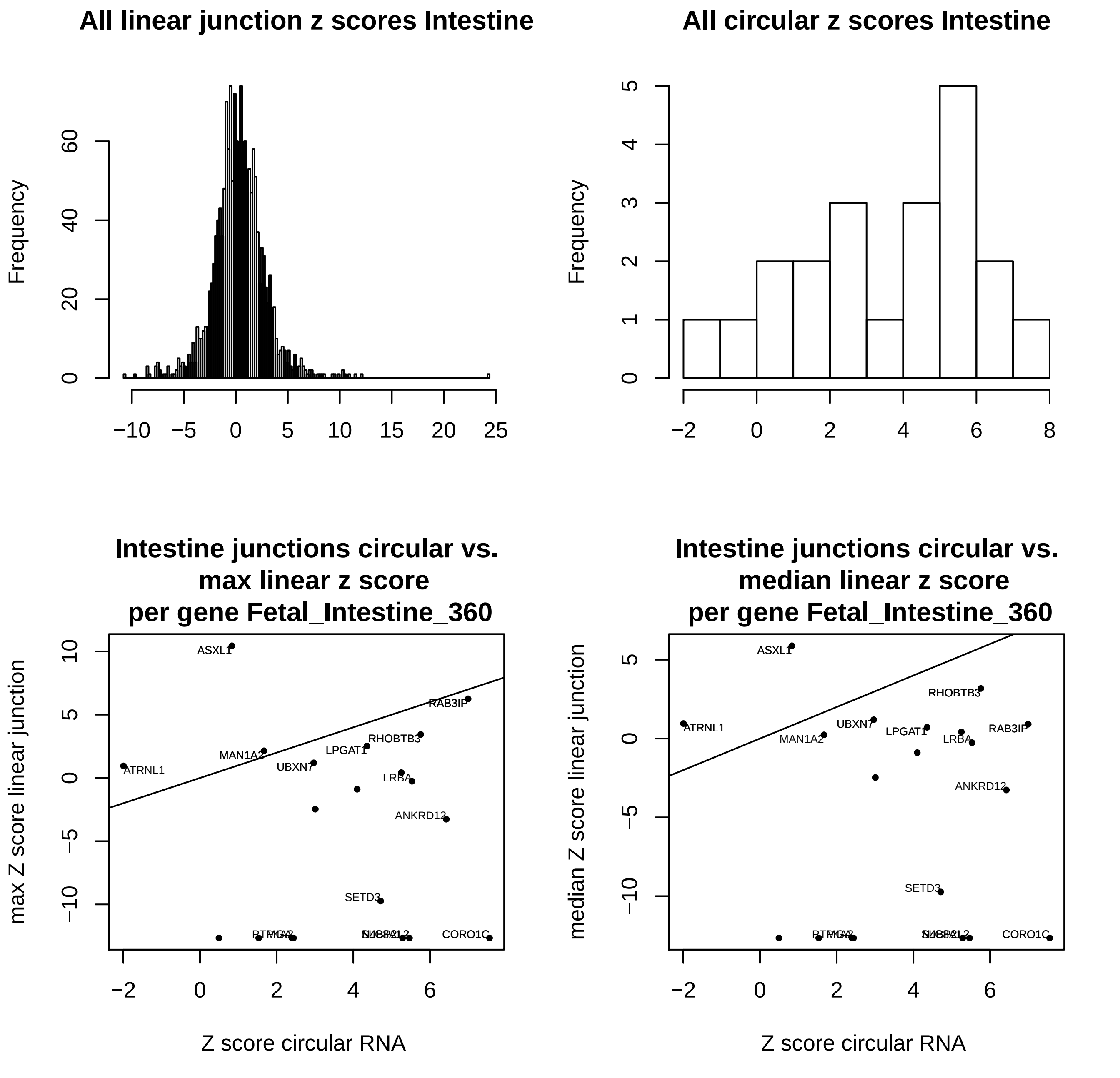

All linear junction z scores Intestine
Frequency
0
20
40
60
−10
−5
0
5
10
15
20
25
All circular z scores Intestine
Frequency
0
1
2
3
4
5
−2
0
2
4
6
8
Intestine junctions circular vs. max linear z score per gene Fetal_Intestine_360
max Z score linear junction
Z score circular RNA
−10
−5
0
5
10
−2
0
2
4
6
ASXL1
RAB3IP
RHOBTB3
MAN1A2
LPGAT1
ATRNL1
UBXN7
LRBA
ANKRD12
SETD3
CORO1C
Intestine junctions circular vs. median linear z score per gene Fetal_Intestine_360
median Z score linear junction
Z score circular RNA
−10
−5
0
5
−2
0
2
4
6
ASXL1
RHOBTB3
ATRNL1
UBXN7
LPGAT1
RAB3IP
MAN1A2
LRBA
ANKRD12
SETD3
CORO1C

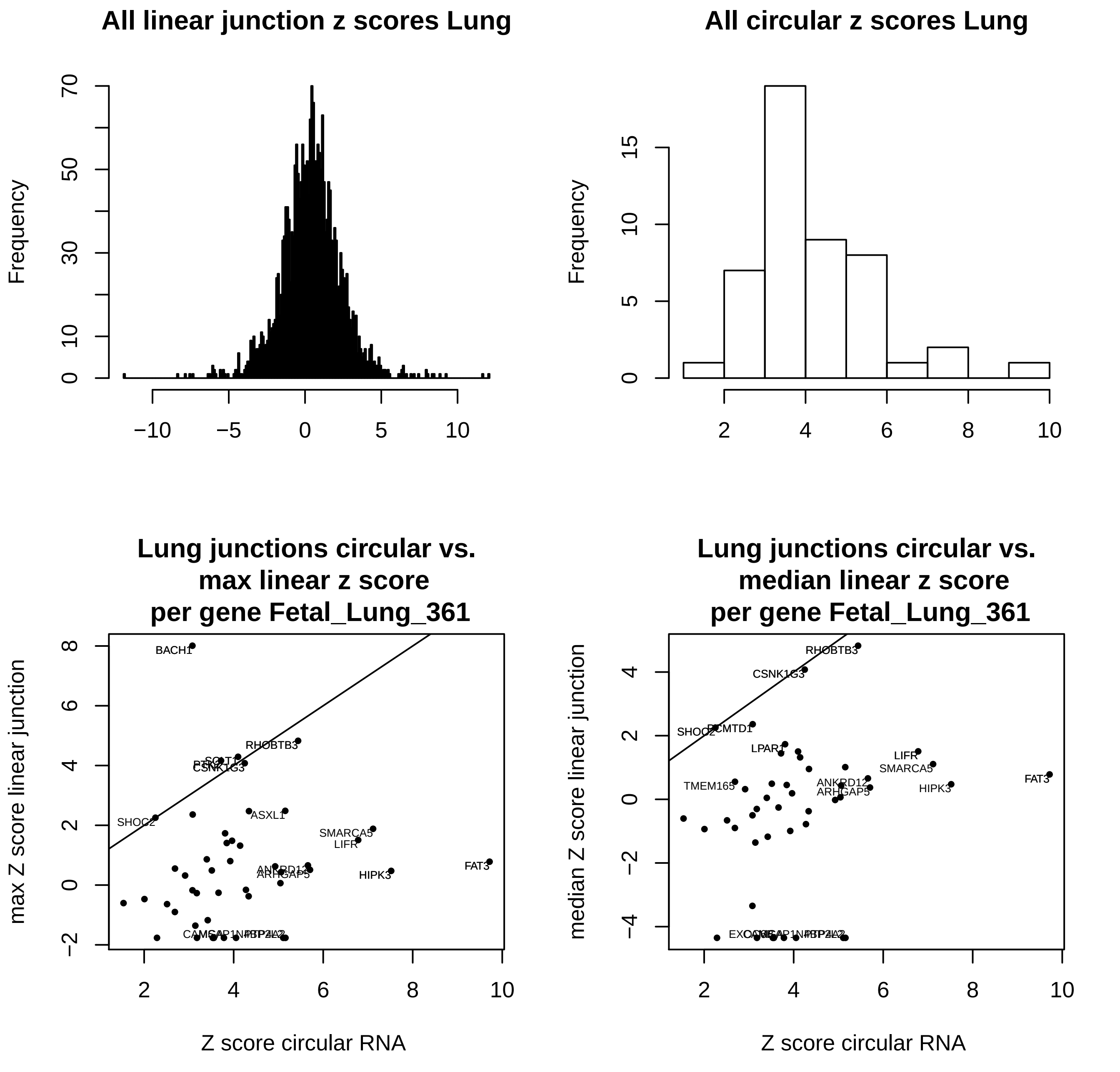
All linear junction z scores Lung
Frequency
0
10
30
50
70
−10
−5
0
5
10
All circular z scores Lung
Frequency
0
5
10
15
2
4
6
8
10
Lung junctions circular vs.
max linear z score
per gene Fetal_Lung_361
max Z score linear junction
−2
0
2
4
6
8
BACH1
RHOBTB3
CSNK1G3
ASXL1
SHOC2
SMARCA5
LIFR
FAT3
HIPK3
ARHGAP5
2
4
6
8
10
Z score circular RNA
Lung junctions circular vs.
median linear z score
per gene Fetal_Lung_361
median Z score linear junction
−4
−2
0
2
4
RHOBTB3
CSNK1G3
SHOC2
LPAR1
LIFR
SMARCA5
FAT3
TMEM165
ANKRD12
ARHGAP5
HIPK3
2
4
6
8
10
Z score circular RNA

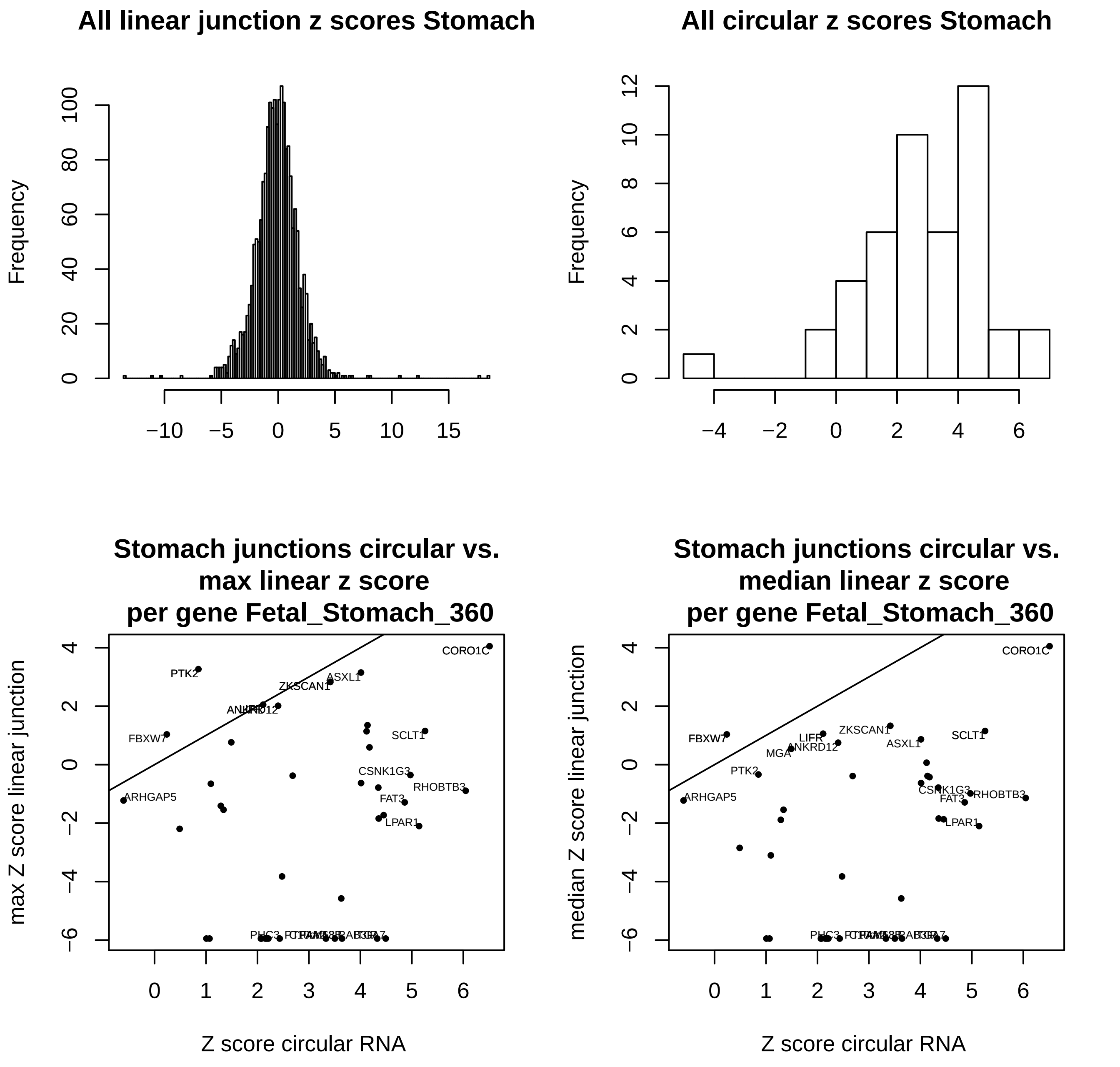

All linear junction z scores Stomach
Frequency
0
20
40
60
80
100
−10
−5
0
5
10
15
All circular z scores Stomach
Frequency
0
2
4
6
8
10
12
−4
−2
0
2
4
6
Stomach junctions circular vs.
max linear z score
per gene Fetal_Stomach_360
max Z score linear junction
Z score circular RNA
CORO1C
PTK2
ASXL1
ZKSCAN1
ANKRD12
FBXW7
SCLT1
CSNK1G3
RHOBTB3
ARHGAP5
FAT3
LPAR1
PHC3
Stomach junctions circular vs.
median linear z score
per gene Fetal_Stomach_360
median Z score linear junction
Z score circular RNA
CORO1C
FBXW7
LIFR
ZKSCAN1
SCLT1
ANKRD12
ASXL1
MGA
PTK2
ARHGAP5
CSNK1G3
RHOBTB3
FAT3
LPAR1
PHC3
