# Supplementary material for: Statistically based splicing detection reveals neural enrichment and tissue-specific induction of circular RNA during human fetal development
Source: Genome Biol. 2015 Jun 16;16(1):126. doi: 10.1186/s13059-015-0690-5 (PMC4506483; doi:10.1186/s13059-015-0690-5)
Supplement: Additional file 17: — Z score plots per tissue for our fetal samples. For our fetal tissue data, z score plots per organ, leaving out earliest time point to be conservative. Clockwise from top left, histogram of linear z scores, circular z scores, plots of circular z score versus median and maximum linear z score per gene. Linear junctions were used if a splice site was shared with any splice site used in circular RNA having at least ten counts. Points with most positive z score are labeled for visualization. [file 13059_2015_690_MOESM17_ESM.pdf]

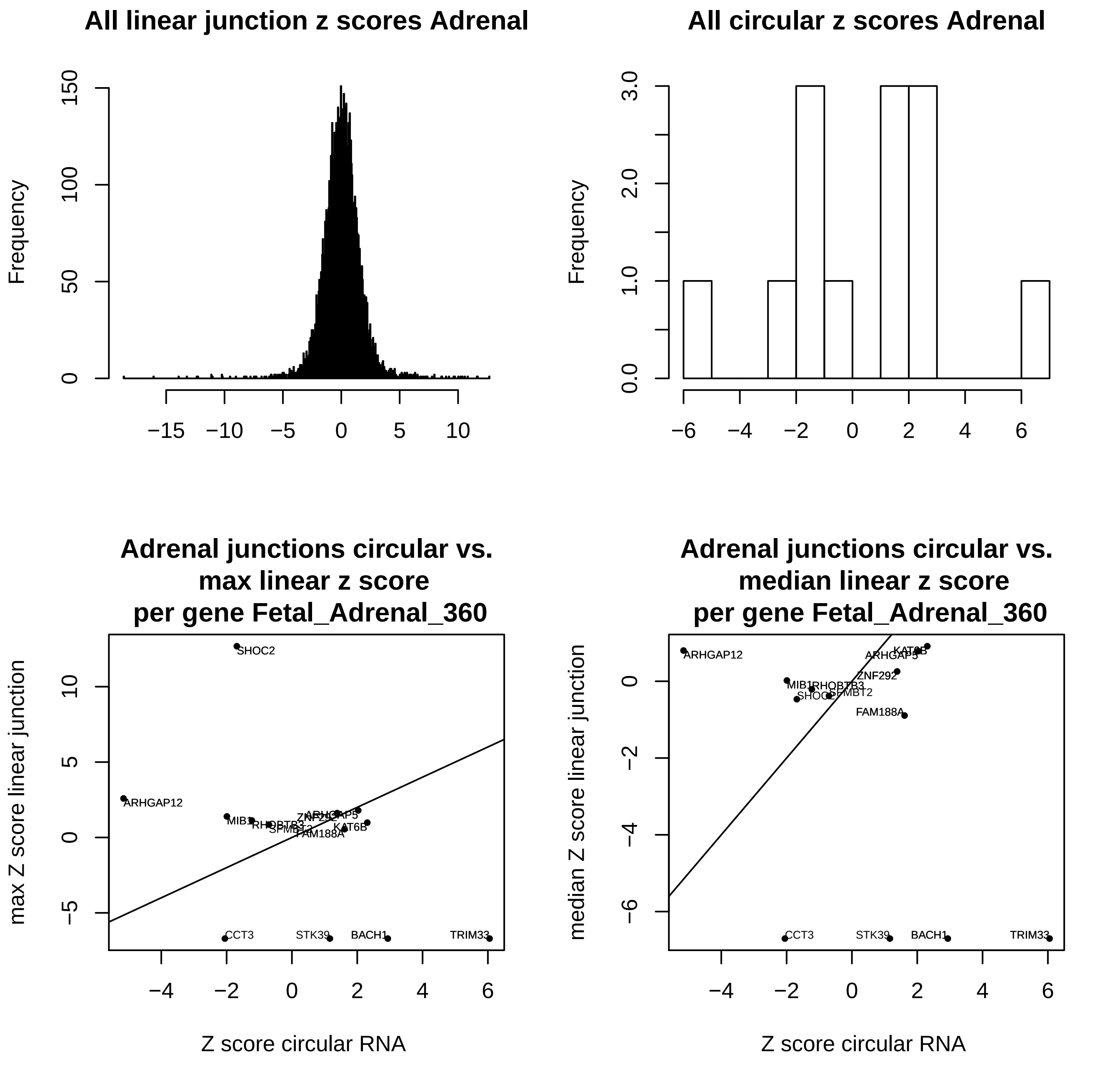
All linear junction z scores Adrenal
Frequency
0
50
100
150
−15
−10
−5
0
5
10
All circular z scores Adrenal
Frequency
0.0
1.0
2.0
3.0
−6
−4
−2
0
2
4
6
Adrenal junctions circular vs. max linear z score per gene Fetal_Adrenal_360
max Z score linear junction
Z score circular RNA
SHOC2
ARHGAP12
MIB1
RHOBTB3
SPMBT2
ZNF292
ARHGAP5
KAT6B
FAM188A
CCT3
STK39
BACH1
TRIM33
−5
0
5
10
−4
−2
0
2
4
6
Adrenal junctions circular vs. median linear z score per gene Fetal_Adrenal_360
median Z score linear junction
Z score circular RNA
ARHGAP12
KAT6B
ARHGAP5
ZNF292
MIB1
RHOBTB3
SHOC2
SPMBT2
FAM188A
CCT3
STK39
BACH1
TRIM33
−6
−4
−2
0
−4
−2
0
2
4
6

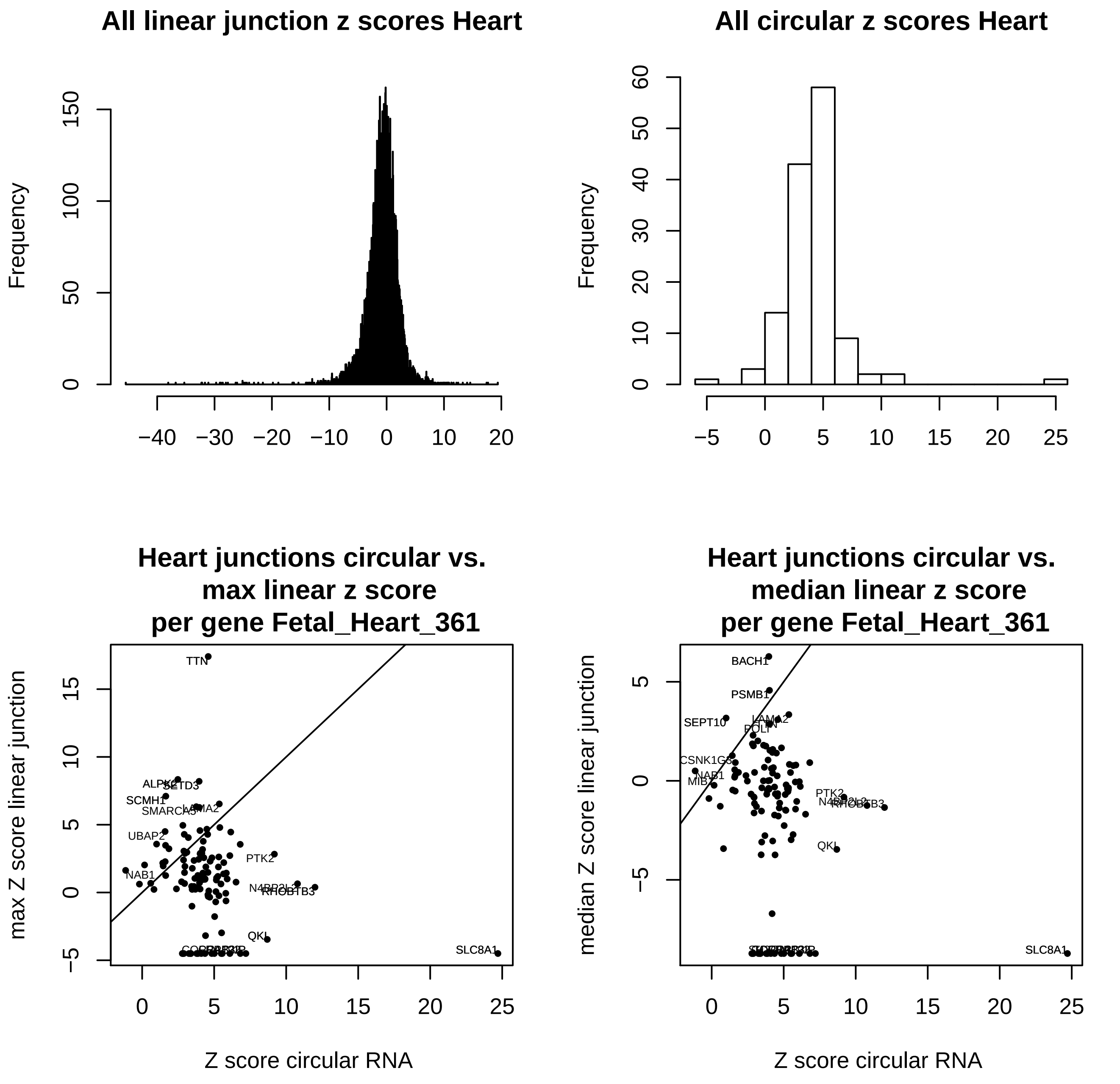
All linear junction z scores Heart
Frequency
All circular z scores Heart
Frequency
Heart junctions circular vs.
max linear z score
per gene Fetal_Heart_361
max Z score linear junction
Z score circular RNA
TTN
SCMH1
UBAP2
NAB1
PTK2
QKI
SLC8A1
Heart junctions circular vs.
median linear z score
per gene Fetal_Heart_361
median Z score linear junction
Z score circular RNA
BACH1
PSMB1
SEPT10
PTK2
QKI
SLC8A1

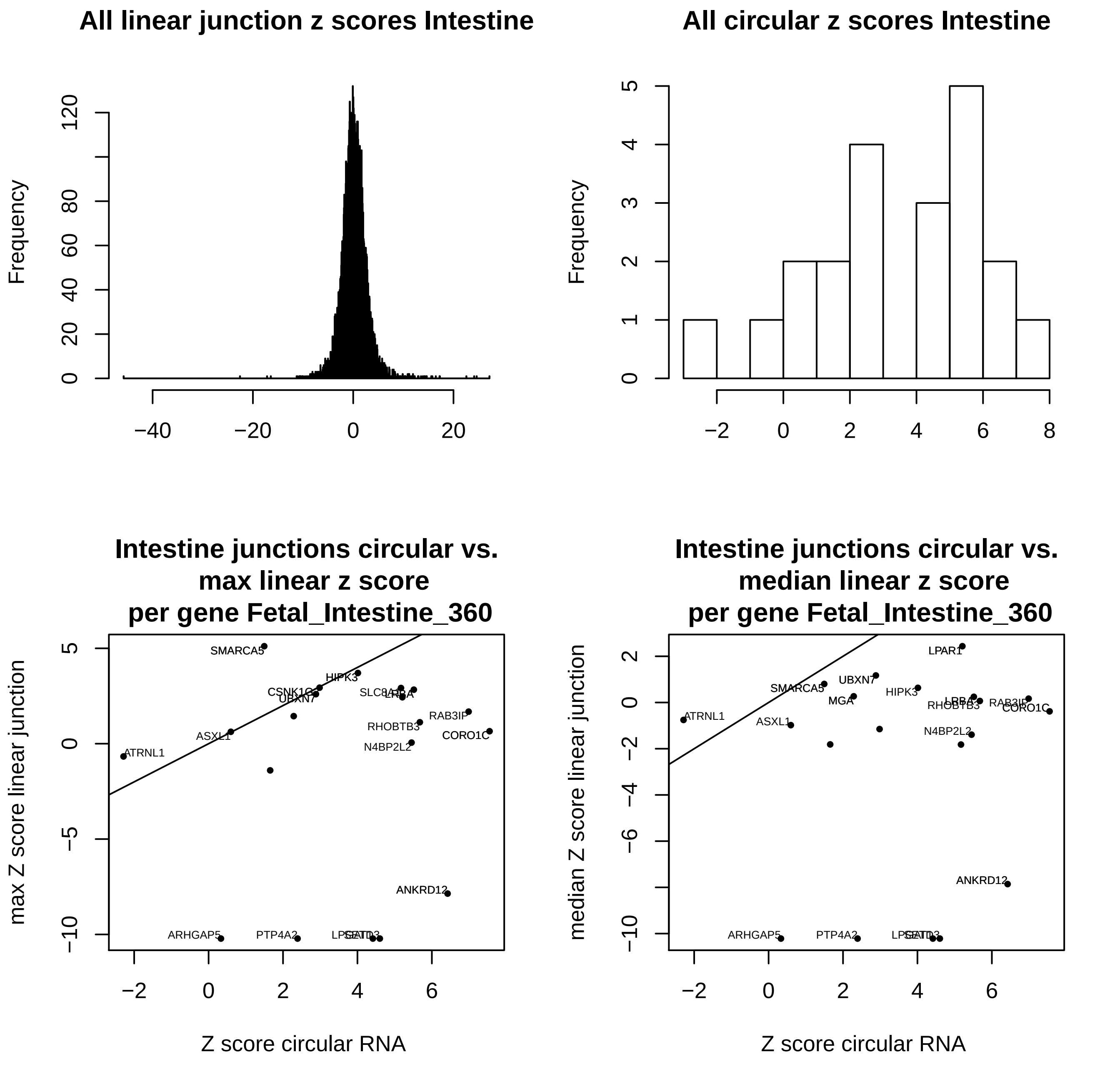
All linear junction z scores Intestine
Frequency
0
20
40
60
80
120
−40
−20
0
20
All circular z scores Intestine
Frequency
0
1
2
3
4
5
−2
0
2
4
6
8
Intestine junctions circular vs.
max linear z score
per gene Fetal_Intestine_360
max Z score linear junction
Z score circular RNA
−10
−5
0
5
−2
0
2
4
6
SMARCA5
HIPK3
CSNK1G3
UBXN7
SLC8A1
LRBA
RAB3IP
RHOBTB3
ASXL1
CORO1C
N4BP2L2
ATRNL1
ANKRD12
ARHGAP5
PTP4A2
Intestine junctions circular vs.
median linear z score
per gene Fetal_Intestine_360
median Z score linear junction
Z score circular RNA
−10
−8
−6
−4
−2
0
2
−2
0
2
4
6
LPAR1
UBXN7
SMARCA5
HIPK3
MGA
LRBA
RAB3IP
RHOBTB3
CORO1C
ATRNL1
ASXL1
N4BP2L2
ANKRD12
ARHGAP5
PTP4A2

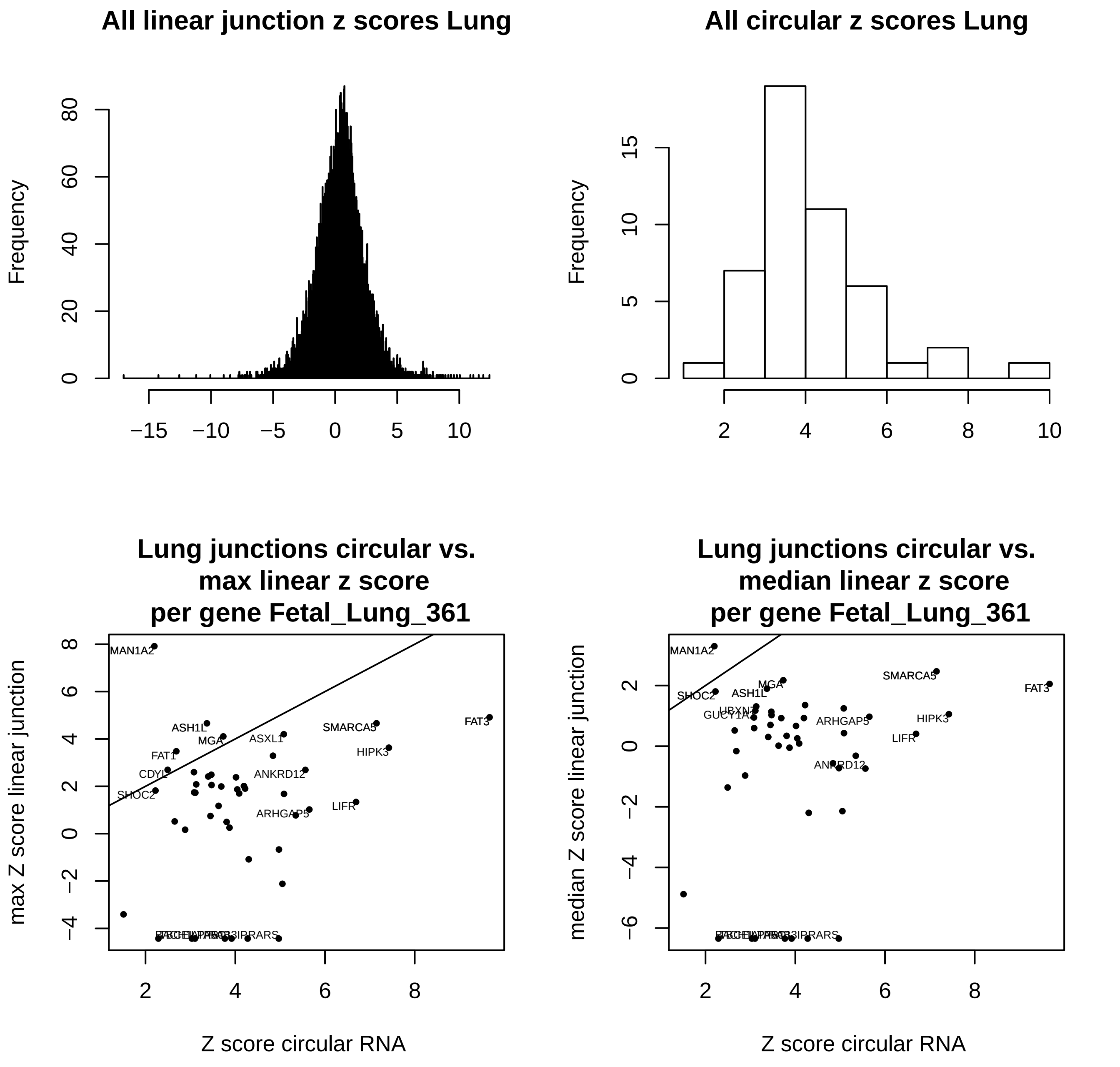
All linear junction z scores Lung
Frequency
0
20
40
60
80
−15
−10
−5
0
5
10
All circular z scores Lung
Frequency
0
5
10
15
2
4
6
8
10
Lung junctions circular vs.
max linear z score
per gene Fetal_Lung_361
max Z score linear junction
Z score circular RNA
MAN1A2
ASH1L
MGA
ASXL1
SMARCA5
FAT3
HIPK3
FAT1
CDYL
ANKRD12
SHOC2
LIFR
ARHGAP5
Lung junctions circular vs.
median linear z score
per gene Fetal_Lung_361
median Z score linear junction
Z score circular RNA
MAN1A2
SMARCA5
MGA
SHOC2
ASH1L
FAT3
GUCY1A2
ARHGAP5
HIPK3
LIFR
ANKRD12

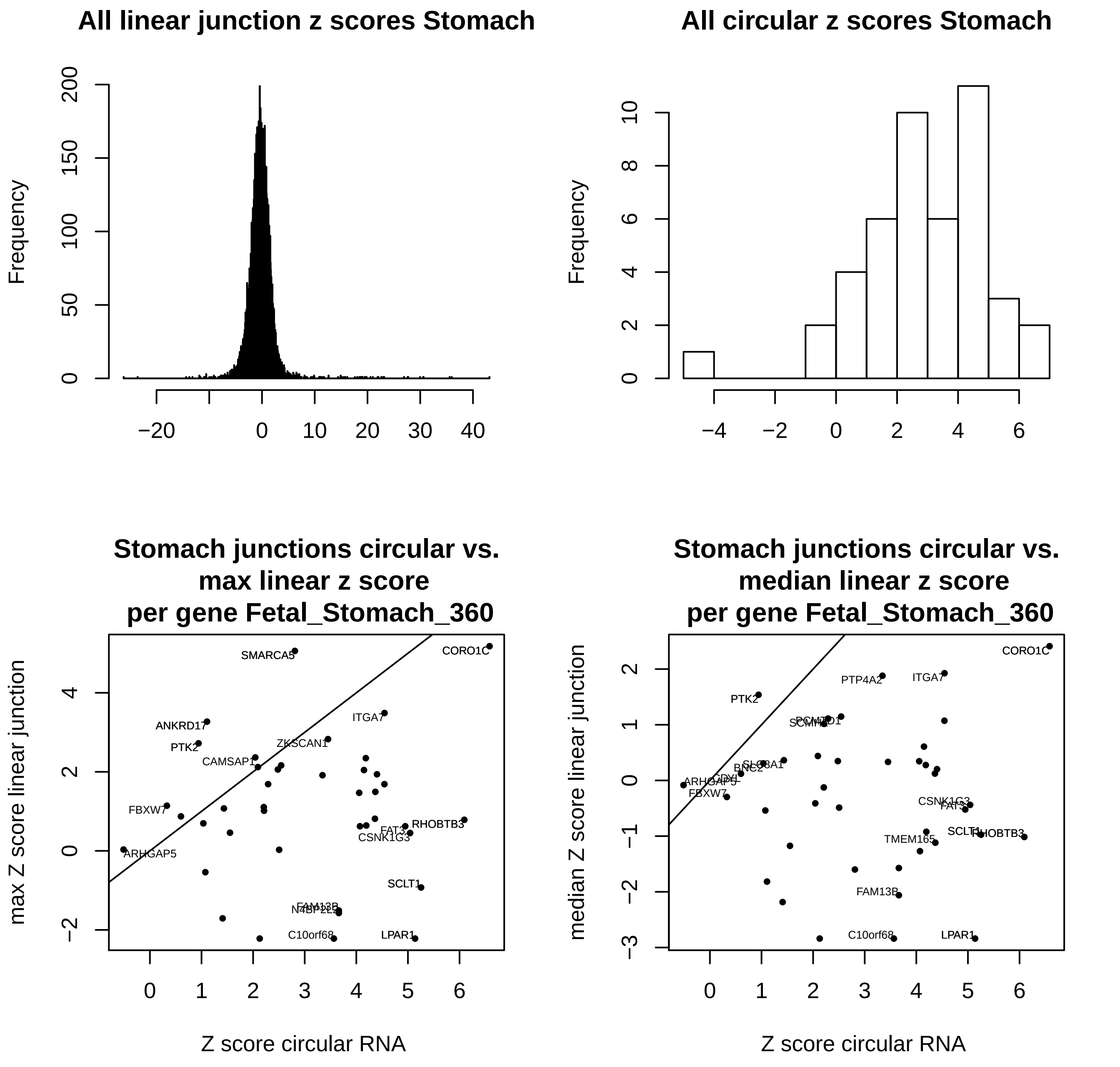
All linear junction z scores Stomach
Frequency
0
50
100
150
200
−20
0
10
20
30
40
All circular z scores Stomach
Frequency
0
2
4
6
8
10
−4
−2
0
2
4
6
Stomach junctions circular vs.
max linear z score
per gene Fetal_Stomach_360
max Z score linear junction
Z score circular RNA
SMARCA5
CORO1C
ANKRD17
ITGA7
PTK2
ZKSCAN1
CAMSAP1
FBXW7
RHOBTB3
FAT3
CSNK1G3
ARHGAP5
SCLT1
FAM13B
N4BP2L2
C10orf68
LPAR1
Stomach junctions circular vs.
median linear z score
per gene Fetal_Stomach_360
median Z score linear junction
Z score circular RNA
CORO1C
PTP4A2
ITGA7
PTK2
PCMTD1
SCMH1
SLC38A1
BNC2
CDYL
ARHGAP5
FBXW7
CSNK1G3
FAT3
SCLT1
RHOBTB3
TMEM165
FAM13B
C10orf68
LPAR1
