# Supplementary material for: Statistically based splicing detection reveals neural enrichment and tissue-specific induction of circular RNA during human fetal development
Source: Genome Biol. 2015 Jun 16;16(1):126. doi: 10.1186/s13059-015-0690-5 (PMC4506483; doi:10.1186/s13059-015-0690-5)
Supplement: Additional file 21: — Z score plots for cardiomyocytes. Clockwise from top left, histogram of linear z scores, circular z scores, plots of circular z score versus median and maximum linear z score per gene. Linear junctions were used if a splice site was shared with any circular RNA having at least one count. Points with most positive z score are labeled for visualization. [file 13059_2015_690_MOESM21_ESM.pdf]

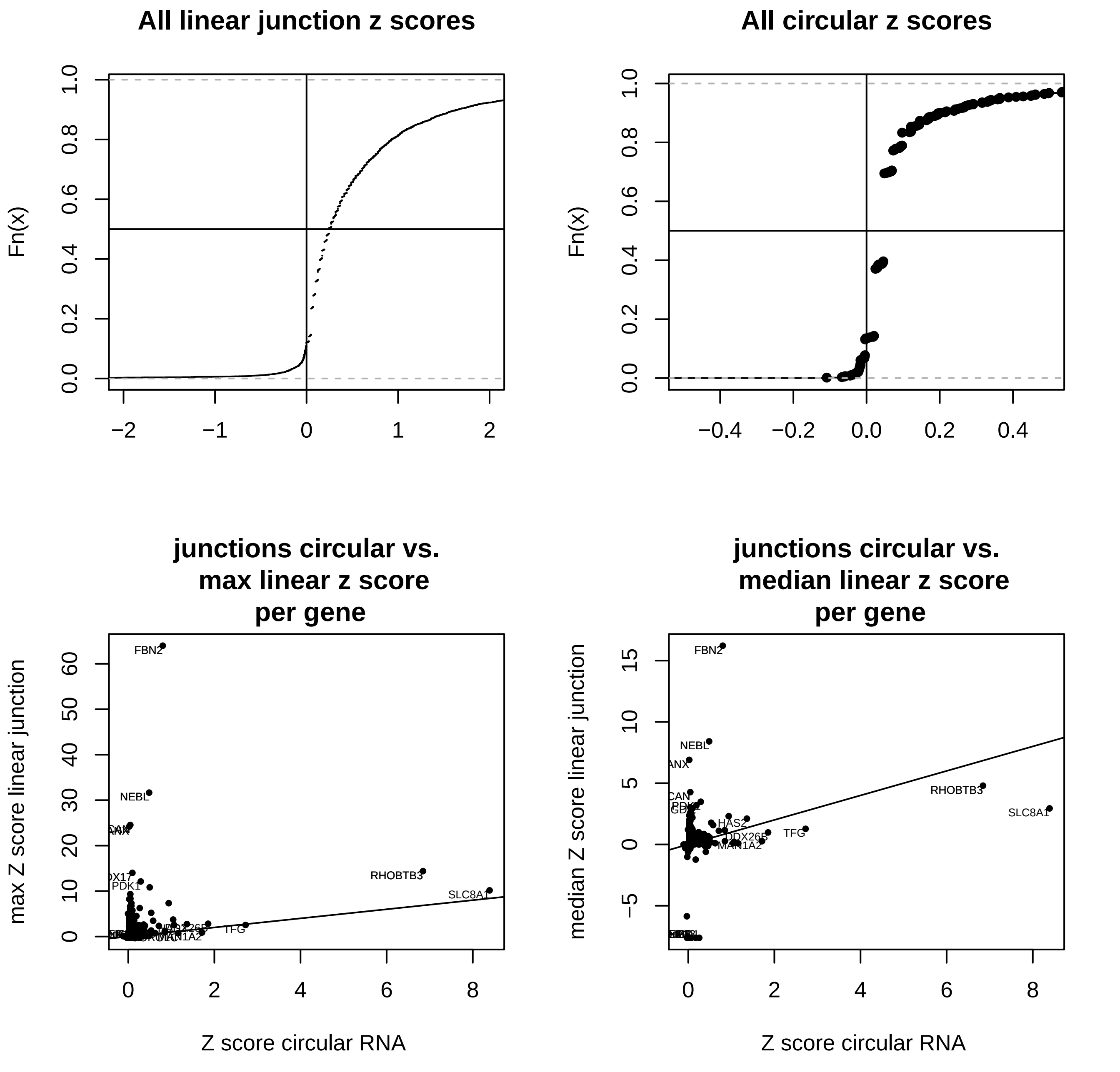
All linear junction z scores
Fn(x)
0.0
0.2
0.4
0.6
0.8
1.0
−2
−1
0
1
2
All circular z scores
Fn(x)
0.0
0.2
0.4
0.6
0.8
1.0
−0.4
−0.2
0.0
0.2
0.4
junctions circular vs.
max linear z score
per gene
max Z score linear junction
0
10
20
30
40
50
60
FBN2
NEBL
PDK1
RHOBTB3
SLC8A1
TFG
MAN1A2
0
2
4
6
8
Z score circular RNA
junctions circular vs.
median linear z score
per gene
median Z score linear junction
−5
0
5
10
15
FBN2
NEBL
PDK1
HAS2
DDX26B
MAN1A2
TFG
RHOBTB3
SLC8A1
0
2
4
6
8
Z score circular RNA
